# Synthetic Analogs of Marine Alkaloid Aplysinopsin Suppress Anti-Apoptotic Protein BCL2 in Prostate Cancer

**DOI:** 10.3390/molecules28010109

**Published:** 2022-12-23

**Authors:** Eslam R. El-Sawy, Zeinab A. El-Shahid, Ahmed A. F. Soliman, Amr Nassrallah, Ahmed B. Abdelwahab, Gilbert Kirsch, Heba Abdelmegeed

**Affiliations:** 1 Chemistry of Natural Compounds Department, National Research Centre, Giza 12622, Egypt; 2 Chemistry of Natural and Microbial Products Department, National Research Centre, Giza 12622, Egypt; 3 Drug Bioassay-Cell Culture Laboratory, Pharmacognosy Department, National Research Center, Giza 12622, Egypt; 4 Biochemistry Department, Faculty of Agriculture, Cairo University, Giza 12613, Egypt; 5 Plant Advanced Technologies (PAT), 54500 Vandœuvre-les-Nancy, France; 6 Laboratoire Lorrain de Chimie Moléculaire (L.2.C.M.), Université de Lorraine, 57050 Metz, France

**Keywords:** aplysinopsin analogs, prostate cancer, anti-apoptotic protein BCL2, cell cycle arrest, molecular docking

## Abstract

Aplysinopsins are a class of indole alkaloids that possess various pharmacological activities. Although their action has been studied in regard to many diseases, their effect on prostate cancer has not yet been examined. Therefore, we synthesized a new series of aplysinopsin analogs and investigated their cytotoxic activity against prostate cancer. Five analogs showed high antitumor activity via suppressing the expression of the anti-apoptotic gene Bcl2, simulationously increasing the expression of the pro-apoptotic genes p53, Bax and Caspase 3. The inhibition of BCL2 led to the activation of BAX, which in turn activated Caspase 3, leading to apoptosis. This dual mechanism of action via apoptosis and cell cycle arrest induction is responsible for aplysinopsin analogs antitumor activity. Hence, our newly synthesized analogs are highly promising candidates for further preclinical studies against prostate cancer.

## 1. Introduction

Prostate cancer is one of the major causes of mortality and morbidity in males [1,2]. Despite recent advances in prostate cancer treatment [3,4], the majority of patients with advanced prostate cancer eventually acquire resistance [5]. Thus, new therapies that target different pathways are important for inhibiting prostate cancer proliferation and increasing the survival rate.

One of the main mechanism for cancer development is apoptosis evasion. BCL2 protein family are involved in apoptosis regulation [6,7]. The overexpression of pro-survival members, such as BCL2, BCL-xL, BCL-w, BCL-B, BCL2A1 and MCL1, is indicative of cancer promotion and metastasis [8]. BCl2 protein is resposnsible for sequestering the pro-apoptotic proteins BAK and BAX, thus inhibiting apoptosis [9]. One of apoptotic mechanisms is the induction of BH3-only proteins which bind to antiapoptotic BCL2 proteins causing the activation of BAX and BAK [10]. The activated BAX and BAK in turn induce apoptosis via activating caspases [11,12]. Caspases are a family of proteases that act as initiator (caspase 8 and caspase 9) or effector proteins (caspase 3, caspase 6 and caspase 7) which cause proteolysis leading to apoptosis via cellular structures degradation and DNA condensation [13]. This coordinated process of apoptosis leads to controlled cell death without affecting neighboring cells.

The marine environment is a superb source of natural bioactive compounds, especially anti-tumor agents [14,15]. In addition to clinical trials, an increasing number of in vivo studies with marine alkaloids were reported to treat tumors [16,17]. Aplysinopsins are a class of marine indole alkaloids that possess a various array of biological activities, for example, antimalarial [18,19], antimicrobial [19,20], monoamine oxidase (MAO) inhibitor [21] and anti-depressant [22]. Recently, it has been shown that aplysinopsins act as a blood–brain barrier permeable scaffold for anti-cholinesterase and anti-BACE-1 activity (beta-site amyloid-precursor protein cleaving enzyme 1) [23]. Additionally, aplysinopsin and its derivatives were found to possess significant anticancer activity against several cancer cell lines, including multidrug resistant (MDR) cell lines and leukemia, breast, colon and uterine cancer cell lines [24,25].

**Scheme 1:**
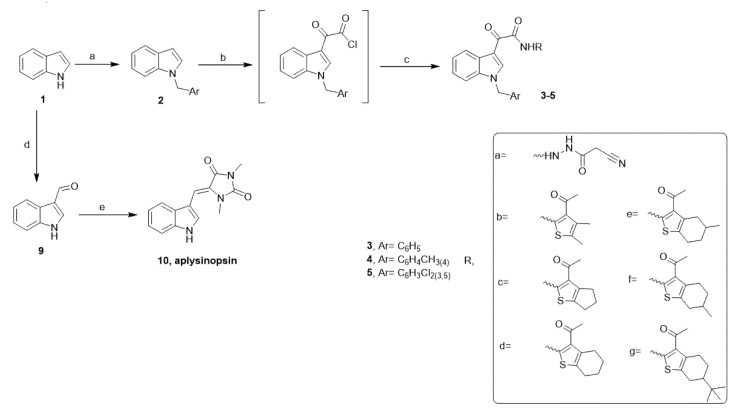
Synthesis of aplysinopsin analogs. Reagents and conditions: (a) benzyl chlorides, NaH, DMF; (b) oxalyl chloride, dry ethyl ether, 40 °C; (c) alkyl or arylamines **a**–**g**, dry THF, TEA, stirring, 3 h; (d) POCl_3_/DMF, 0 °C, NaOH; (e) hydantoin (imidazolidine-2,4-dione), piperidine, reflux, 4 h.

Until recently, the mechanism of action of these compounds as anti-cancer cells was still unclear. Lately, we investigated the mechanism of action of aplysinopsin and some of its synthetic derivatives against leukemia [26]. To further extend our work, we synthesized a new series of aplysinopsin analogs (Scheme 1) and studied their effect on various cancer cell lines. The NMR spectrum data of ^1^H and ^13^C NMR of the new series of aplysinopsin analogs were provided (experimental part and supplementary data). All the synthesized analogs induced high antitumor activity against prostate cancer, while five were the most promising analogs, showing the highest cytotoxic effect. In addition, we evaluated their pro-apoptotic activity and their ability to induce cell cycle arrest. These analogs showed that apoptosis and cell cycle arrest at S-phase are their main mechanisms of action responsible for their cytotoxic activity. This demonstrates that the synthesized aplysinopsin analogs establish a new therapeutic approach against prostate cancer.

## 2. Results

### 2.1. Aplysinopsin Analogs Induced Cytotoxic Activity against Prostate Cancer

The cytotoxic activity aplysinopsin (**10**) and its newly synthesized analogs **3**, **4**, and **5** on different cancer cell lines namely human breast cancer (MCF-7), human colon cancer (HCT-116), human liver cancer (HepG2), Human non-small cell Lung cancer (A549), and human prostate cancer (PC3) were evaluated by in vitro MTT assay. The initial screening assay was at a single dose concentration of (100 μM) for 48 h. The cytotoxicity of the treated cells was determined as cell death % compared to untreated cells. (Supplementary Table S1).

The results indicated that all aplysinopsin analogs under study showed selective cytotoxicity against the PC3 cell line compared with untreated cell. Compounds that showed potent cytotoxic effect (≥ 60%) were subjected to dose response analysis to calculate the half maximal inhibitory concentration (IC_50_) (Supplementary Table S2). The obtained results indicated that all analogs under investigation exhibited selective cytotoxicity against PC3 cell line as compared to untreated cells. Particularly, aplysinopsin (**10**) and its analogs **4b**, **4c**, **4e**, **5a**, and **5b** showed the lowest IC_50_ values. Accordingly, their cytotoxic activity against PC3 cell line was in the increasing order of **4b** (0.037 ± 0.43 μM) > **5a** (0.056 ± 0.3 μM) > **4c** (0.073 ± 0.15 μM) > **4e** (0.075 ± 0.37 μM) > **5b** (0.079 ± 0.24 μM) > aplysinopsin (**10**) (0.107 ± 0.38 μM). Based on these findings, we further studied the cellular mechanisms underlying the potent cytotoxic effect of the analogs **4b**, **4c**, **4e**, **5a**, and **5b** against prostate cancer compared to aplysinopsin (**10**).

### 2.2. Aplysinopsin Analogs Induced Apoptosis in Prostate Cancer

**Figure 1:**
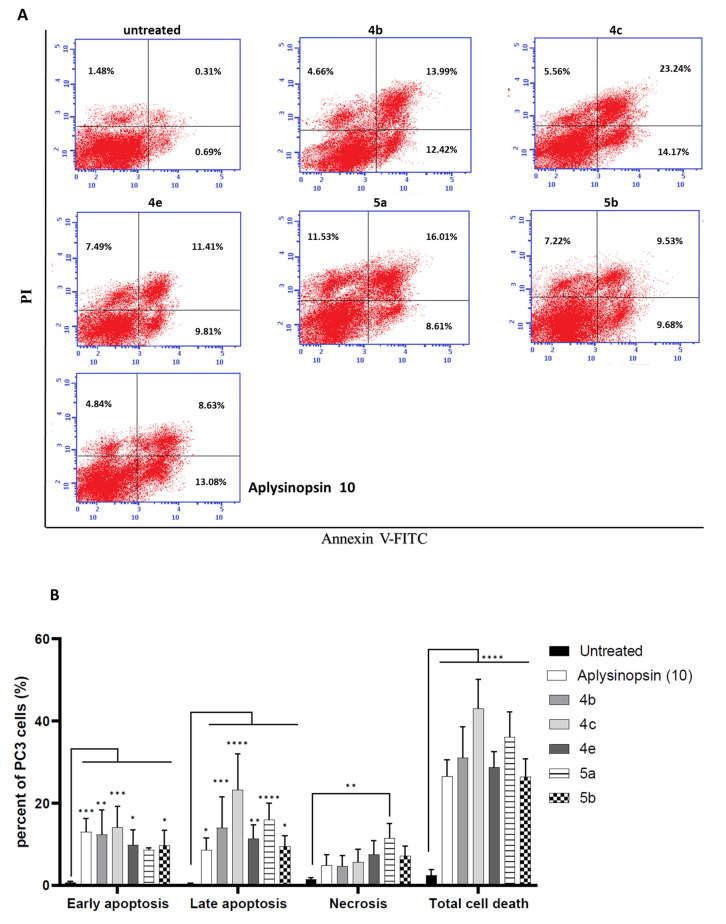

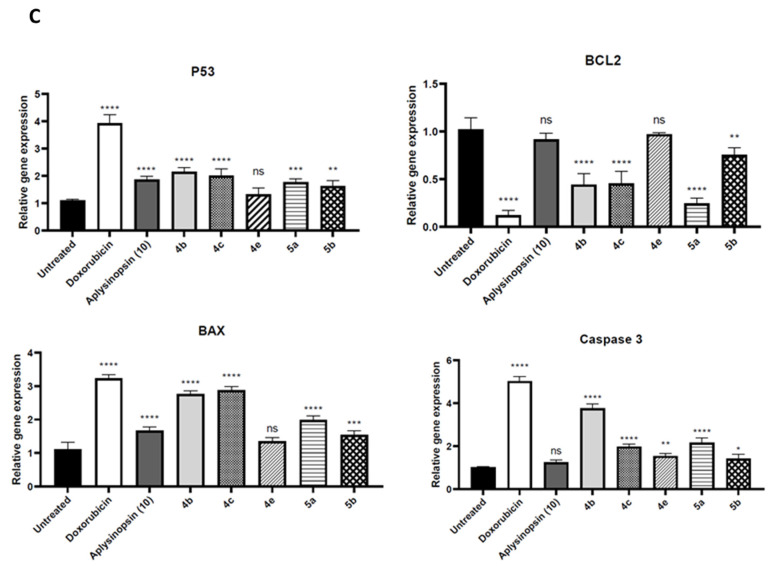
(**A**). Dot plots of apoptotic cells populations using PI /Annexin V-FITC staining for FACS analysis. (**B**). Percentages of early, late apoptosis and necrosis induced by analogs **4b**, **4c**, **4e**, **5a**, **5b,** and aplysinopsin (**10**) in PC3 cells. (**C**). Relative gene expression of apoptosis related genes (P53, Caspase-3, Bcl2 and Bax) induced by aplysinopsin and its analogs. Data are represented as mean ± SD of three individual experiments. Statistical analysis was done by applying either one-way or two-way ANOVA followed by with Dunnett’s multiple comparison test; * *p* < 0.05, ** *p* < 0.01, *** *p* < 0.001, **** *p* < 0.0001 compared to the control.

In order to understand the mechanism by which the synthesized analogs induced their cytotoxic action, we incubated the active compounds with PC-3 cells for 24 h. Cells were stained with both PI and Annexin V-FITC, then the flow cytometric analysis was conducted. Both dyes were used to differentiate between early and late apoptosis. Early apoptotic cells were (PI^−^/Annexin V-FITC^+^), late apoptotic cells were (PI^+^/Annexin V-FITC^+^), necrotic cells were (PI^+^/Annexin V-FITC^-^) while healthy cells remained unstained (PI^−^/Annexin V-FITC^−^). Figure 1A shows the dot blots of the most active analogs **4b**, **4c**, **4e**, **5a**, **5b,** and aplysinopsin (**10**). 

As listed in Table S3, all analogs **4b**, **4c**, **4e**, **5a**, **5b,** and aplysinopsin (**10**) exhibited their cytotoxic activity mostly via apoptosis. This was evident by the low levels of induced necrosis compared with the significant increase in the induced levels of early and late apoptosis of all analogs as illustrated in Figure 1B. The analog **4c** induced the highest levels of cell death (43.07%). Moreover, **4c** displayed the highest levels of both early apoptosis (14.17%), late apoptosis (23.24%) and very low levels of necrosis (5.66%) as demonstrated in Figure 1B. This shows that **4c** analog’s cytotoxic activity is attributed to the controlled process of apoptosis and not to necrosis. Therefore, **4c** is considered to be superior to the other analogs due to its high apoptotic activity with low necrotic effects. On the other hand, the analog **5b** showed the lowest levels of early apoptosis (9.68%), late apoptosis (9.53%) and necrosis (7.22%). These data indicate that aplysinopsin analogs exhibit their cytotoxic effects mainly via apoptosis.

For further elucidation of the molecular mechanism involved in aplysinopsin analogs apoptotic activity, we assessed the expression levels of p53, Bcl2, Bak and Caspase 3. We analyzed the expression levels of these genes induced by the five aplysinopsin analogs **4b**, **4c**, **4e**, **5a**, and **5b** that showed the highest apoptotic activity and their parent compound aplysinopsin (**10**). This was conducted in comparison to a negative control and the positive control, doxorubicin. As illustrated in Figure 1C, all analogs showed a significant increase in the expression of all the genes except compound **4e**. In addition, the synthesized analogs **4b, 4c, 5a** and **5b** only showed a significant decrease in the expression levels of the anti-apoptotic gene Bcl2 but not aplysinopsin (**10**). The inhibition of Bcl2 expression resulted in inducing Bak and Caspase 3 expression by these analogs. As expected, the inability of aplysinopsin (**10**) to suppress Bcl2 expression hindered the increase in Caspase 3 expression. These data explain that suppressing Bcl2 expression is the main the mechanism by which aplysinopsin analogs induced their cytotoxicity against prostate cancer. 

### 2.3. Aplysinopsin Analogs Promoted Cell Cycle Arrest at S-Phase in Prostate Cancer

**Figure 2:**
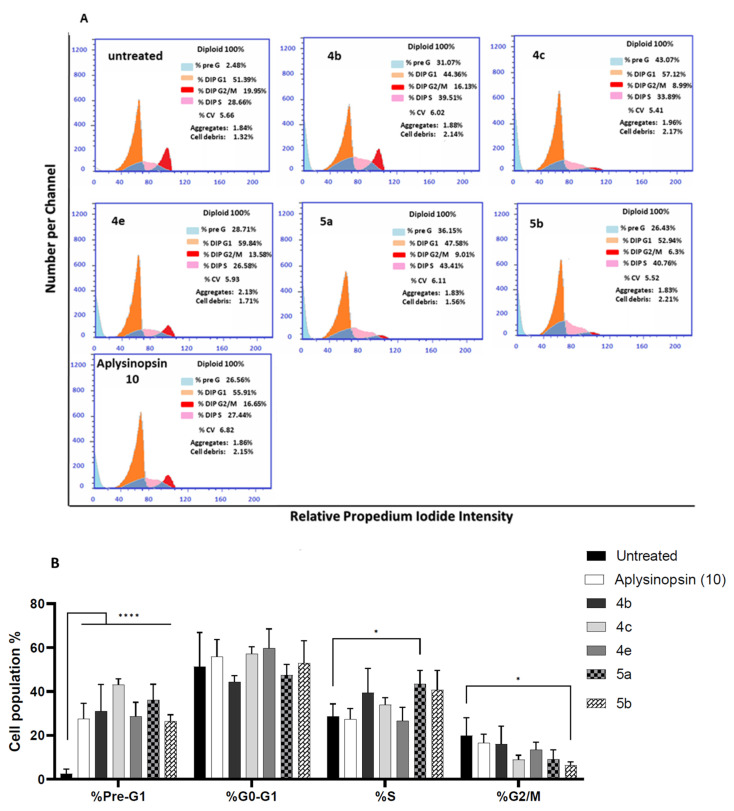
(**A**). Histograms of cell cycle phase distribution using PI staining for FACS analysis. (**B**). Percentages of cells accumulation in Pre-G1, G0-G1, S and G2/M cell cycle phases induced by analogs **4b**, **4c**, **4e**, **5a**, **5b,** and aplysinopsin (**10**). Data are represented as the mean ± SD of three independent experiments. Statistical analysis was conducted using two-way ANOVA followed by Dunnett’s multiple comparison test; * *p* < 0.05, **** *p* < 0.0001 compared to the control.

Since the cytotoxicity of the synthesized analogs against prostate cancer cells was induced by elevated apoptosis levels, we examined whether these analogs further induced cell cycle arrest. To analyze cell cycle distribution, PC3 cells were treated with aplysinopsin analogs for 24 h, stained with PI then followed by flow cytometric analysis. Figure 2A demonstrates the histogram of each analog, while the percentages of cells in each phase of the cell cycle are further enlisted in Supplementary Table S4. 

The cells distribution at the different phases of the cell cycle is displayed in Figure 2B. It shows that the analog **5a** significantly induced cell cycle arrest mainly at S-phase (43.41%) compared with untreated cells (28.66%). On the other hand, the analog **5b** significantly decreased the cell population at G2-phase (6.3%) compared with untreated cells (19.95%). In addition, the percentages of cell population at pre-G1 phase significantly increased after PC3 cells were treated with all the five active analogs and their parent compound aplysinopsin (**10**) compared with untreated cells (2.48%). Hence, the cytotoxicity of some of aplysinopsin analogs against prostate cancer can be attributed to another mechanism which is the promotion of cell cycle arrest at S-phase. 

### 2.4. Bioavailability of Aplysinopsin Analogs According to Lipinski’s Rule of Five 

Next, we examined the drug-likeness of aplysinopsin (**10**) and its analogs **4b**, **4c**, **4e**, **5a**, and **5b** according to Lipinski’s rule of five using the free tool SwissADME [27] (Table 1). 

According to Lipinski’s rule of five, all studied compounds could have high chances of oral bioavailability due to their compatibility with Lipinski’s rule of five as follows: the molecular weights (<500), the octanol–water partition coefficient, lipopholicity, LogP values (<4.15), H-bond donor (HBD ≤ 5), H-bond acceptor (HBA ≤ 10), and atomic molar reactivity (MR) (40 to 130) (Table 1). Compounds **4c**, **4e**, and **5b** showed one remark; their molar reactivity slightly exceed the optimal range of MR, where the values were 132.57, 142.18, and 134.93 respectively.

### 2.5. Molecular Docking of Aplysinopsin (10) and Its Analogs ***4b***, ***4c***, ***4e***, ***5a***, and ***5b*** towrds Anti-Apoptotic Protein BCL2

**Figure 3:**
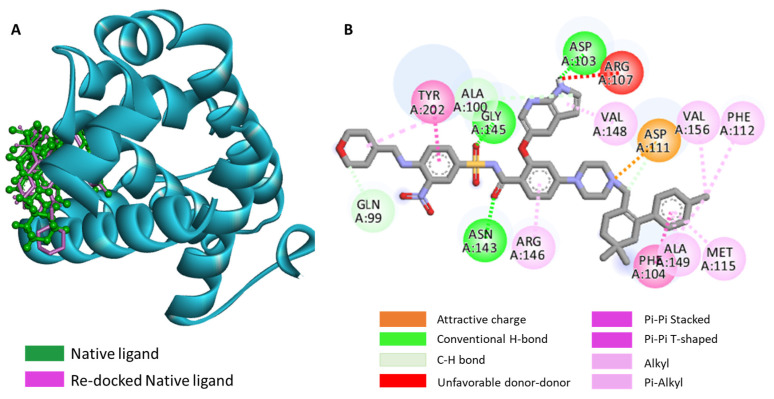
(**A**). The 3D binding mode of the re-docked ligand (purple, sphere and stick) within the binding pocket of BCL2 (PDB ID: 6O0K). It is shown to be superimposed on the same position as the original ligand (green, ball and stick), with the same orientation. (**B**). The 2D binding mode of the re-docked ligand LBM within the binding pocket of BCL2 (PDB: 6O0K).

The induction of apoptosis by the five most active analogs led to investigation of interaction with the target anti-apoptotic protein BCL2 using molecular docking. Accordingly, BCL2 crystal structure was obtained from the protein data bank. Its native ligand fundamental poses were employed as references for validating the simulation process and determining the grid box dimensions and positioning. It was noticed that the native ligand exhibit numerous hydrogen bonds interaction with the target including ASP103, GLY145, ASN143, and GLN99, besides Pi-Pi and Pi-alkyl interactions (Figure 3). 

### 2.6. Assessment of the Binding Mode of the Studied compounds with BCL2 (PDB ID: 6O0K)

**Figure 4:**
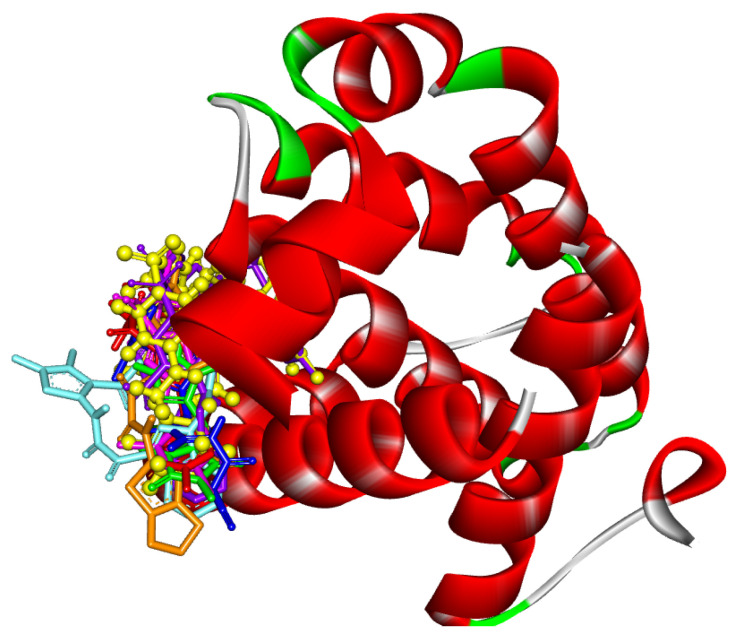
The 3D orientation of co-crystalline ligand of BCL2 (yellow, ball, and stick), the re-docked ligand (purple), compound **4b** (blue sky); compound **4c** (orang); compound **4e** (red); compound **5a** (dark blue); compound **5b** (pink); compound **10** (green) inside the binding pocket of BCL2 (PDB: 6O0K).

The docking result of all studied compounds indicated that all of them were superimposable to LBM. In addition, they showed good binding mode with the active pocket through the formation of multiple interactions with the key amino acids of BCL2 in a similar manner as LBM (Table S6, Figure 4). Moreover, they revealed good binding energy towards the binding site of BCL2 (PDB ID: 6O0K) with values ranging from −9.2 to −8.3 kcal/mol (Table S6). The most promising compounds showing the lowest binding energy and establishing good binding interactions with both the binding pocket and the allosteric site of the BCL2 followed the order: **4e** (−9.2Kcal/mol) > **5a** (−8.9 Kcal/mol) > **4c** and aplysinopsin (**10**) (−8.7 Kcal/mol) > **5b** (−8.5 Kcal/mol) > **4b** (−8.3 Kcal/mol).

**Figure 5:**
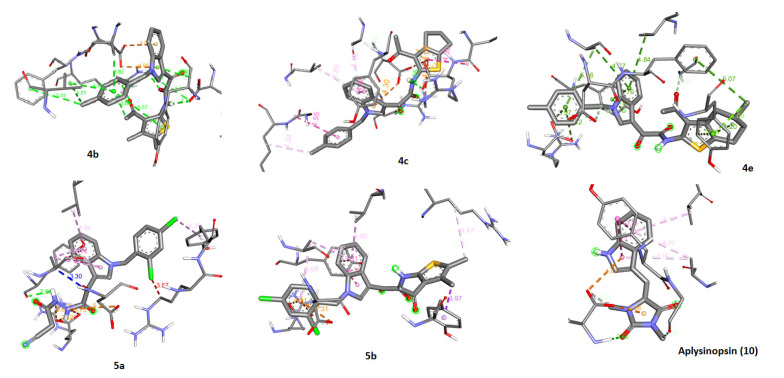
The 3D interactions of aplysinopsin (**10**) and its analogs **4b**, **4c**, **4e**, **5a**, and **5b** inside the binding pocket of BCl2 (PDB: 6O0K).

**Figure 6:**
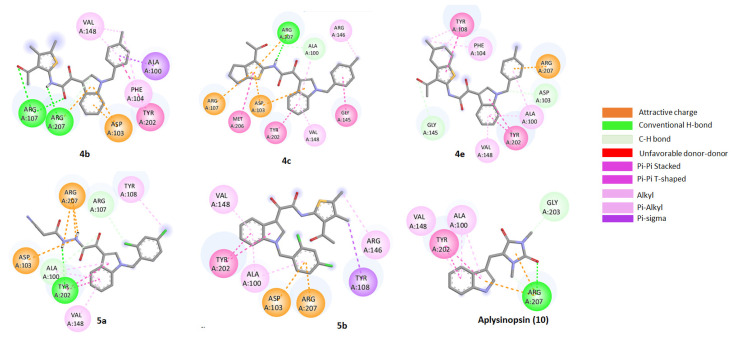
The 2D interaction of aplysinopsin (**10**) and its analogs **4b**, **4c**, **4e**, **5a**, and **5b** within the binding pocket of BCl2 (PDB: 6O0K) illustrating the formed hydrogen bonds, attractive charge, pi-pi bond, and pi-alkyl.

We propose that the formed hydrogen bonds, attractive charge, pi-pi bond, and pi-alkyl modulate these analogs mode of action. Generally, the thiophene, indole and cyclohexane moieties are susceptible to play the role of the aromatic heterocyclic moiety in the native ligands (LBM) (Figure 5 and Figure 6).

## 3. Discussion

Aplysinopsins are marine indole derivatives that have demonstrated different biological activities such as antileishmanial [28], antimicrobial [19,20], antiplasmodial [29] and antifilarial [30]. They also induced neurotransmitters modulation such as anti-cholinesterase activity [23], monoamine oxidase inhibition [22,31] and serotonin receptor affinity modulation [32]. This modulation controls several diseases such as depression, schizophrenia, anxiety, Parkinson’s and Alzheimer’s disease [32]. Moreover, aplysinopsins exhibited antitumor activity [24,33] against breast cancer [25], melanoma [24], ovarian cancer [24] and Leukemia [26].

One of the main mechanisms of anticancer agents is apoptotic pathway activation [34]. During apoptosis, cells undergo a controlled process of cell death which is characterized by protein cleavage and DNA fragmentation [35]. As expected, our data revealed that the cytotoxic activity of aplysinopsin analogs against prostate cancer was induced by apoptosis. These data are aligned with another study that showed apoptosis as a mechanism controlling aplysinopsins cytotoxic activity against chronic myeloid leukemia [26]. Moreover, we showed that p53, Bcl2, Bax and Caspase3 were involved in aplysinopsins analogs apoptotic activity. P53 is known to play a major role in mediating tumor growth suppression and its loss contribute to tumor induction and growth [36,37]. We found that its expression was upregulated in PC-3 cells treated with aplysinopsin analogs which leads to the activation of p53 and apoptotic cell death [38]. BCL2 is another key player that modulates apoptosis. It is considered an anti-apoptotic protein which is highly expressed in most of tumors [35,36,39,40]. Therefore, BCL2 was down-regulated in prostate cancer cells treated with the most active five aplysinopsin analogs that we developed. Along with these results, other recently discovered indole derivatives (indole-1,2,4-triazoles) also induced their anti-proliferative activity through the gradual decrease of Bcl2 levels [41]. In addition, we analyzed the expression levels of pro-apoptotic genes such as Bax and Caspase3. Bax causes the assembly of a channel in the mitochondrial outer membrane leading to its permeabilization and the release of cytochrome c which triggers apoptosis [42]. We found that Bax was upregulated in PC-3 cells treatment with aplysinopsin analogs due to its activation after Bcl2 inhibition. The activated Bax induced caspase3 leading to its increased expression. Other indole derived alkaloids such as evodiamine and violacein were also reported to increase in Bax expression leading to apoptosis in human gastric adenocarcinoma cells [43] and Head and neck carcinoma (HNC), respectively [44]. Due to the previous changes in p53, Bcl2 and Bax levels, the downstream effector Caspase 3 expression was elevated after treating prostate cancer cells with our synthesized aplysinopsin analogs especially **4c**. These results altogether show apoptosis as a mechanistic pathway causing aplysinopsin analogs anti-proliferative activity.

Furthermore, we explored the active analogs potential to cause cell cycle arrest as another mechanism for their antitumor activity. Cell cycle is composed of well-coordinated phases that finally leads to cellular division. A dysregulated cell cycle is associated with uncontrolled cell division leading to tumor growth [45]. Hence, cell cycle arrest deprives tumor cells from their ability to proceed to cell proliferation which in turn has beneficial outcomes in cancer treatment [46]. We investigated the distribution of cells at the different phases of cell cycle after PC3 cells treatment with the active aplysinopsin analogs. **5a** Analog demonstrated cell cycle arrest at S-phase. Cell cycle arrest at different phases was evident in another synthesized indole-1,2,4-triazole derivative bearing the 3,4,5-trimethoxyphenyl moiety. This derivative caused cell cycle arrest at G2/M phases via decreasing cyclin B1 expression [41]. Moreover, evodiamine significantly induced G2/M phase cell cycle arrest, thus causing the inhibition of human gastric carcinoma cells proliferation [43]. Another key player in cell cycle arrest is p53 upregulation, which was evidently upregulated by our synthesized aplysinopsin derivatives. This upregulation of p53 expression causes cell cycle arrest via p21 activation [36,45]. Therfore, we propose that the increased expression of p53 which we described earlier resulted in the induction of cell cycle arrest by the active analog **5a**. Taken all together, the five most active analogs **4b**, **4c**, **4e**, **5a**, **5b,** and their parent compound, aplysinopsin (**10**) activated both apoptosis and cell cycle arrest to exert their antitumor activity. This dual mechanism of action was also reported in a recently discovered indole derivative against osteosarcoma called NSC743420 [47]. Therefore, our synthesized analogues are highly promising candidates for prostate cancer treatment. 

## 4. Materials and Methods

### 4.1. Chemistry

#### 4.1.1. General Information

All reagents and solvents were of commercial grade. Melting points were determined on the digital melting point apparatus (Electro thermal 9100, Electro thermal Engineering Ltd., serial No. 8694, Rochford, UK) and are uncorrected. Elemental analyses were performed on a Flash Smart™ Elemental Analyzer (Thermo Scientific, Courtaboeuf, France) and were found within ± 0.4% of the theoretical values. ^1^H and ^13^C NMR spectra were measured with a Bruker Avance spectrometer (Bruker, Germany) at 400 and 101 MHz, respectively, using TMS as the internal standard. Hydrogen coupling patterns are described as (s) singlet, (d) doublet, (t) triplet, (q) quartet, and (m) multiplet. The chemical shifts were defined as parts per million (ppm) relative to the solvent peak. The reaction progress was checked by pre-coated TLC Silica gel 0.2 nm F254 nm [Fluka], visualized under UV lamp 254 and 365 nm. Cyanoacetic acide hydrazide [48], *N*-benzyl indoles [49]; methyl creatinine [32]; indole-3-aldehyde [50] were prepared as reported.

#### 4.1.2. General Procedure for the Preparation of Aplysinopsin Analogs (EE) **3c**–**g**, **4b**–**g** and **5a**–**g**

A solution of oxalyl chloride (0.44 ml, 5.1 mmol) in dry ethyl ether (25 ml) was treated with a solution of indoles (4.14 mmol) in dry ethyl ether (5 ml) dropwise under cooling. The resulting yellow slurry was refluxed for 2 h. After removing the ether under vacuum, the remains were dissolved in dry tetrahydrofuran (20 ml), and then cooled to 0 °C. A solution of amines (9.73 mmol) in dry tetrahydrofuran (20 ml) was added slowly to the THF solutions under stirring. After complete addition, 1 ml of triethylamine was added to the reaction mixture and left to stir overnight. The formed precipitate was filtered off, washed several times with water, dried, and recrystallized from acetone.

*N*-(3-Acetyl-4,5-dimethylthiophen-2-yl)-2-(1-(4-methylbenzyl)-1*H*-indol-3-yl)-2-oxo-acetamide (4b). Yield (0.28g, 47%); mp 201-3 °C; ^1^H-NMR (400 MHz, CDCl_3_) δ 13.46 (s, 1H), 9.11 (s, 1H), 8.56 (d, *J* = 7.8 Hz, 1H), 7.46 – 7.05 (m, 7H), 5.37 (s, 2H), 2.63 (s, 3H), 2.43 – 2.27 (m, 6H), 1.58 (s, 3H); ^13^C-NMR (101 MHz, CDCl_3_) δ 145.02, 141.09, 138.12, 136.55, 132.33, 129.72, 127.93, 126.99, 125.21, 124.42, 124.12, 123.64, 123.02, 110.60, 51.14, 31.40, 21.08, 15.27, 12.78; HRMS-(ESI): *m*/*z* [M+Na]^+^ calcd for C_26_H_24_N_2_O_3_SNa (467.14); Anal calcd for: C_26_H_24_N_2_O_3_S (444.55): C, 70.25; H, 5.44; N, 6.30; S, 7.21; found: C, 70.12; H, 5.22; N, 6.21; S, 7.01.

*N*-(3-Acetyl-5,6-dihydro-4*H*-cyclopenta[*b*]thiophen-2-yl)-2-(1-(4-methylbenzyl)-1*H*-indol-3-yl)-2-oxo-acetamide (4c). Yield (0.37g, 60%); mp 233-5 °C; ^1^H-NMR (400 MHz, CDCl_3_) δ 13.40 (s, 1H), 9.11 (s, 1H), 8.56 (d, *J* = 7.8 Hz, 1H), 7.48 – 7.02 (m, 9H), 5.38 (s, 2H), 3.11 – 2.90 (m, 2H), 2.54 (d, *J* = 4.9 Hz, 2H), 2.34 (s, 3H), 1.59 (s, 3H); ^13^C-NMR (101 MHz, CDCl_3_) δ 195.54, 177.38, 160.21, 150.40, 141.11, 141.00, 138.11, 136.56, 134.18, 132.34, 129.72, 127.93, 126.98, 124.13, 123.64, 123.02, 119.02, 112.44, 110.62, 51.13, 31.36, 29.98, 28.73, 28.12, 21.08; Anal calcd for C_27_H_24_N_2_O_3_S (456.56): C, 71.03; H, 5.30; N, 6.14; S, 7.02; found: C, 71.00; H, 5.22; N, 6.24; S, 6.98.

*N*-(3-Acetyl-5-methyl-4,5,6,7-tetrahydrobenzo[*b*]thiophen-2-yl)-2-(1-(4-methylbenzyl)-1*H*-indol-3-yl)-2-oxo-acetamide (4e). Yield (0.40g, 61%); mp 208-10 °C; ^1^H-NMR (400 MHz, CDCl_3_) δ 13.55 (s, 1H), 9.11 (d, *J* = 9.5 Hz, 1H), 8.56 (d, *J* = 7.9 Hz, 1H), 7.42 – 7.05 (m, 7H), 5.37 (s, 2H), 2.96 (dt, *J* = 34.9, 17.4 Hz, 1H), 2.83 – 2.67 (m, 2H), 2.58 (s, 3H), 2.46 – 2.32 (m, 1H), 2.35 (s, 3H), 2.05 – 1.82 (m, 1H), 1.63 – 1.44 (m, 2H), 1.16 (s, 3H); ^13^C-NMR (101 MHz, CDCl_3_) δ 196.33, 177.53, 160.33, 146.34, 141.09, 138.11, 136.55, 132.34, 130.41, 129.72, 128.23, 127.93, 127.00, 124.11, 123.63, 123.10, 123.02, 112.46, 110.60, 51.13, 35.96, 31.58, 30.73, 29.30, 24.44, 21.76, 21.08; HRMS-(ESI): *m*/*z* [M+Na]^+^ calcd for C_29_H_28_N_2_O_3_SNa (507.16); Anal calcd for C_29_H_28_N_2_O_3_S (484.61): C, 71.88; H, 5.82; N, 5.78; S, 6.62; found: C, 71.89; H, 5.77; N, 5.81; S, 6.59.

N′-(2-cyanoacetyl)-2-(1-(2,4-dichlorobenzyl)-1*H*-indol-3-yl)-2-oxoacetohydrazide (5a). Yield (0.36g, 77%); mp 256-8 °C; ^1^H-NMR (400 MHz, DMSO-*d_6_*) δ 10.81 (s, 1H), 10.41 (s, 1H), 8.89 (s, 1H), 8.21 (s, 1H), 7.66 (d, *J* = 8.8 Hz, 1H), 7.35 (d, *J* = 8.7 Hz, 1H), 7.18 (dd, *J* = 20.3, 8.1 Hz, 4H), 5.56 (s, 2H), 3.83 (s, 2H); ^13^C-NMR (101 MHz, CDCl_3_) δ 194.03, 180.86, 162.03, 161.47, 141.95, 137.40, 135.07, 133.25, 129.46, 129.37, 129.31, 128.14, 127.86, 127.56, 127.47, 123.90, 120.66, 115.55, 113.56, 111.24, 69.79, 50.04, 45.84; Anal calcd for C_20_H_14_Cl_2_N_4_O_3_ (429.26): C, 55.96; H, 3.29; Cl, 16.52; N, 13.05; found: C, 55.88; H, 3.21; N, 12.99.

*N*-(3-Acetyl-4,5-dimethylthiophen-2-yl)-2-(1-(2,4-dichlorobenzyl)-1*H*-indol-3-yl)-2-oxo-acetamide (5b). Yield (0.39g, 72%); 227-9 °C; ^1^H-NMR (400 MHz, CDCl_3_) δ 13.47 (s, 1H), 9.09 (s, 1H), 8.58 (d, *J* = 7.9 Hz, 1H), 7.52 – 7.09 (m, 5H), 6.73 (d, *J* = 8.3 Hz, 1H), 5.49 (s, 2H), 2.62 (s, 3H), 2.37 (d, *J* = 10.0 Hz, 6H); ^13^C NMR (101 MHz, CDCl_3_) δ 196.72, 177.73, 160.07, 144.92, 141.04, 136.35, 134.83, 133.36, 131.69, 129.73, 129.06, 128.49, 127.81, 127.70, 125.36, 124.46, 123.89, 123.11, 112.87, 110.26, 45.86, 31.39, 8.63; HRMS-(ESI): *m*/*z* [M+Na]^+^ calcd for C_25_H_20_Cl_2_N_2_O_3_SNa (521.04); Anal calcd for C_25_H_20_Cl_2_N_2_O_3_S (499.41): C, 60.13; H, 4.04; N, 5.61; S, 6.42; found: C, 60.01; H, 4.00; N, 5.54; S, 6.32.

#### 4.1.3. Preparation of (Z)-5-((1H-indol-3-yl)methylene)-1,3-dimethylimidazolidine-2,4-dione (Aplysinopsin, **10**) [26,51]

A mixture of 1,3-dimethyl creatinine (2.8 g, 22.7 mmol) and indole-3-aldehyde (22.7 mmol) was heated under reflux in piperidine (30 mL) for 4 h. After cooling, the reaction mixture was poured into water (200 mL) and then stirred for 30 min. The precipitate was filtered, washed several times with water, air dried and crystallized from methanol. Yield 80%, mp 236–8 °C (reported mp 236 °C); ^1^H NMR (400 MHz, CDCl_3_) δ: 8.87 (d, *J* = 2.7 Hz, 1H), 8.54 (s, 1H), 7.85–7.66 (m, 1H), 7.45 (dd, *J* = 6.7, 1.4 Hz, 1H), 7.33–7.18 (m, 3H), 6.42 (s, 1H), 3.34 (s, 3H), 3.23 (s, 3H).

### 4.2. Biological Assays

#### Chemicals

Doxorubicin, DMEM, DMEM-F12, penicillin/streptomycin, trypsin solution and fetal bovine serum were purchased from Lonza, Spain. 3-(4,5-dimethylthiazolyl-2)-2,5-diphenyltetra-zolium bromide (MTT) was obtained from Sigma-Aldrich, St. Louis, MO, USA. Triton X-100 was from Pierce Biotechnology Inc., Rockford, IL, USA.

### 4.3. Cell Lines and Cell Cultures

HCT-116 (colon), HepG2 (liver), MCF-7 (breast), PC3 (prostate) and A549 (lung) cancer cell lines were kindly supplied by Professor Stig Linder, Oncology and Pathology department, Karolinska Institute, Stockholm, Sweden, and were formerly obtained from the American Type Culture Collection (ATCC). Cancer cell lines were grown in Dulbecco’s modified Eagle’s medium (DMEM) supplemented with 10% fetal bovine serum (FBS). Cells were cultured in DMEM media supplied with 10 % fetal bovine serum, 100 U/mL penicillin/streptomycin. The cells were maintained at 37 °C in 5 % CO_2_.

### 4.4. Cell Proliferation and Viability

The cytotoxic activity against the different cancer cell lines was determined according to the method of Thabrew et al. [52], with slight modifications. The cells were seeded into a 96-well plate at a concentration of 20,000 cells/well for HCT-116 and PC3 cell lines and 10000 cells/well for HepG2, A549 and MCF-7 cell lines. After 24 h, the media was aspirated and replaced with serum-free media containing the tested compounds (100 μM). The cells were treated for 48 h in triplicates. For the positive and negative controls, doxorubicin (100 μM) and dimethyl sulfoxide (DMSO) (0.5 %) were used, respectively. Cell viability in response to treatments was calculated using the MTT [3-(4, 5-dimethylthiazol-2-yl)-2,5-diphenyltetrazolium bromide] assay [53]. The percentage of cytotoxicity was calculated using the following equation:

% Cytotoxicity = [1-(AV_x_/AV_NC_)] × 100 (1)

where AVx denotes the average absorbance of the sample well and AV_NC_ denotes the average absorbance of the negative control well measured at 595 nm with reference at 690 nm.

### 4.5. Determination of IC_50_ Values

Active hits possessing cytotoxicity ≥ 60 % on different cancer cell lines were selected for dose-response studies at different concentrations. The final tested concentrations were 100, 50, 25, 12.5, and 6.25 μM in triplicates. The IC_50_ values were calculated using the concentration-response curve fit to the non-linear regression model using Graph Pad Prism® v6.0 (GraphPad Software Inc., San Diego, CA, USA).

### 4.6. Gene Expression Analysis by Quantitative Real-Time PCR Method (qPCR)

The levels of gene expression of P53, Bax, Caspase-3 and Bcl-2 were determined using the Step One Plus quantitative RT-PCR system (Applied Biosystems, 180 Oyster Point Blvd, South San Francisco, CA, USA) with respect to SYBR Green (Thermo-Scientific, Waltham, MA, USA) method [54].

### 4.7. RNA Isolation

The assay was done following the procedure of Oliveira et al. [54]. In brief, cells were seeded on a six-well plate at a concentration of 3 × 10^5^ cells/well. After a 24-h incubation, the IC_50_ dose of active compounds were applied to each well for 48 h. Total RNA was isolated according to the Trizol reagent protocol. The cells were collected and centrifuged at 2500 rpm for 15 min at 4 °C. The total RNA isolation kit (RNeasy extraction kit- QIAGEN) was used to isolate RNA while TranScriba First Strand cDNA Synthesis kit (BIORAD) was used to obtain cDNA according to the manufacturer’s instructions.

### 4.8. Reverse Transcription (RT) Reaction

The RT-PCR analysis was performed using specific primers for each gene. To identify genes expression, the results of the selected genes expression were normalized to the β-actin housekeeping gene (Table 2). The RT reaction was started by incubation at 50 °C for 45min for cDNA synthesis and followed by real-time PCR amplification cycles (95 °C for 10 sec and 60 °C for 60 sec, 40 cycles) in a Rotor-Gene 3000 (Corbett Robotics, Australia). Negative control was also used in each run to access the specificity of primers and possible contamination [55].

### 4.9. Cell Cycle Analysis by Flow Cytometry Assay

Annexin-V-FITC Apoptosis Detection Kit (BIPEC, USA) was used to determine apoptotic cells. PI staining was used to reveal cell cycle stage. Briefly, cells treated with each analog were resuspended with 400 μl binding buffer, labeled with annexin-V-FITC for 15min and PI for another 5min at 4 °C. The fluorescence of the stained cells was analyzed using flow cytometry by by FACSCalibur (BD Biosciences, Mountain View, CA). The data were analyzed using the CellQuest software (BD Biosciences).

### 4.10. In Silico Prediction for the Drug-Likeness

Drug-likeness of the aplysinopsin and its analogs were calculated and interpreted based on Lipinski’s rule of five using a free tool SwissADME [27].

### 4.11. Molecular Docking 

Molecular docking studies of active aplysinopsin analogs together with anti-apoptotic protein BCL2 was performed using PyRx tools Autodock Vina (version 1.1.2) [56]. The crystal structure of BCL2 complexed with the inhibitor (LBM), 4-{4-[(4′-chloro-5,5-dimethyl[3,4,5,6-tetrahydro[1,1′-biphenyl]]-2-yl)methyl] piperazin-1-yl}-*N*-[(3- nitro-4-{[(oxan-4-yl)methyl]amino}phenyl)sulfonyl]-2-[(1*H*-pyrrolo[2,3-*b*]pyridin-5-yl) oxy] benzamide was retrieved from the protein data bank at https://www.rcsb.org/structure/6o0k (access on 26 October 2022) using 6O0K code. 

The native ligand and the water molecules were removed from the protein using VEGA ZZ 2.3.2 tool followed by adding polar hydrogen and Kollman charges and then converted to PDBQT format by Autodock Vina tools. All the molecules were constructed with the ChemDraw ultra 10.0 and saved as mol file, then protonated, minimized and converted to pdb file by Open Babel software. The created pdb file was submitted to Autodock Vina tools to set a number of torsion and for pdbqt file construction. 

AutoGrid was used with a grid box to create the grid map. The grid box coordinates were X = -15.3231 Å, Y = 2.21374 Å, Z = -9.59347 Å to cover the BCL2 binding pocket. The number of docked poses generated for each compound at the active pocket of BCL2 is 10 and subsequently was ranked according to the binding energy. The pose of lowest binding energy and 0 Å root-mean-square deviation (RMSD) was considered to be the fittest and complexed with receptor for analysis. The molecular interactions and binding modes of the top poses were visually examined using BIOVIA Discovery Studio 2021.

### 4.12. Statistical Analysis

Comparisons between multiple treatments were made by either one-way or two-way ANOVA, followed by Dunnett’s multiple comparison test; * *p* < 0.05, ** *p* < 0.01, *** *p* < 0.001, **** *p* < 0.0001 were considered to be a significant difference. GraphPad Prism 8 (GraphPad Software, San Diego, CA, USA) was used for statistical analysis and plotting the graphs.

## 5. Conclusions

Several synthesized aplysinopsin analogs were screened against different cancer cell lines, namely human breast cancer (MCF-7), human colon cancer (HCT-116), human liver cancer (HepG2), human non-small cell lung cancer (A549) and human prostate cancer (PC3). Compounds **4b**, **4c**, **4e**, **5a** and **5b** were the most active analogs with potent selective cytotoxicity against the PC3 cell line. The induction of apoptosis and cell cycle arrest, which are responsible for cytotoxic activity, was studied for the most active aplysinopsin analogs. The suppression of BCL2 by these analogs resulted in their apoptotic activity, along with Bax, Caspase 3 and p53 induction. This dual mechanism of action via apoptosis and cell cycle arrest induction is responsible for aplysinopsin analogs antitumor activity. Hence, our newly synthesized analogs are highly promising candidates for further preclinical studies against prostate cancer. All studied compounds could have high chances of oral bioavailability due to their compatibility with Lipinski’s rule of five.

## Figures and Tables

**Table 1:** Drug-likeness of aplysinopsin (**10**) and its analogs were calculated and interpreted based on Lipinski’s rule of five.

Lipinski’s rule of five	aplysinopsin (**10**)	**4b**	**4c**	**4e**	**5a**	**5b**
MW (g/mol)	255.27	444.55	456.56	484.61	429.26	499.41
Log *P*_o/w_	0.79	2.57	2.77	3.17	1.71	3.31
HBA	1	3	3	3	4	3
HBD	2	1	1	1	2	1
MR	79.91	129.88	132.57	142.18	108.07	134.93
No. Lipinski violation	0	0	0	0	0	0

MW: Molecular weight ≤ 500; Log P: lipophilicity < 4.15; HBA: Hydrogen bond acceptor ≤ 10; HBD: Hydrogen bond donor ≤ 5; MR: Molar reactivity (40 ≤ MR ≤ 130).

**Table 2:** Primer Sequence of different genes.

Gene	Forward Strand	Reverse Strand
p53	5′-CCCCTCCTGGCCCCTGTCATCTTC-3′	5′-GCAGCGCCTCACAACCTCCGTCAT-3′
Bcl2	5′-CCTGTG GAT GAC TGA GTA CC-3′	5′-GAGACA GCC AGG AGA AAT CA-3′
Bax	5′-GTTTCA TCC AGG ATC GAG CAG-3′	5′-CATCTT CTT CCA GAT GGT GA-3′
Caspase-3	5′-GGAAGCGAATCAATGGACTCTGG-3′	5′-GCATCGACATCTGTACCAGACC-3′
β -actin	5′-GTGACATCCACACCCAGAGG-3′	5′-ACAGGATGTCAAAACTGCCC-3′

## Data Availability

Not applicable.

## Supplementary Materials

The following supporting information can be downloaded at: https://www.mdpi.com/article/10.3390/molecules28010109/s1. ^1^H, ^13^C NMR and mass spectral data of aplysinopsin analogs. Table S1. The cytotoxicity of aplysinopsin (10) and its analogs 3c-g, 4b-g and 5a-g against different cell lines. Table S2. The IC_50_ of aplysinopsin (10) and its analogs 3c-g, 4b-g and 5a-g against PC3 cell line. Table S3. Apoptosis phase after treatment of PC3 cells with aplysinopsin analogs for 24h. Table S4. Gene expression analysis (expressed as fold Change B-actin) of active compounds on Prostate cancer cell line. Table S5. Cell cycle phase distribution after treatment of PC3 cells with aplysinopsin analogs for 24h. Table S6. The molecular docking result of aplysinopsin (10), and its analogs 4b, 4c, 4e, 5a, and 5b.
