# Supplementary material for: Synthetic Analogs of Marine Alkaloid Aplysinopsin Suppress Anti-Apoptotic Protein BCL2 in Prostate Cancer
Source: Molecules. 2022 Dec 23;28(1):109. doi: 10.3390/molecules28010109 (PMC9821956; doi:10.3390/molecules28010109)
Supplement: Supplementary file 1 [file molecules-28-00109-s001.zip › NMR MS supporting information.pdf]

L2CM Salma EE-65
Salma EE-65

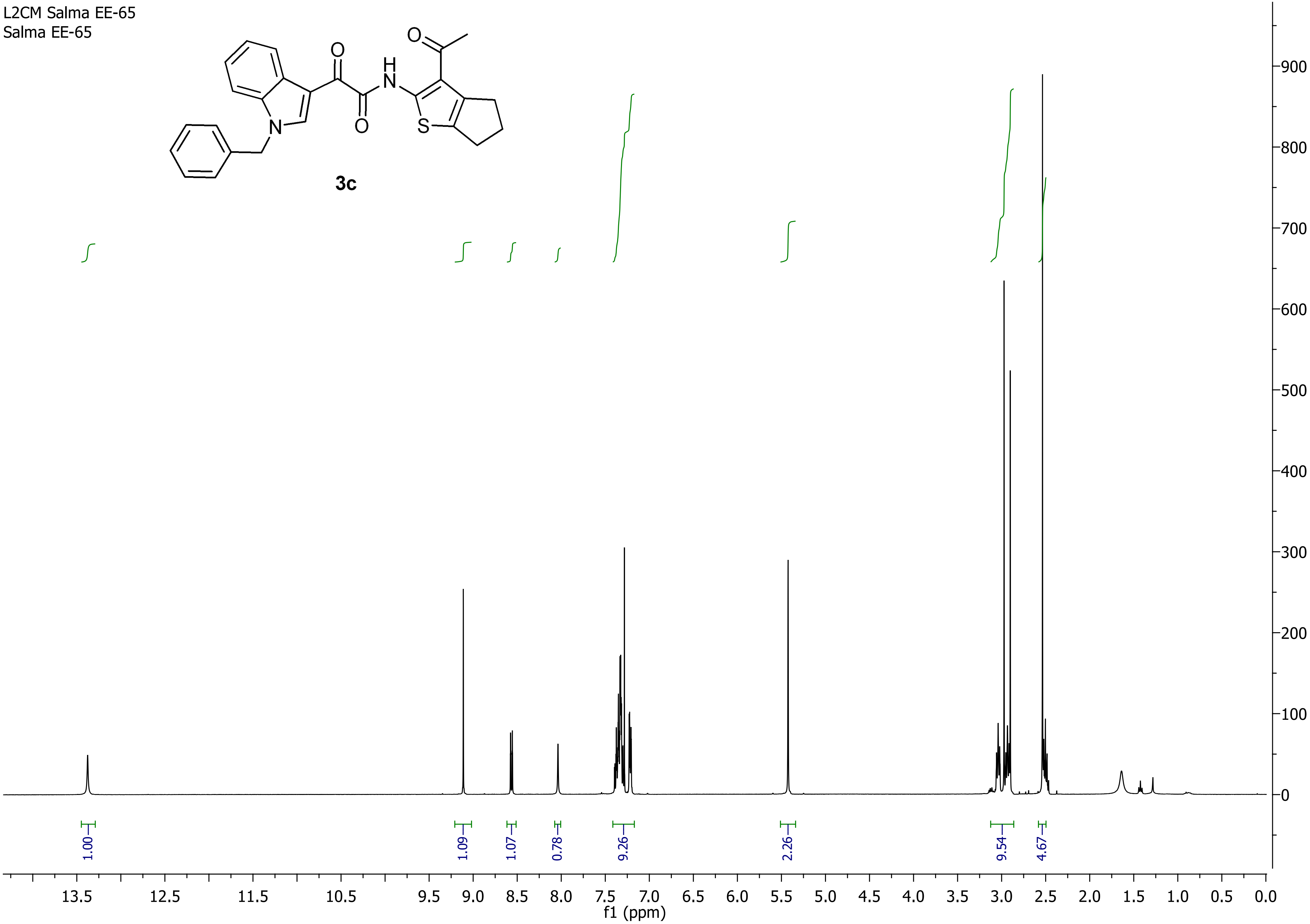

L2CM Salma EE-65 13C CPD

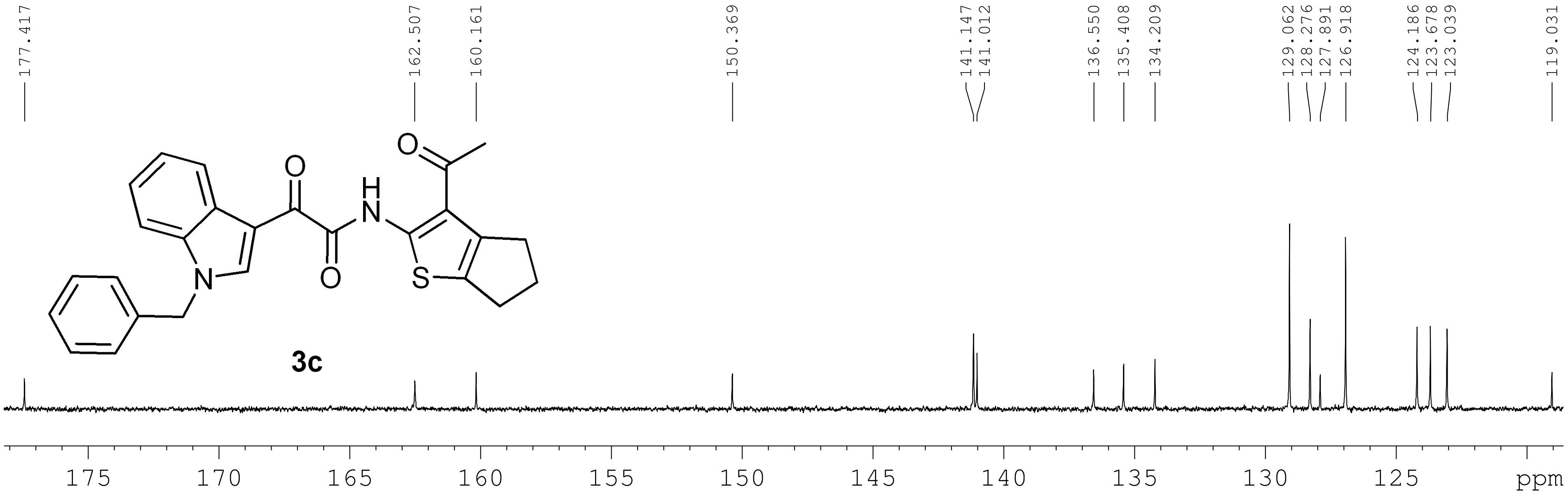

3c

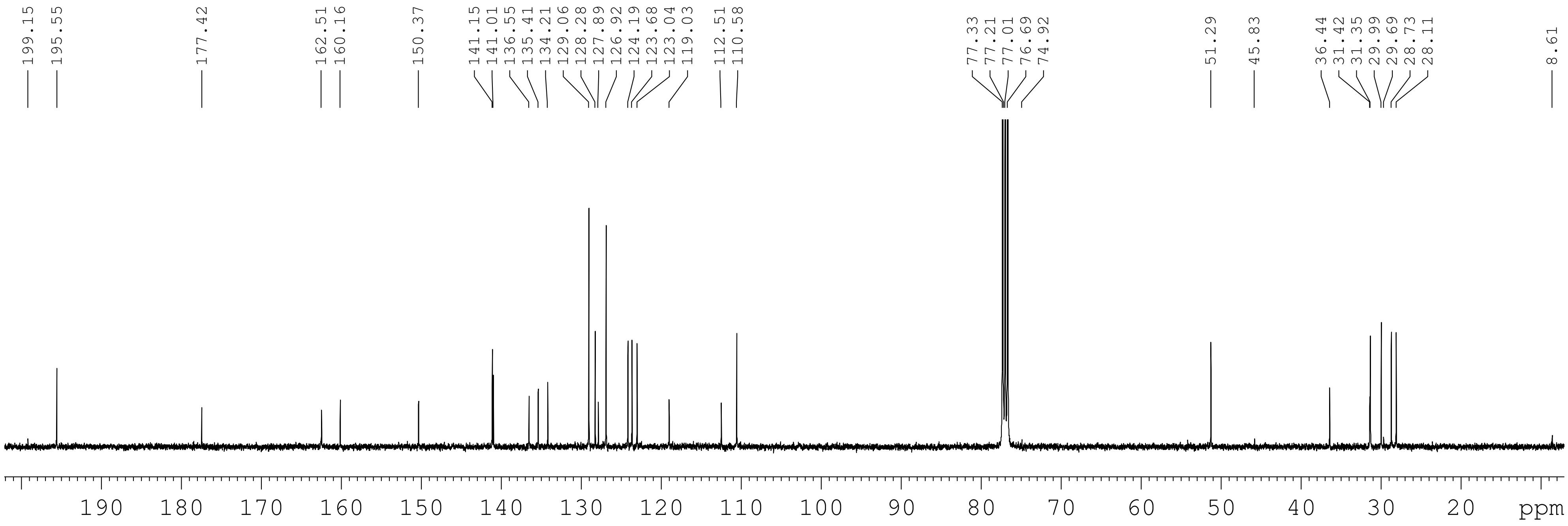

Mass Spectrum SmartFormula Report

Analysis Info

Analysis Name	D:\Data\2018\180920000002.d	Acquisition Date	9/20/2018 3:05:08 PM
Method	Tuneposlow.m	Operator	fabien
Sample Name	180913799///EE65	Instrument	micrOTOF-Q 228888.00098
Comment	CH2Cl2/MeOH		

Acquisition Parameter

Source Type	ESI	Ion Polarity	Positive	Set Nebulizer	1.0 Bar
Focus	Not active	Set Capillary	4500 V	Set Dry Heater	190 °C
Scan Begin	50 m/z	Set End Plate Offset	-500 V	Set Dry Gas	4.0 l/min
Scan End	3000 m/z	Set Collision Cell RF	250.0 Vpp	Set Divert Valve	Source

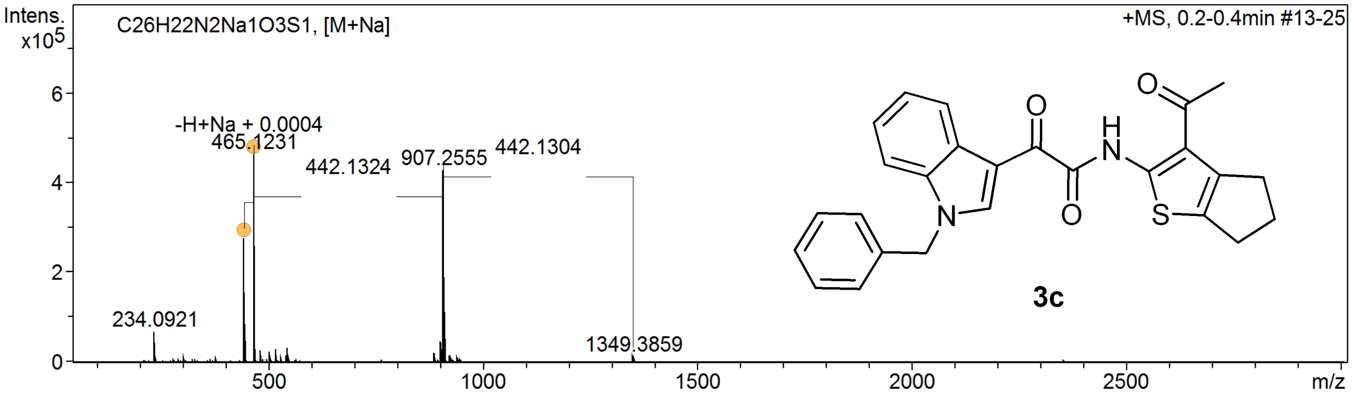

Meas. m/z	#	Ion Formula	m/z	|err| [mDa]	err [ppm]	mSigma	# mSigma	Score	rdb	e⁻ Conf	N-Rule
443.1407	1	C26H23N2O3S	443.1424	1.7	3.7	3.5	1	100.00	16.5	even	ok
465.1231	1	C26H22N2NaO3S	465.1243	1.3	2.7	1.7	1	100.00	16.5	even	ok

L2CM Salma EE-45

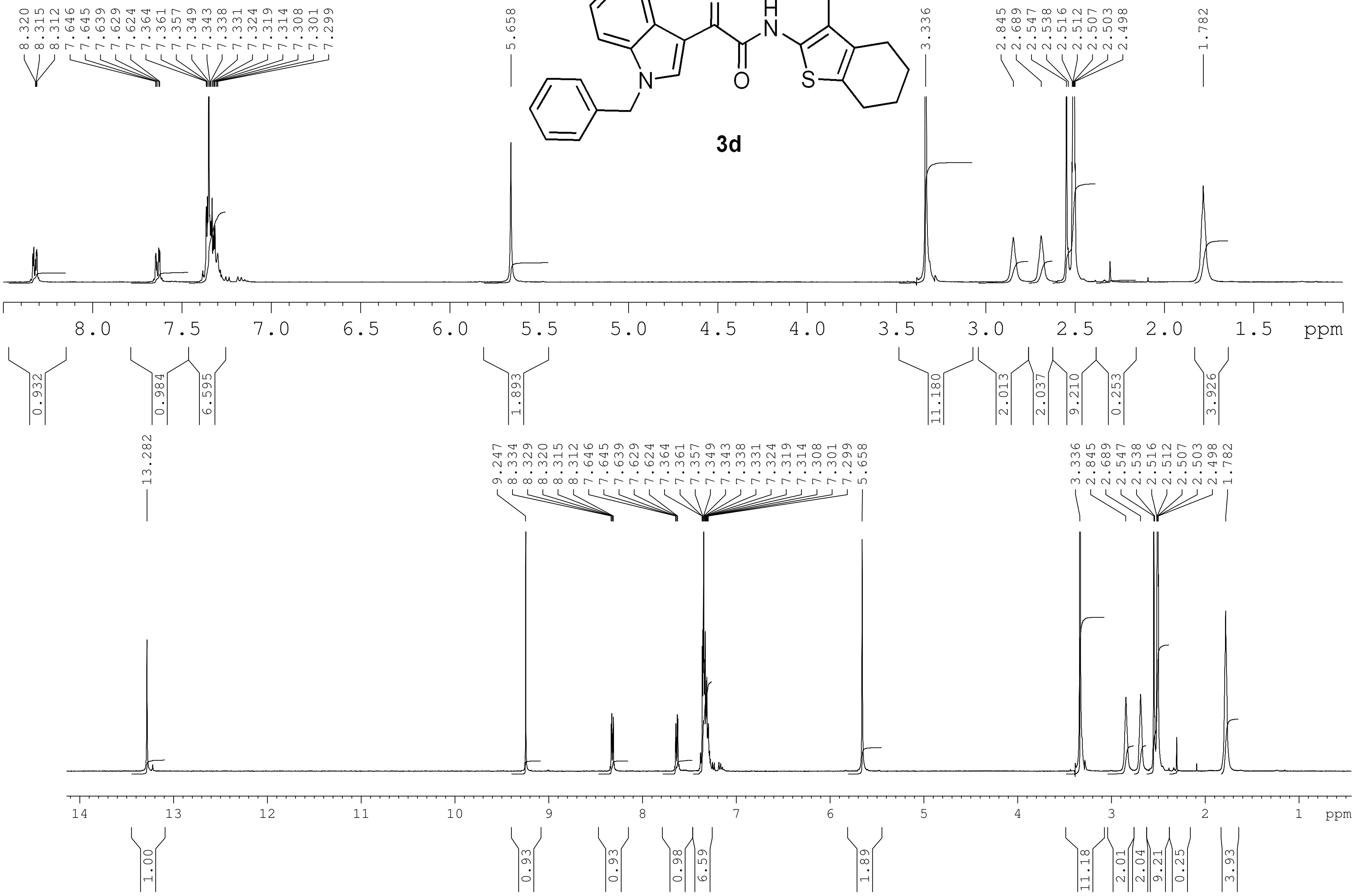

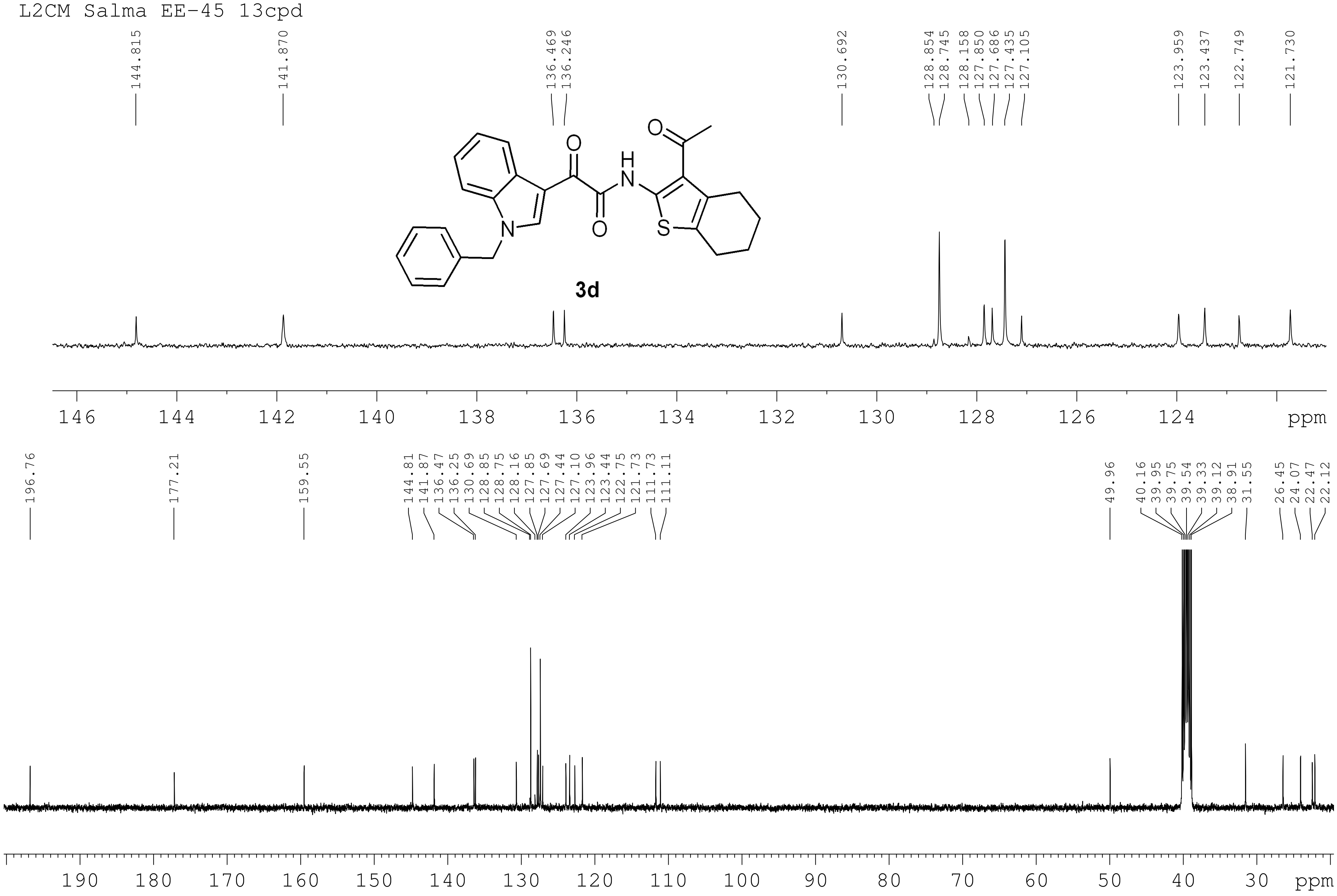
L2CM Salma EE-45 13cpd
144.815
141.870
136.469
136.246
130.692
128.854
128.745
128.158
127.850
127.686
127.435
127.105
123.959
123.437
122.749
121.730
O
O
H
N
O
N
S
3d
146 144 142 140 138 136 134 132 130 128 126 124 ppm
196.76
177.21
159.55
144.81
141.87
136.47
136.25
130.69
128.85
128.75
128.16
127.85
127.69
127.44
127.10
123.96
123.44
122.75
121.73
111.73
111.11
49.96
40.16
39.95
39.75
39.54
39.33
39.12
38.91
31.55
26.45
24.07
22.47
22.12
190 180 170 160 150 140 130 120 110 100 90 80 70 60 50 40 30 ppm

Mass Spectrum SmartFormula Report

Analysis Info

Analysis Name	D:\Data\2018\180920000003.d	Acquisition Date	9/20/2018 3:21:17 PM
Method	Tuneposlow.m	Operator	fabien
Sample Name	180913800///EE45	Instrument	micrOTOF-Q 228888.00098
Comment	CH2Cl2/MeOH		

Acquisition Parameter

Source Type	ESI	Ion Polarity	Positive	Set Nebulizer	1.0 Bar
Focus	Not active	Set Capillary	4500 V	Set Dry Heater	190 °C
Scan Begin	50 m/z	Set End Plate Offset	-500 V	Set Dry Gas	4.0 l/min
Scan End	3000 m/z	Set Collision Cell RF	250.0 Vpp	Set Divert Valve	Source

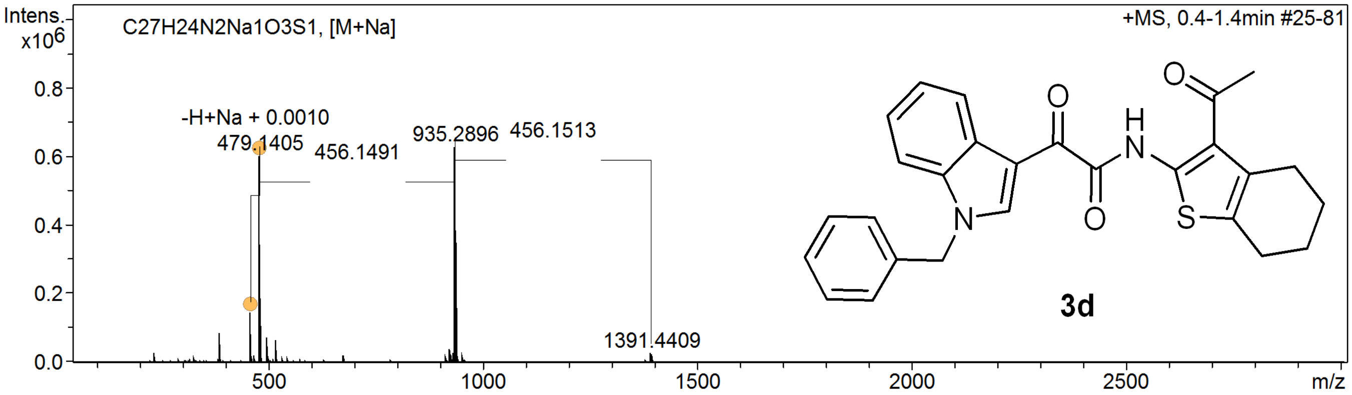

3d

Meas. m/z	#	Ion Formula	m/z	|err| [mDa]	err [ppm]	mSigma	# mSigma	Score	rdb	e⁻ Conf	N-Rule
457.1576	1	C27H25N2O3S	457.1580	0.4	0.9	4.3	1	100.00	16.5	even	ok
479.1405	1	C27H24N2NaO3S	479.1400	0.6	-1.2	1.5	1	100.00	16.5	even	ok

L2CM Salma AE-47

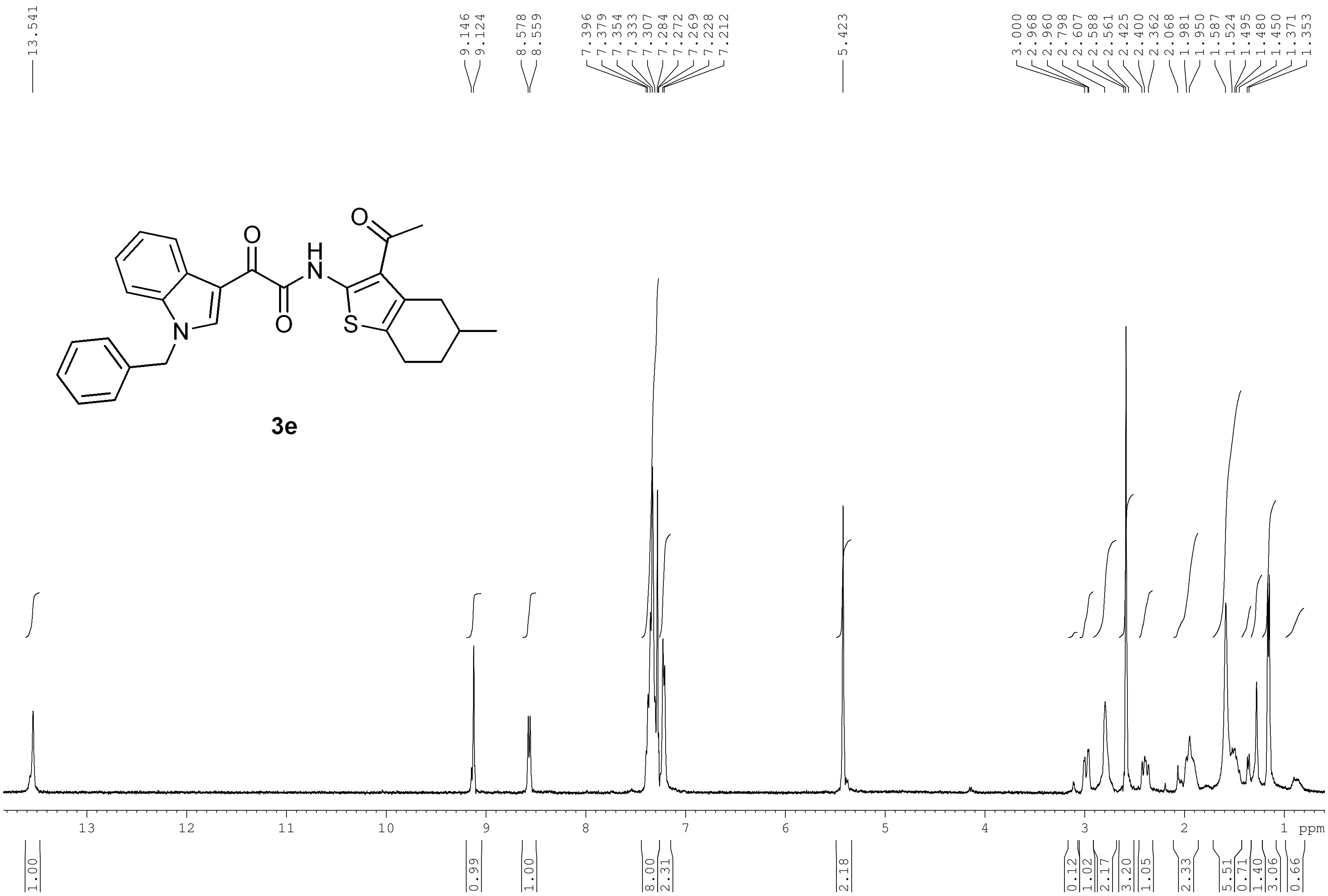

L2CM Salma EE-47 13C

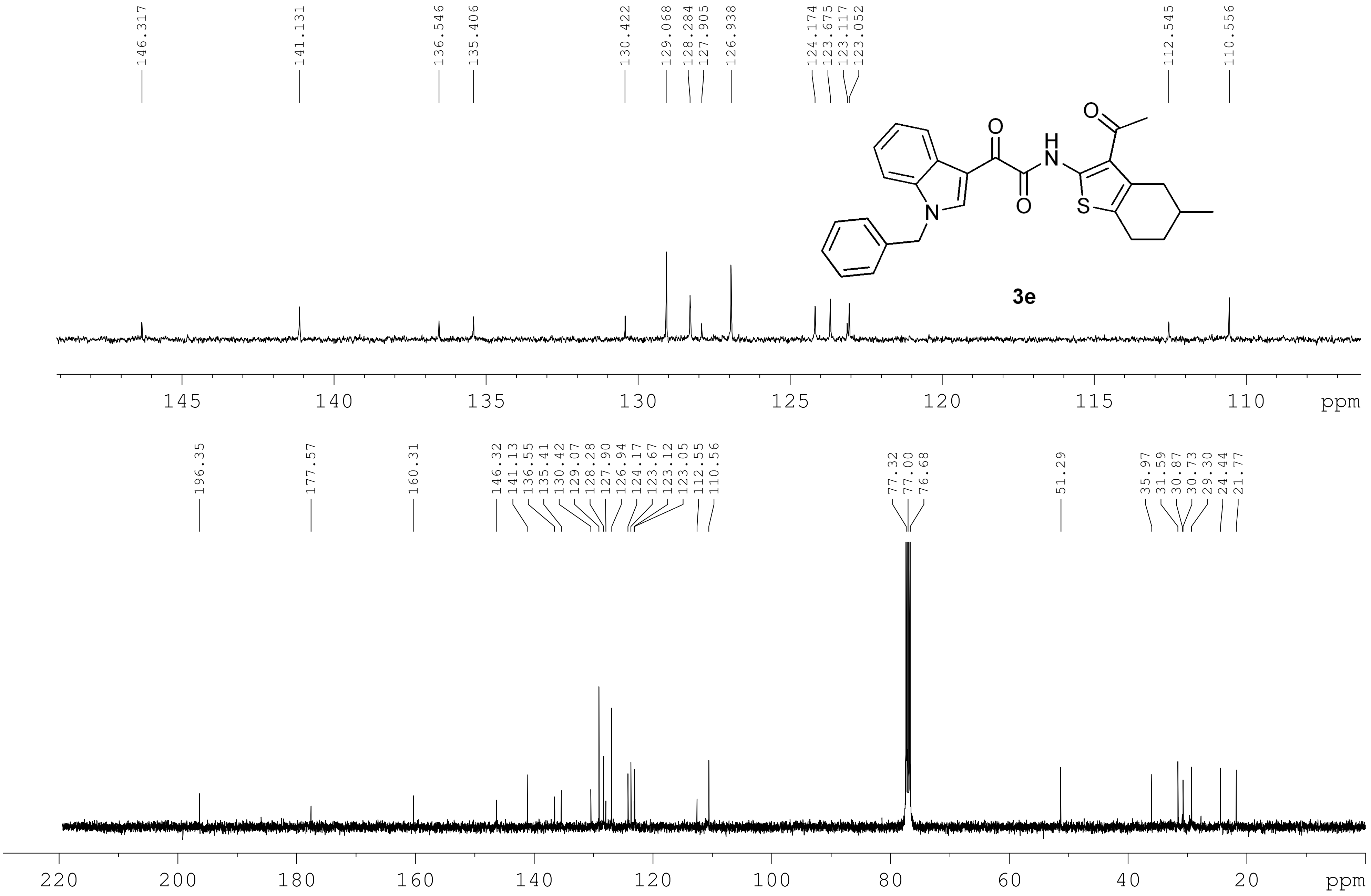

Mass Spectrum SmartFormula Report

Analysis Info

Analysis Name	D:\Data\2018\180920000004.d	Acquisition Date	9/20/2018 3:36:36 PM
Method	Tuneposlow.m	Operator	fabien
Sample Name	180913801///EE47	Instrument	micrOTOF-Q 228888.00098
Comment	CH2Cl2/MeOH		

Acquisition Parameter

Source Type	ESI	Ion Polarity	Positive	Set Nebulizer	1.0 Bar
Focus	Not active	Set Capillary	4500 V	Set Dry Heater	190 °C
Scan Begin	50 m/z	Set End Plate Offset	-500 V	Set Dry Gas	4.0 l/min
Scan End	3000 m/z	Set Collision Cell RF	250.0 Vpp	Set Divert Valve	Source

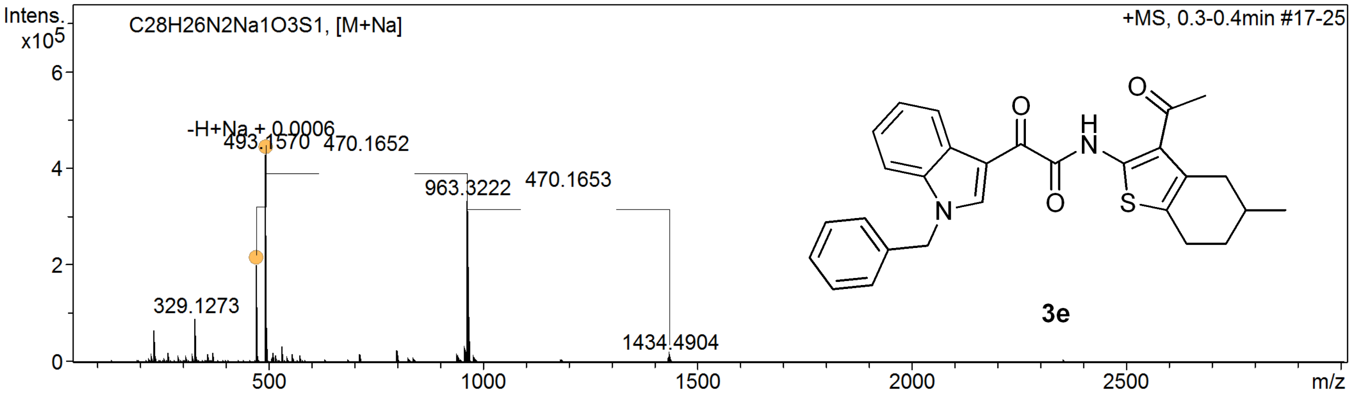

Meas. m/z	#	Ion Formula	m/z	|err| [mDa]	err [ppm]	mSigma	# mSigma	Score	rdb	e⁻ Conf	N-Rule
471.1744	1	C28H27N2O3S	471.1737	0.7	-1.5	5.5	1	100.00	16.5	even	ok
493.1570	1	C28H26N2NaO3S	493.1556	1.4	-2.8	5.7	1	100.00	16.5	even	ok

L2CM Salma EE-46
L2CM Salma EE-46

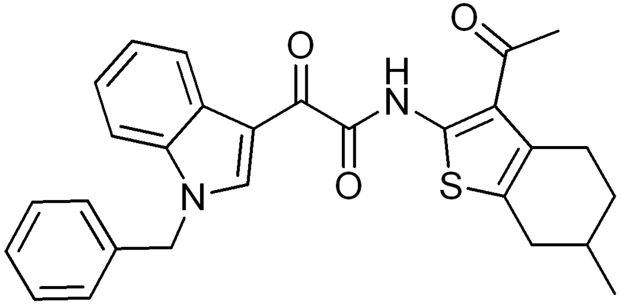

3f

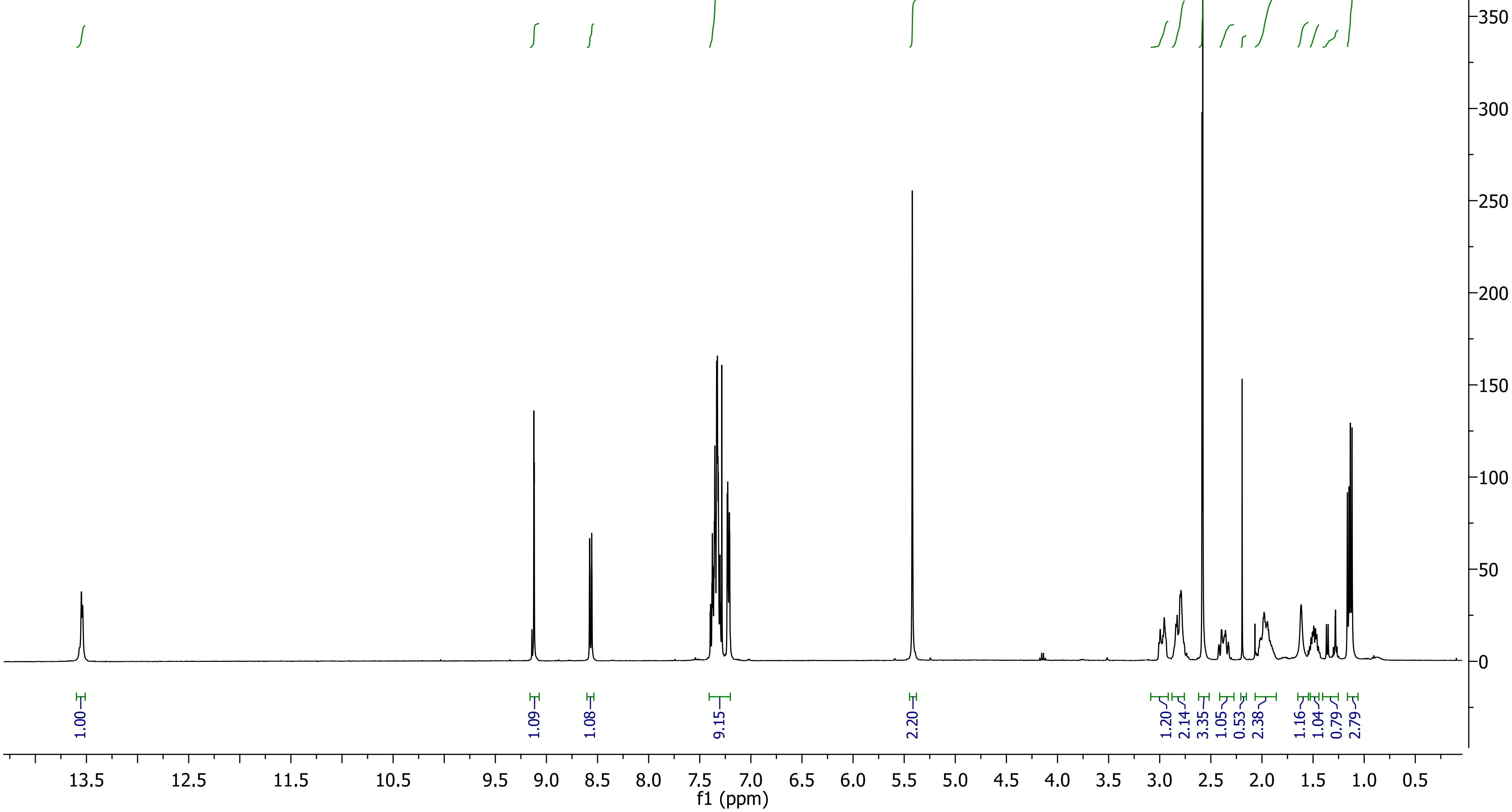

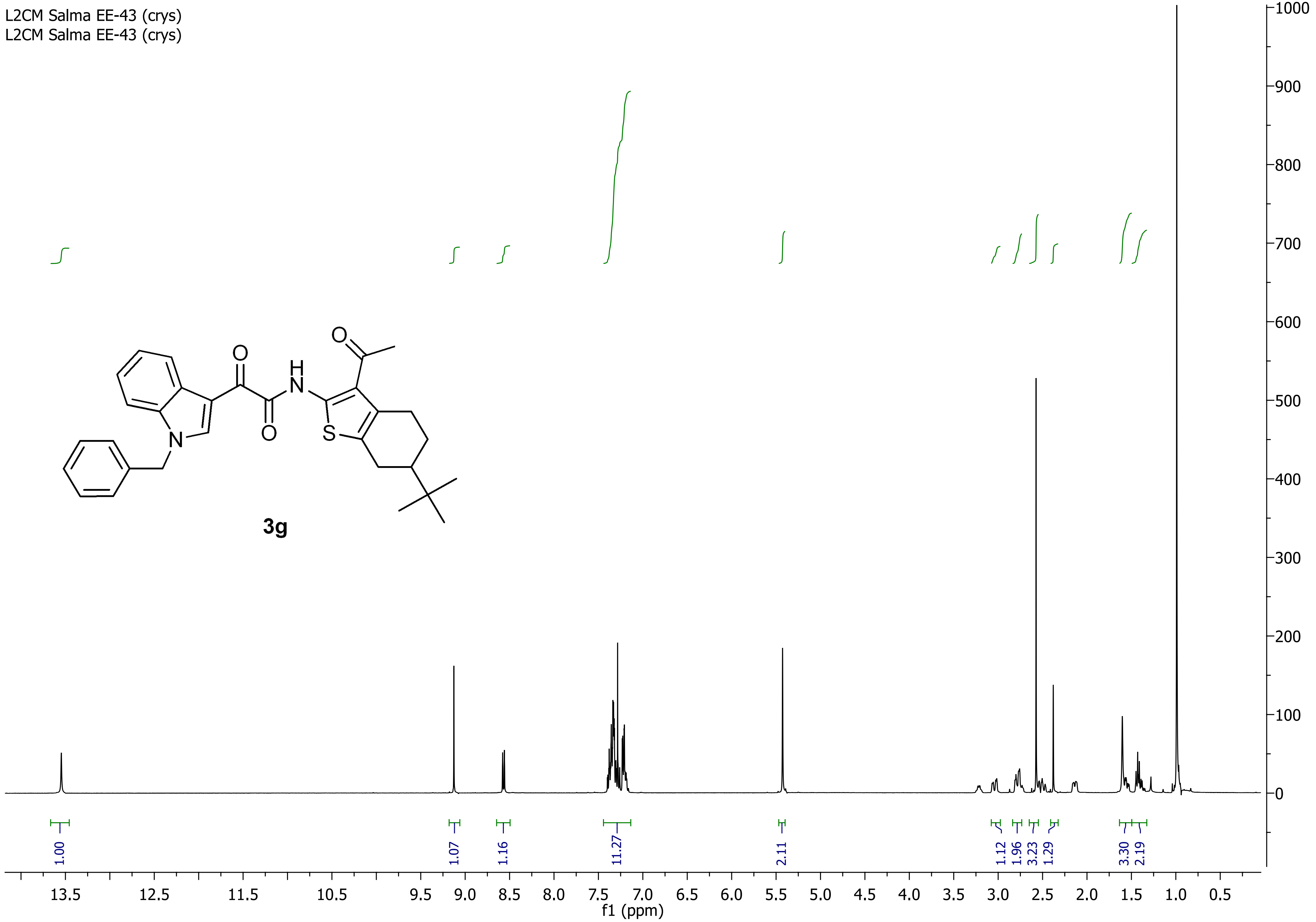
L2CM Salma EE-43 (crys)
L2CM Salma EE-43 (crys)
3g
O
O
O
H
N
N
S
1.00
1.07
1.16
11.27
2.11
1.12
1.96
3.23
1.29
3.30
2.19
1000
900
800
700
600
500
400
300
200
100
0
13.5
12.5
11.5
10.5
9.5
9.0
8.5
8.0
7.5
7.0
6.5
6.0
5.5
5.0
4.5
4.0
3.5
3.0
2.5
2.0
1.5
1.0
0.5
f1 (ppm)

L2CM Salma EE-43 (crys) 13C

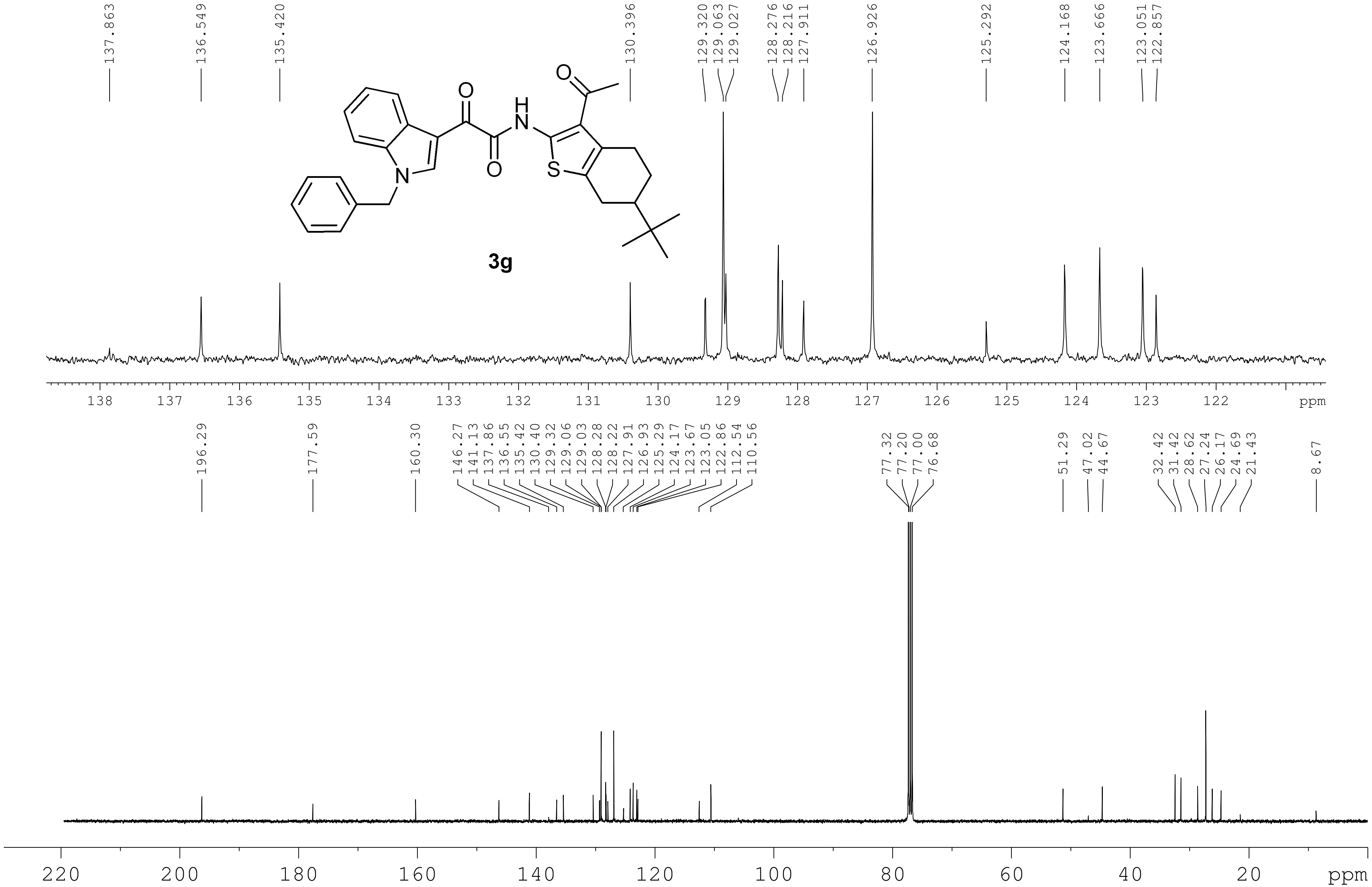

Display Report

Analysis Info

Analysis Name	D:\Data\2018\180920000005.d	Acquisition Date	9/20/2018 3:56:39 PM
Method	Tuneposlow.m	Operator	fabien
Sample Name	180913802///EE43	Instrument	micrOTOF-Q 228888.00098
Comment	CH2Cl2/MeOH		

Acquisition Parameter

Source Type	ESI	Ion Polarity	Positive	Set Nebulizer	1.0 Bar
Focus	Not active	Set Capillary	4500 V	Set Dry Heater	190 °C
Scan Begin	50 m/z	Set End Plate Offset	-500 V	Set Dry Gas	4.0 l/min
Scan End	3000 m/z	Set Collision Cell RF	250.0 Vpp	Set Divert Valve	Source

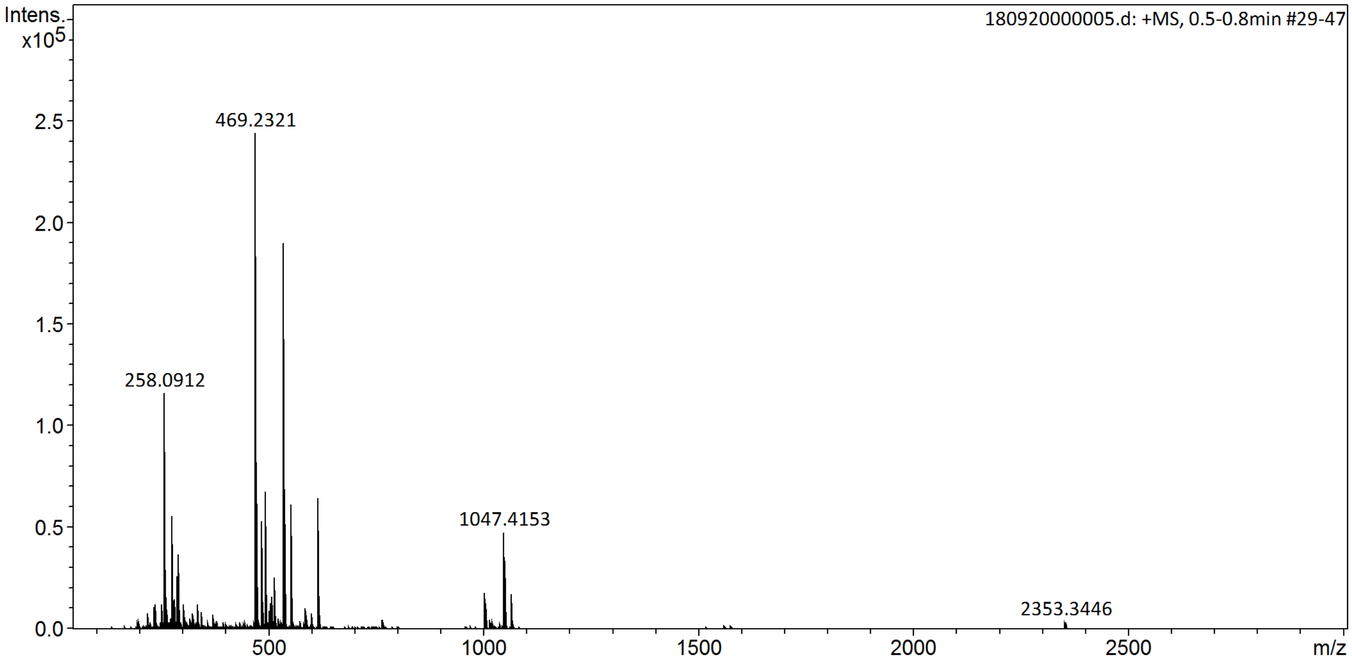

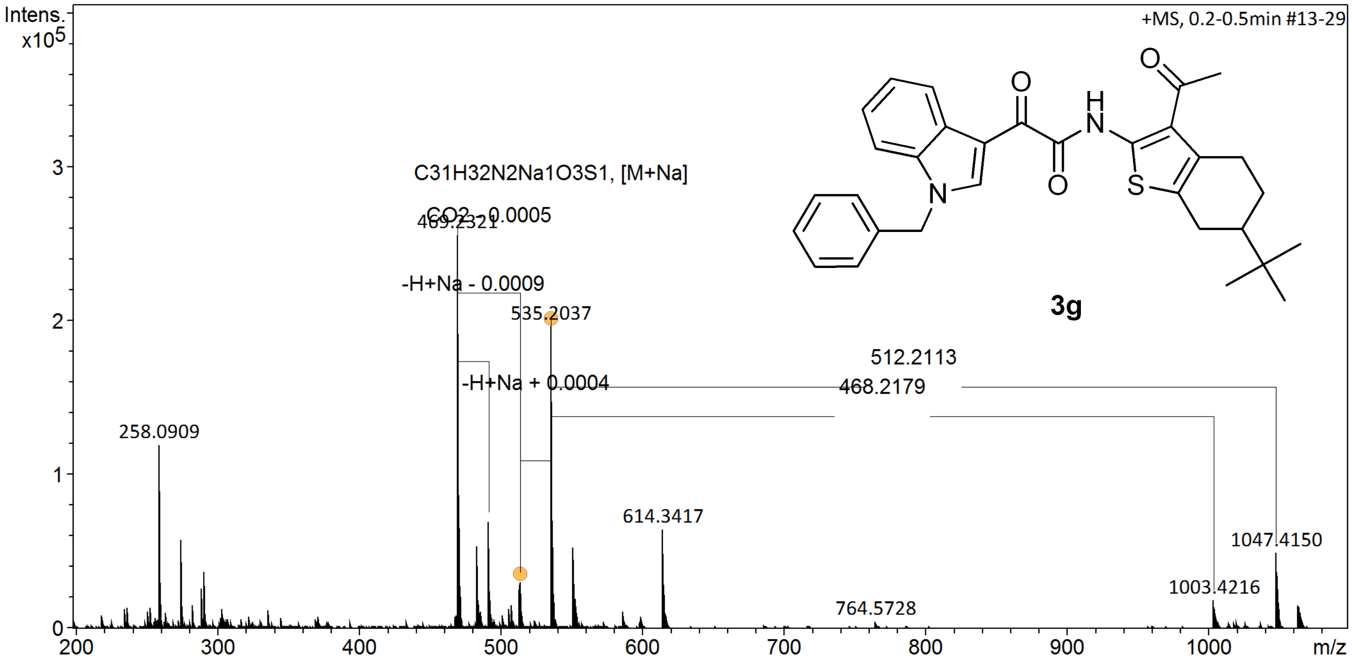

L2CM Salma EE-67
L2CM Salma EE-67

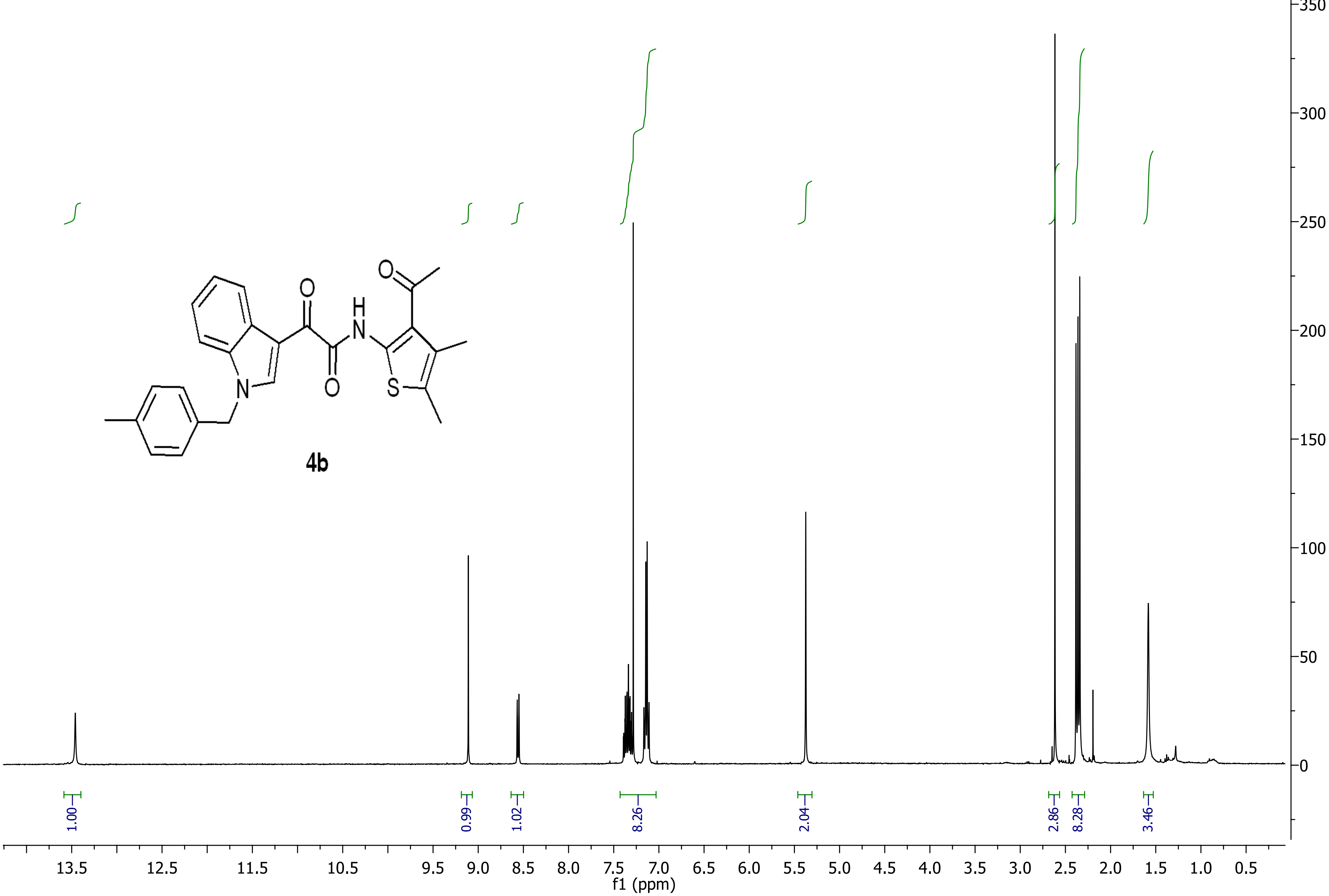

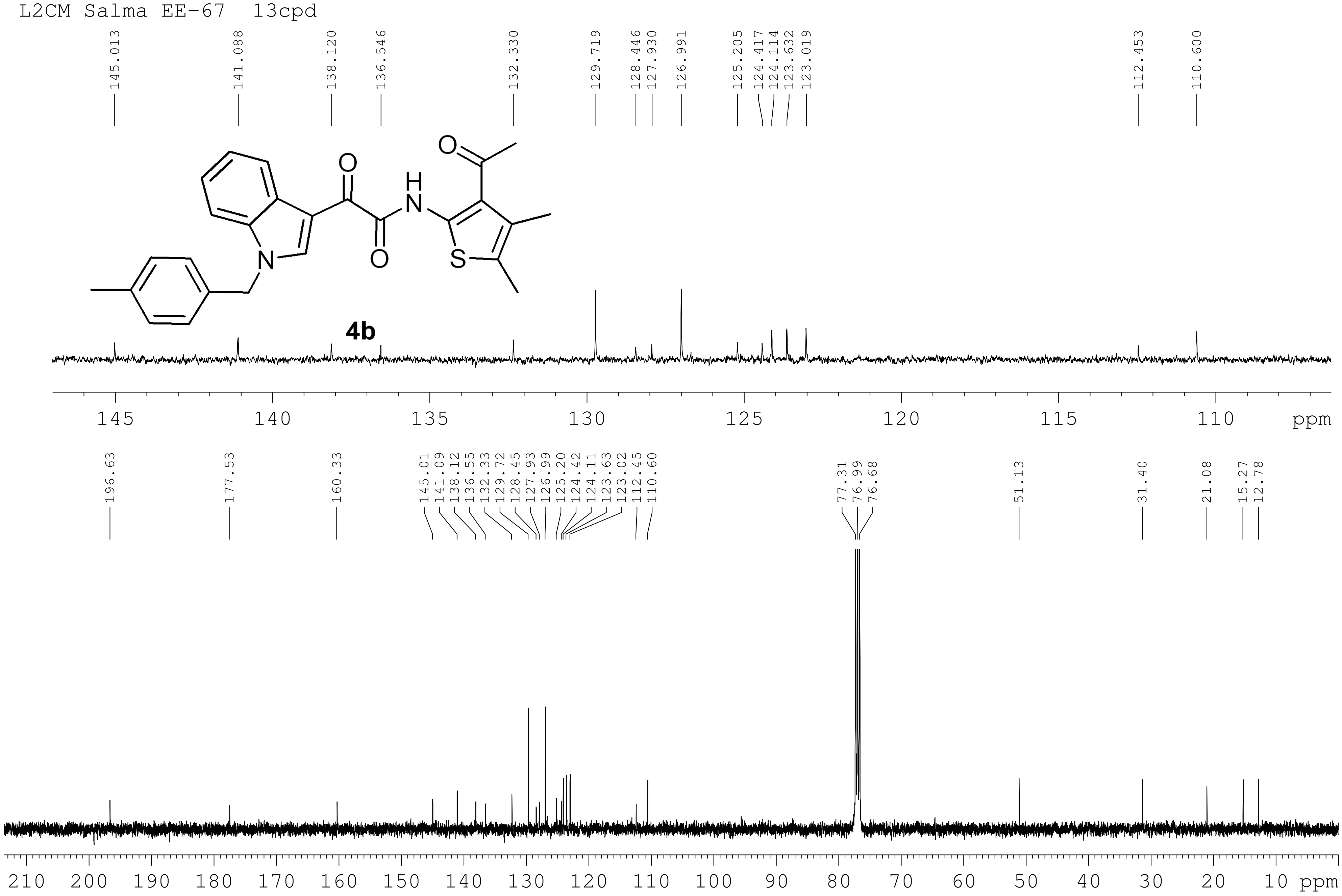
L2CM Salma EE-67 13cpd
145.013
141.088
138.120
136.546
132.330
129.719
128.446
127.930
126.991
125.205
124.417
124.114
123.632
123.019
112.453
110.600
H
N
O
O
O
N
S
4b
145 140 135 130 125 120 115 110 ppm
196.63
177.53
160.33
145.01
141.09
138.12
136.55
132.33
129.72
128.45
127.93
126.99
125.20
124.42
124.11
123.63
123.02
112.45
110.60
77.31
76.99
76.68
51.13
31.40
21.08
15.27
12.78
210 200 190 180 170 160 150 140 130 120 110 100 90 80 70 60 50 40 30 20 10 ppm

Mass Spectrum SmartFormula Report

Analysis Info

Analysis Name	D:\Data\2018\180920000007.d
Method	Tuneposlow.m
Sample Name	180913804///EE67
Comment	CH2Cl2/MeOH

Acquisition Date	9/20/2018 4:41:19 PM
Operator	fabien
Instrument	micrOTOF-Q 228888.00098

Acquisition Parameter

Source Type	ESI	Ion Polarity	Positive	Set Nebulizer	1.0 Bar
Focus	Not active	Set Capillary	4500 V	Set Dry Heater	190 °C
Scan Begin	50 m/z	Set End Plate Offset	-500 V	Set Dry Gas	4.0 l/min
Scan End	3000 m/z	Set Collision Cell RF	250.0 Vpp	Set Divert Valve	Source

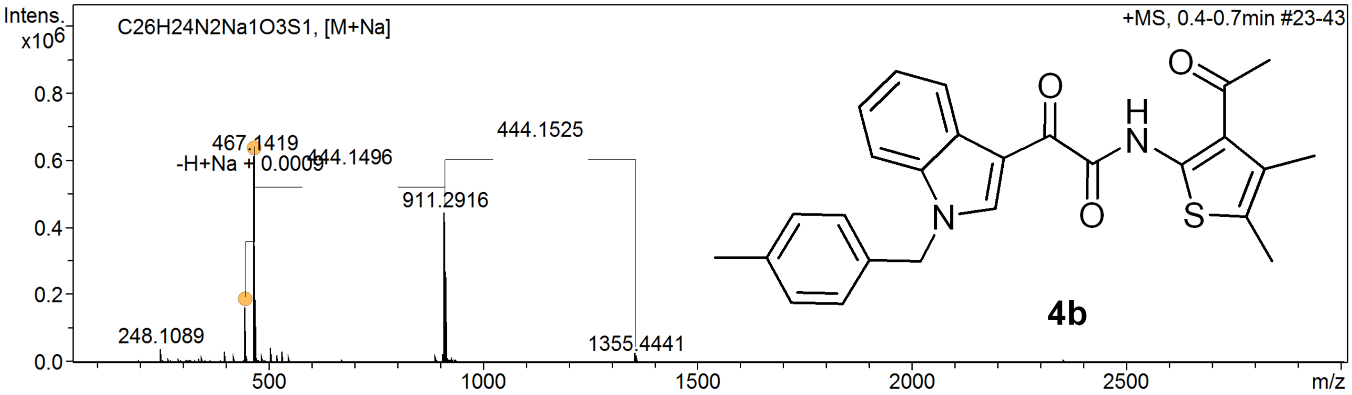

Meas. m/z	#	Ion Formula	m/z	|err| [mDa]	err [ppm]	mSigma	# mSigma	Score	rdb	e⁻ Conf	N-Rule
445.1590	1	C26H25N2O3S	445.1580	1.0	-2.2	2.2	1	100.00	15.5	even	ok
467.1419	1	C26H24N2NaO3S	467.1400	1.9	-4.1	2.4	1	100.00	15.5	even	ok

L2CM Salma EE-119
L2CM Salma EE-119

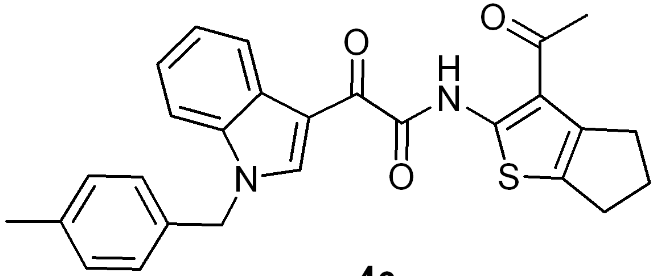

4c

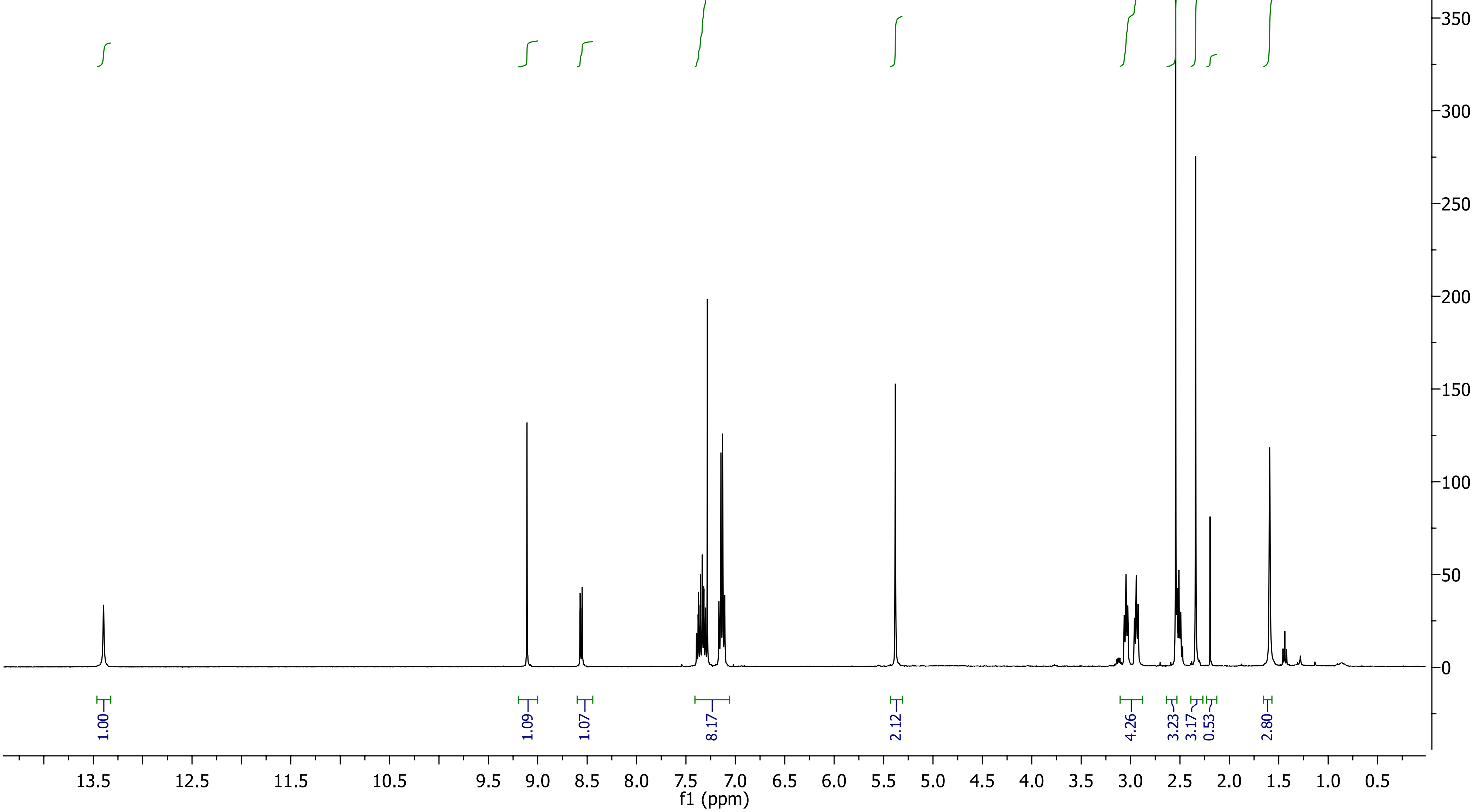

L2CM Salma EE-119 13cpd

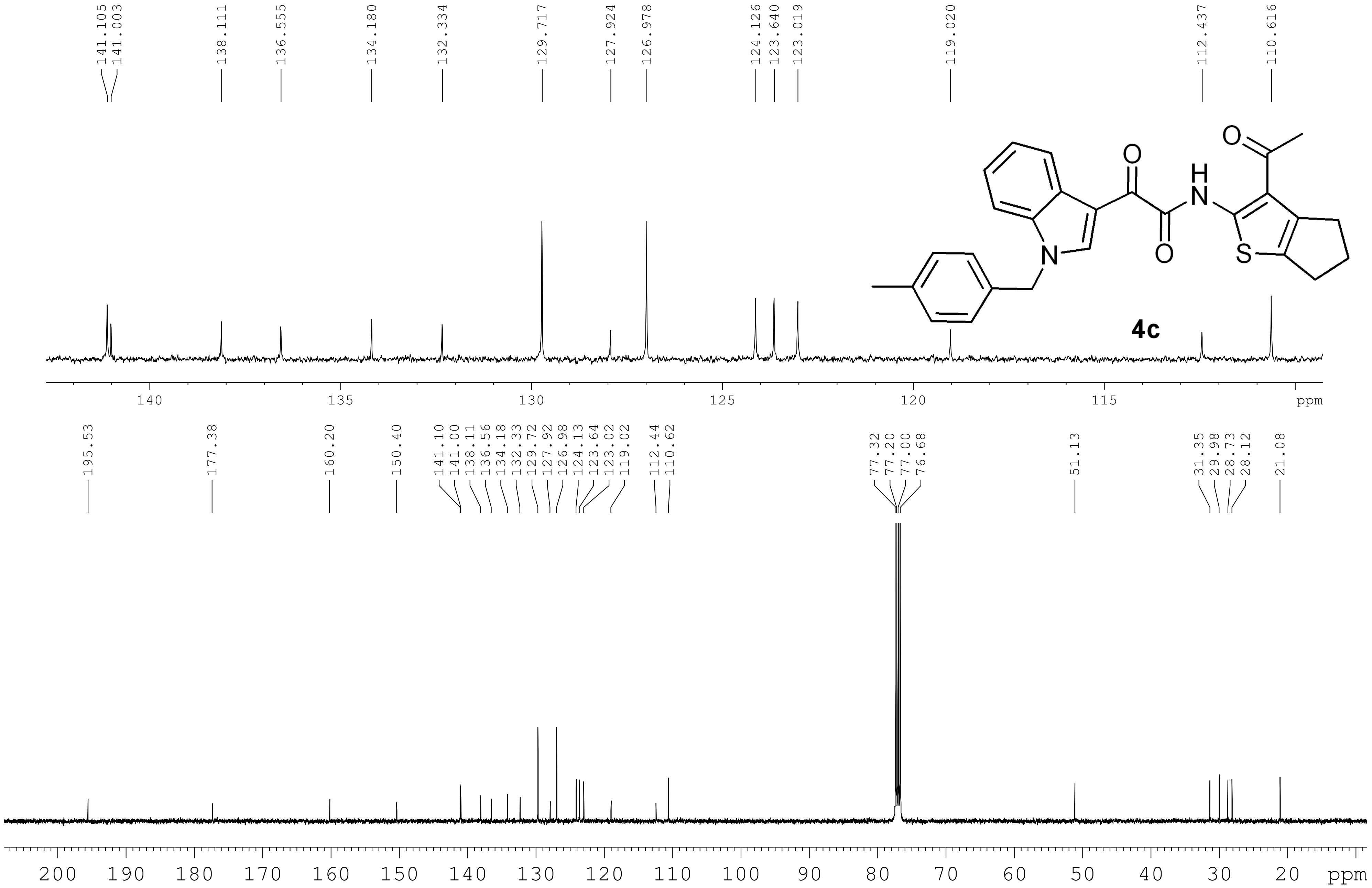

L2CM Salma EE-69

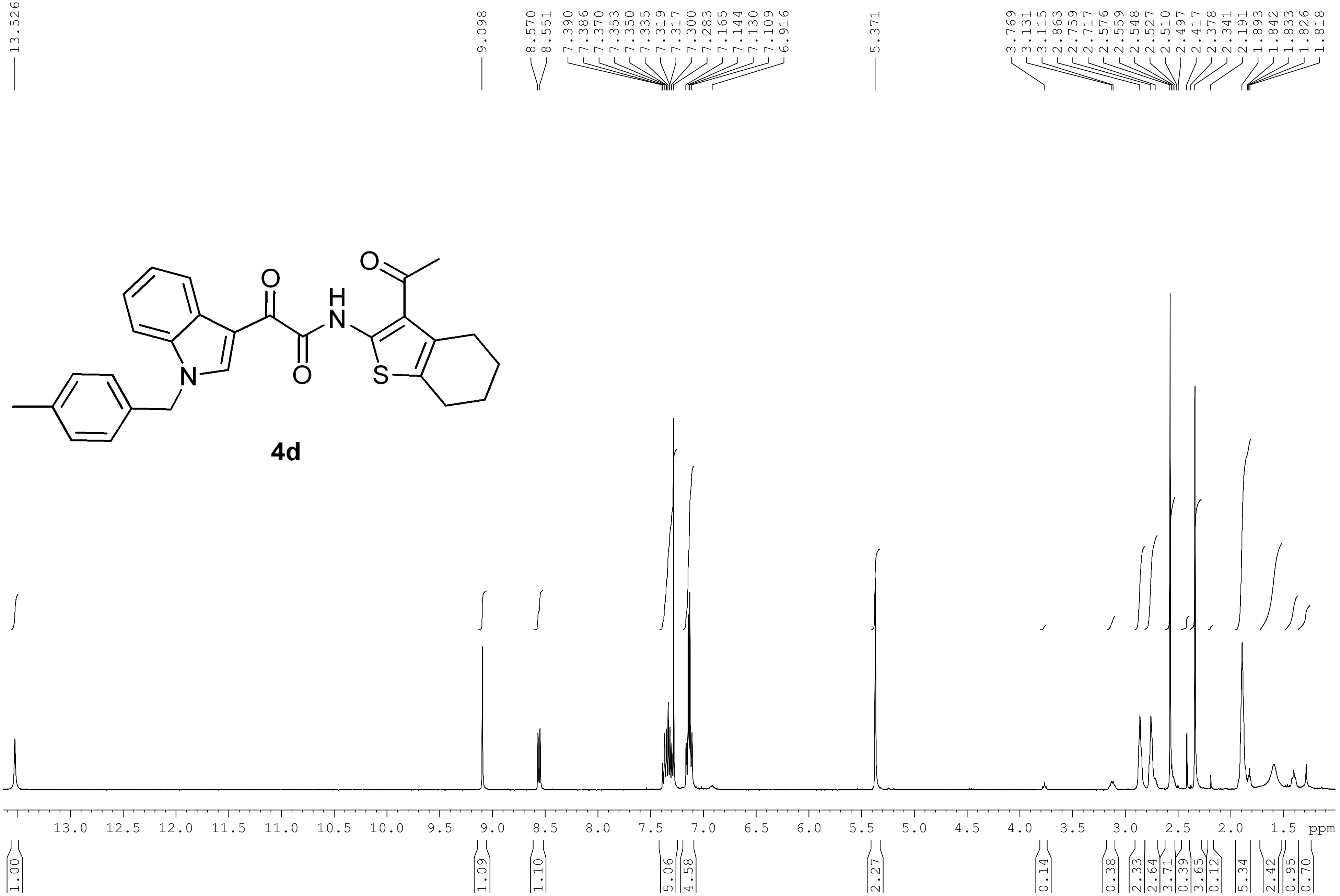

L2CM Salma EE-69 13C

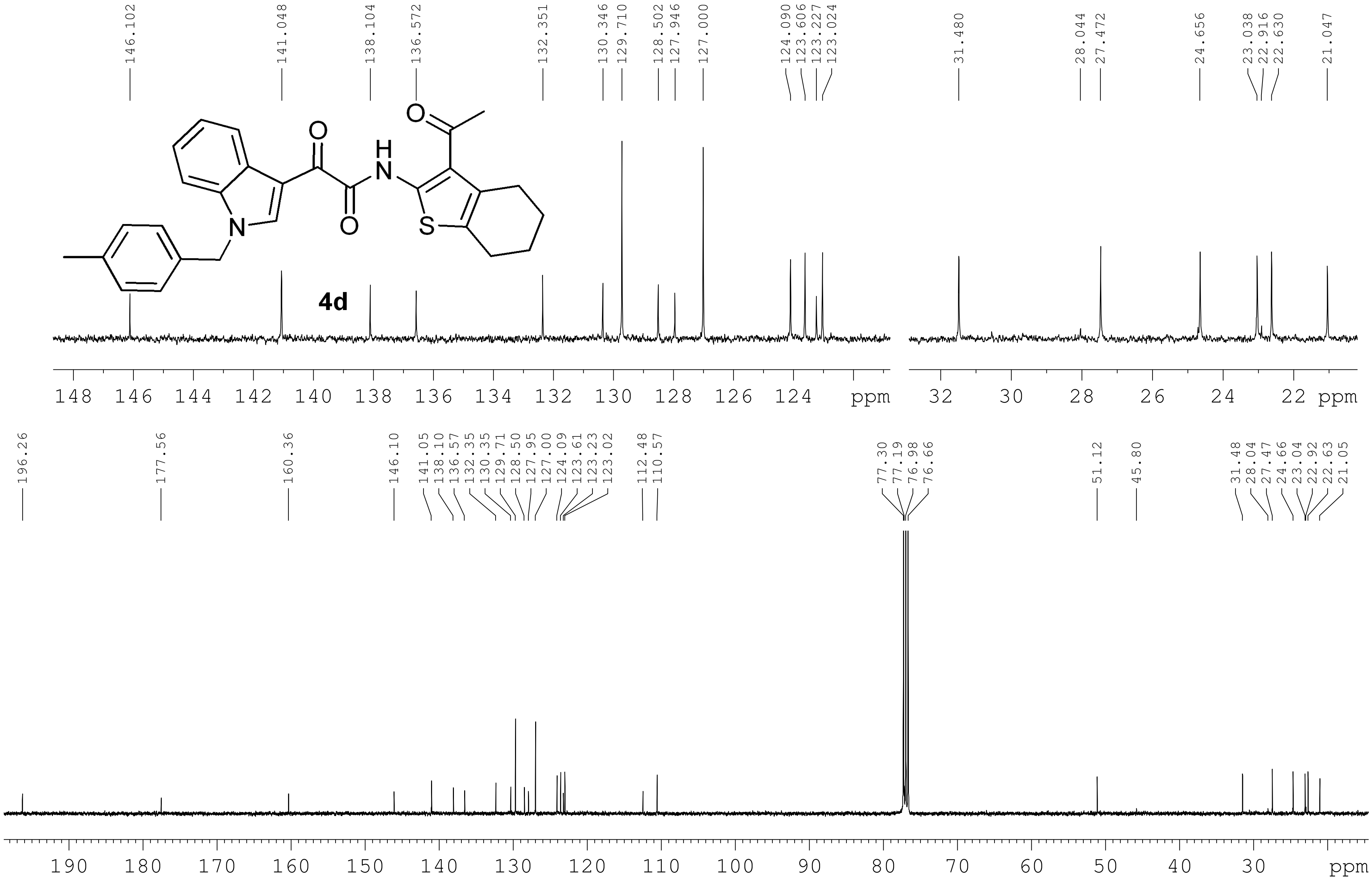

Mass Spectrum SmartFormula Report

Analysis Info

Analysis Name	D:\Data\2018\180924000001.d	Acquisition Date	9/24/2018 11:53:25 AM
Method	Tuneposlow.m	Operator	fabien
Sample Name	180913805///EE-69	Instrument	micrOTOF-Q 228888.00098
Comment	MeOH/CH2Cl2		

Acquisition Parameter

Source Type	ESI	Ion Polarity	Positive	Set Nebulizer	1.0 Bar
Focus	Not active	Set Capillary	4500 V	Set Dry Heater	190 °C
Scan Begin	50 m/z	Set End Plate Offset	-500 V	Set Dry Gas	4.0 l/min
Scan End	3000 m/z	Set Collision Cell RF	250.0 Vpp	Set Divert Valve	Source

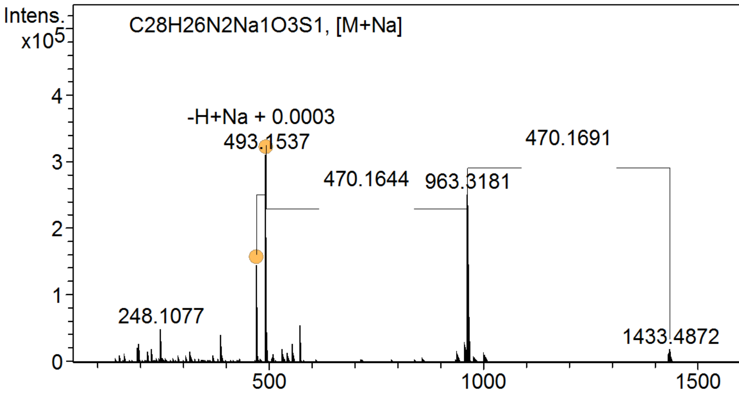

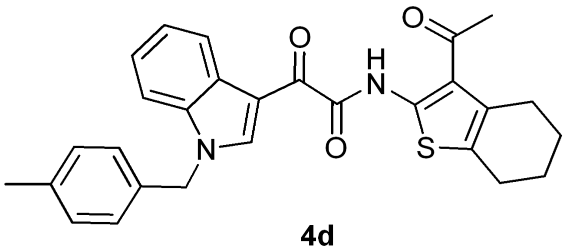

4d

Meas. m/z	#	Ion Formula	m/z	|err| [mDa]	err [ppm]	mSigma	# mSigma	Score	rdb	e⁻ Conf	N-Rule
471.1715	1	C28H27N2O3S	471.1737	2.2	4.7	3.7	1	100.00	16.5	even	ok
493.1537	1	C28H26N2NaO3S	493.1556	1.9	3.8	1.5	1	100.00	16.5	even	ok

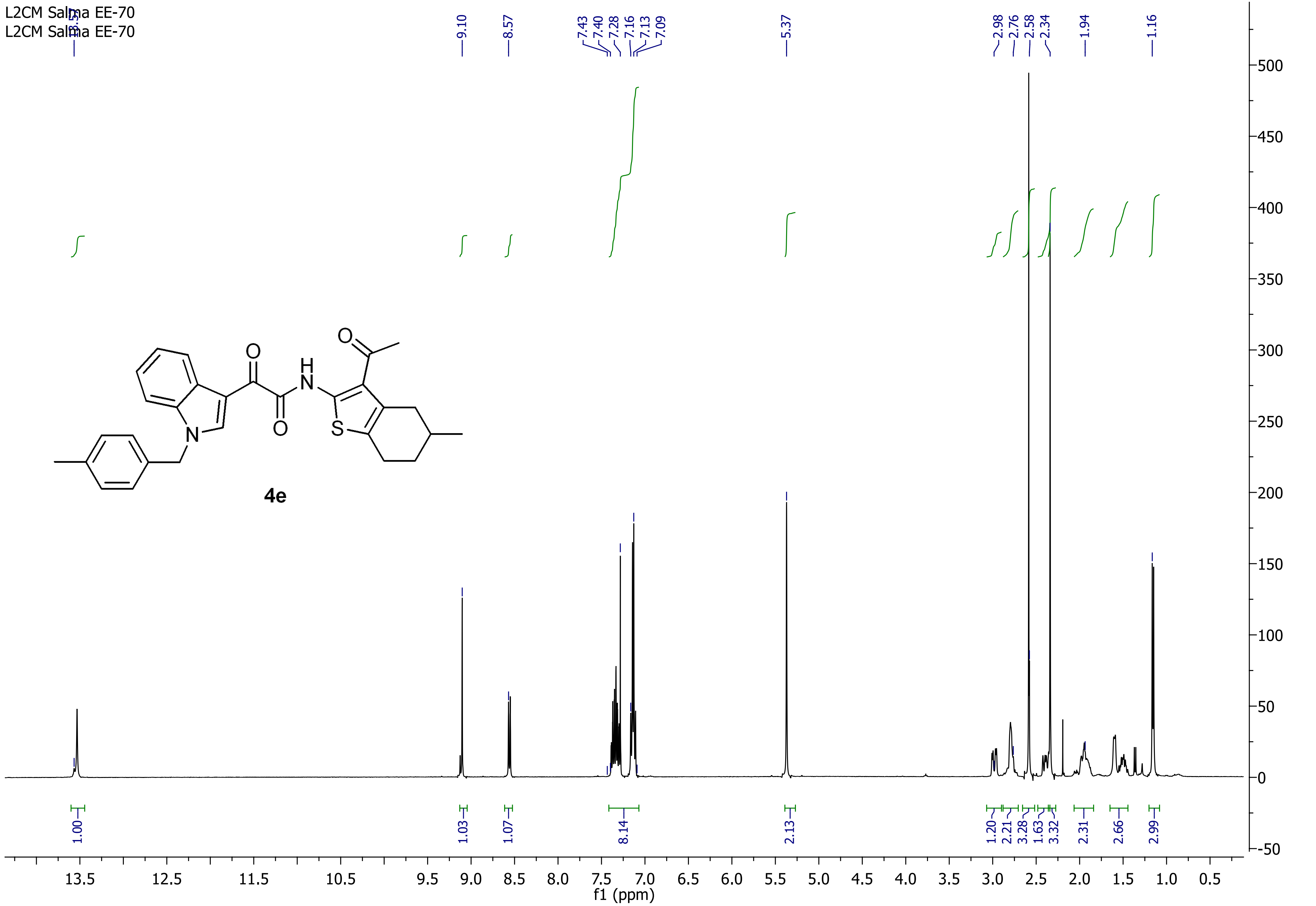

L2CM Salma EE-70
L2CM Salma EE-70
9.10
8.57
7.43
7.40
7.28
7.16
7.13
7.09
5.37
2.98
2.76
2.58
2.34
1.94
1.16
1.00
1.03
1.07
8.14
2.13
1.20
2.21
3.28
1.63
3.32
2.31
2.66
2.99
H
N
O
S
4e
f1 (ppm)

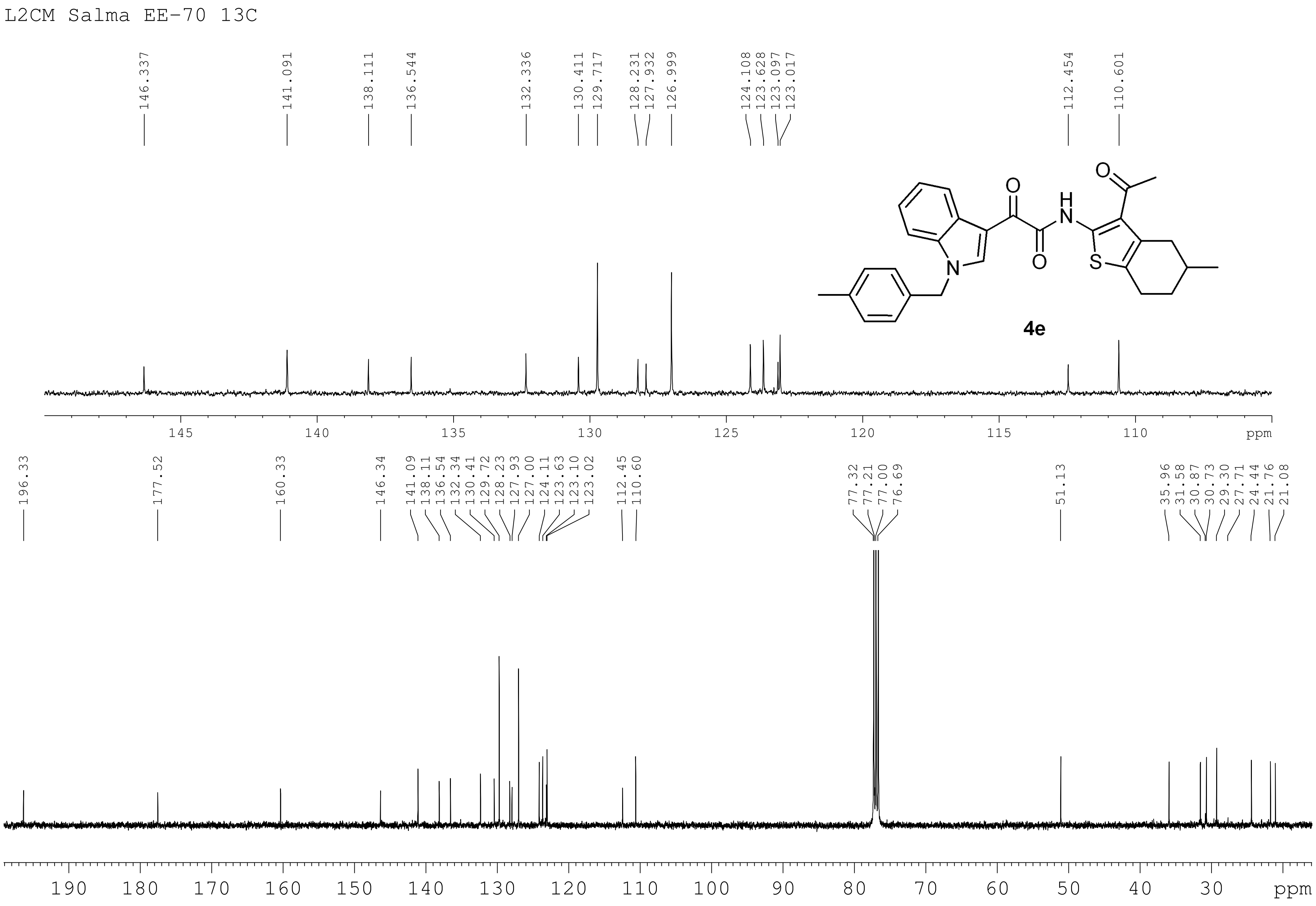
L2CM Salma EE-70 13C
146.337
141.091
138.111
136.544
132.336
130.411
129.717
128.231
127.932
126.999
124.108
123.628
123.097
123.017
112.454
110.601
4e
145
140
135
130
125
120
115
110
ppm
196.33
177.52
160.33
146.34
141.09
138.11
136.54
132.34
130.41
129.72
128.23
127.93
127.00
124.11
123.63
123.10
123.02
112.45
110.60
77.32
77.21
77.00
76.69
51.13
35.96
31.58
30.87
30.73
29.30
27.71
24.44
21.76
21.08
190
180
170
160
150
140
130
120
110
100
90
80
70
60
50
40
30
ppm

Mass Spectrum SmartFormula Report

Analysis Info

Analysis Name	D:\Data\2018\180924000002.d
Method	Tuneposlow.m
Sample Name	180913806///EE-70
Comment	MeOH/CH2Cl2

Acquisition Date	9/24/2018 12:02:18 PM
Operator	fabien
Instrument	micrOTOF-Q 228888.00098

Acquisition Parameter

Source Type	ESI	Ion Polarity	Positive	Set Nebulizer	1.0 Bar
Focus	Not active	Set Capillary	4500 V	Set Dry Heater	190 °C
Scan Begin	50 m/z	Set End Plate Offset	-500 V	Set Dry Gas	4.0 l/min
Scan End	3000 m/z	Set Collision Cell RF	250.0 Vpp	Set Divert Valve	Source

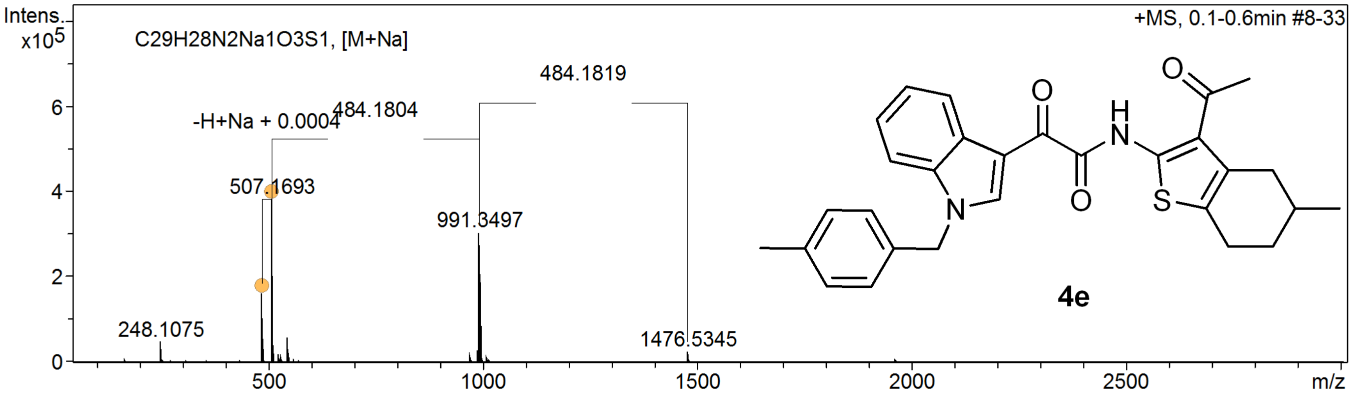

Meas. m/z	#	Ion Formula	m/z	|err| [mDa]	err [ppm]	mSigma	# mSigma	Score	rdb	e⁻ Conf	N-Rule
485.1869	1	C29H29N2O3S	485.1893	2.4	5.0	2.7	1	100.00	16.5	even	ok
507.1693	1	C29H28N2NaO3S	507.1713	2.0	4.0	2.6	1	100.00	16.5	even	ok

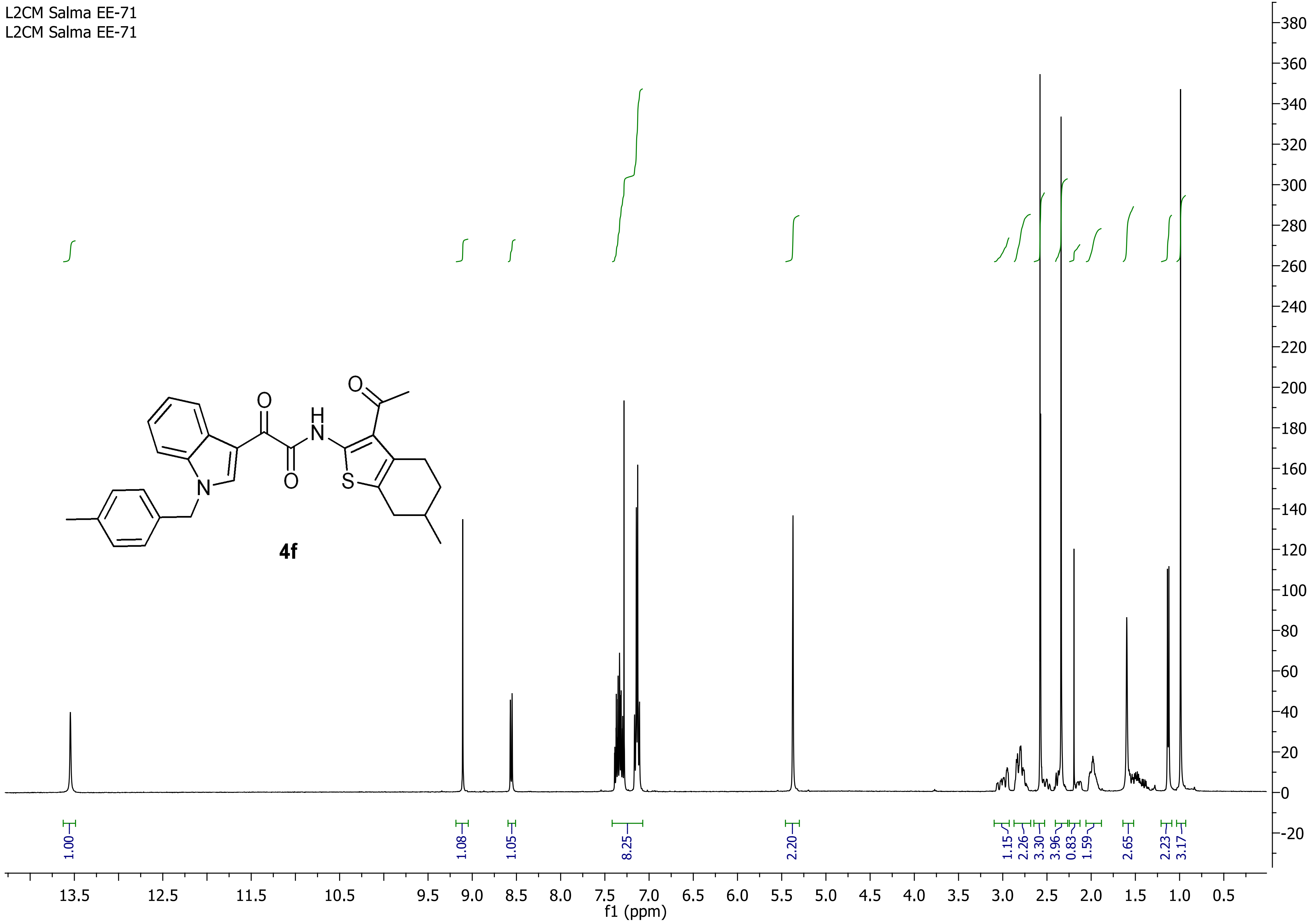

L2CM Salma EE-71
L2CM Salma EE-71
4f
O
O
O
H
N
N
S
1.00
1.08
1.05
8.25
2.20
1.15
2.26
3.30
3.96
0.83
1.59
2.65
2.23
3.17
13.5 12.5 11.5 10.5 9.5 9.0 8.5 8.0 7.5 7.0 6.5 6.0 5.5 5.0 4.5 4.0 3.5 3.0 2.5 2.0 1.5 1.0 0.5
f1 (ppm)
380 360 340 320 300 280 260 240 220 200 180 160 140 120 100 80 60 40 20 0 -20

L2CM Salma EE-71

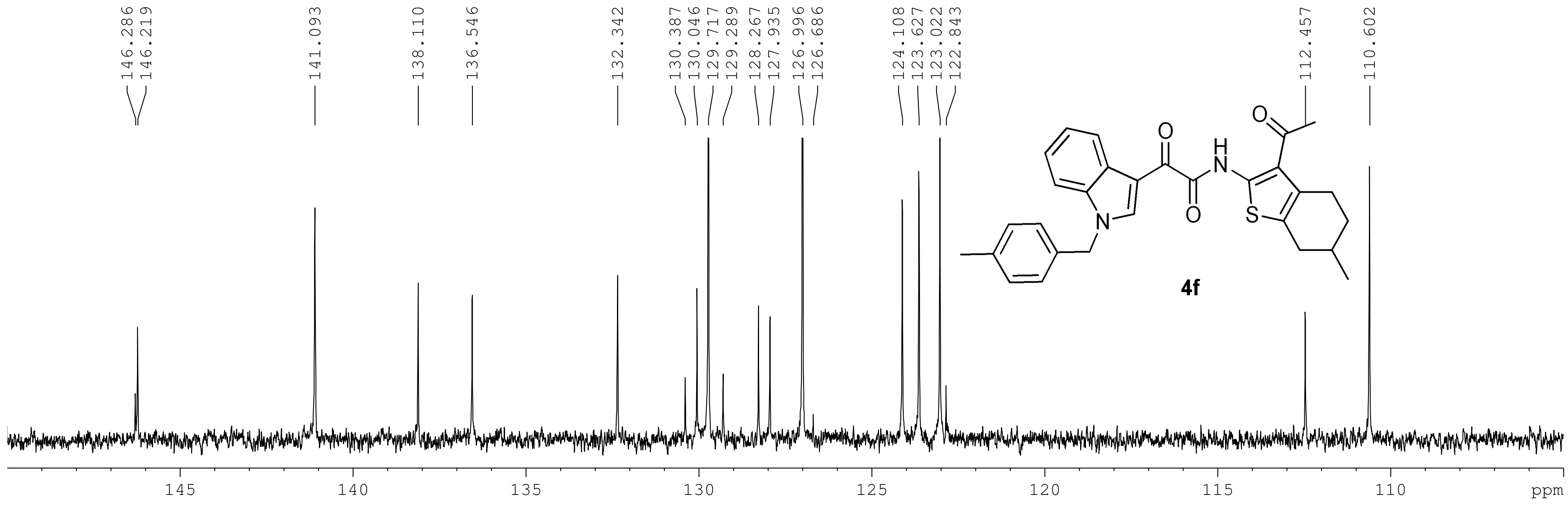

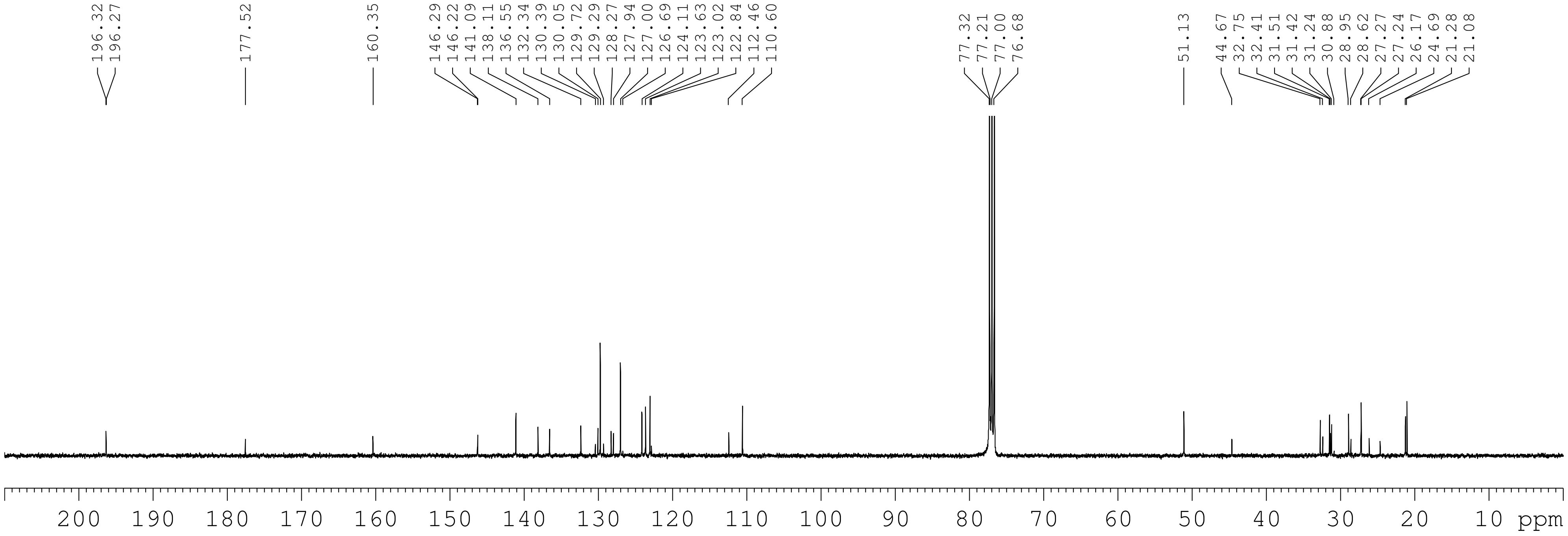

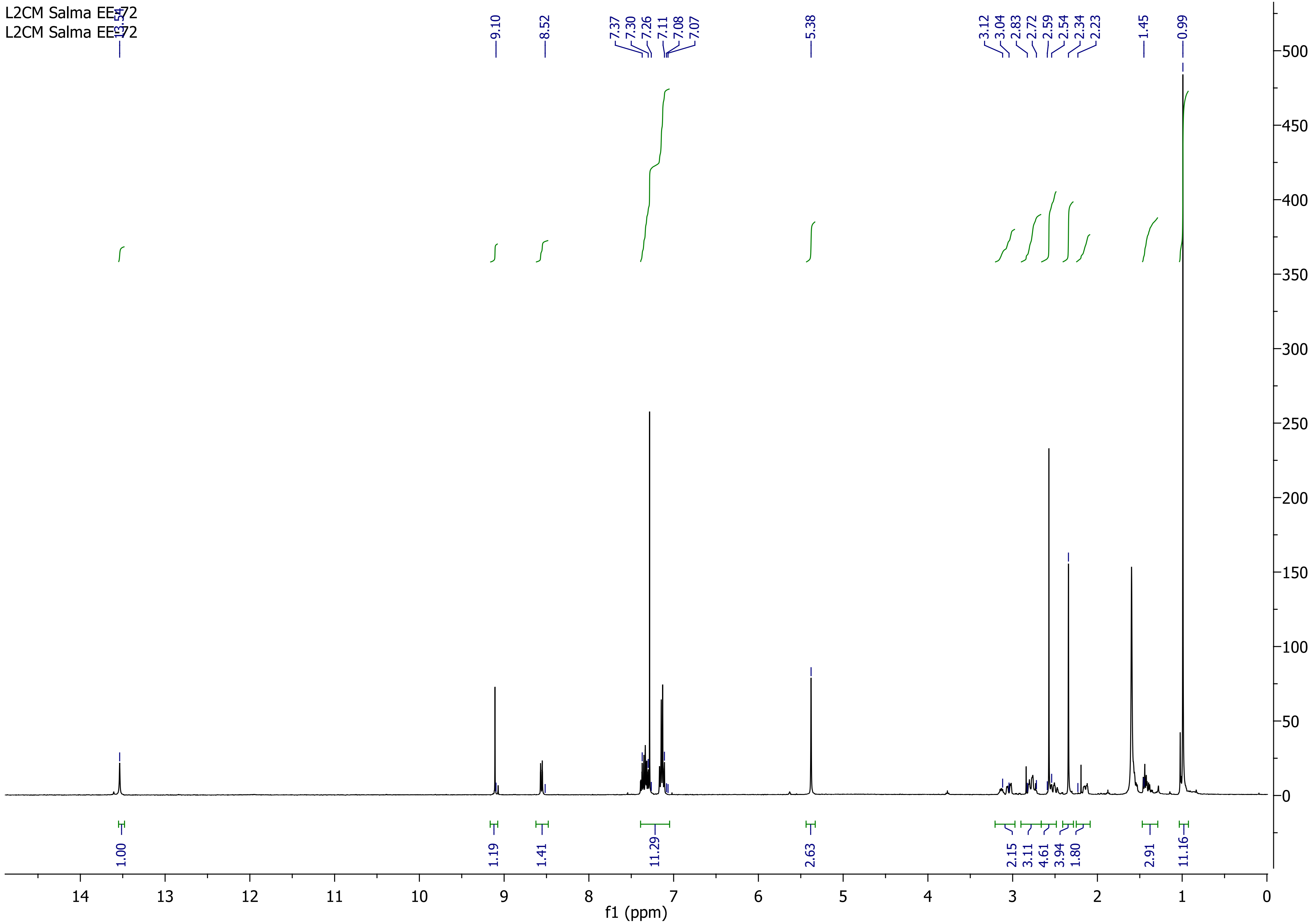

L2CM Salma EE-72
L2CM Salma EE-72
13.54
9.10
8.52
7.37
7.30
7.26
7.11
7.08
7.07
5.38
3.12
3.04
2.83
2.72
2.59
2.54
2.34
2.23
1.45
0.99
1.00
1.19
1.41
11.29
2.63
2.15
3.11
4.61
3.94
1.80
2.91
11.16
f1 (ppm)

L2CM Salma EE-72

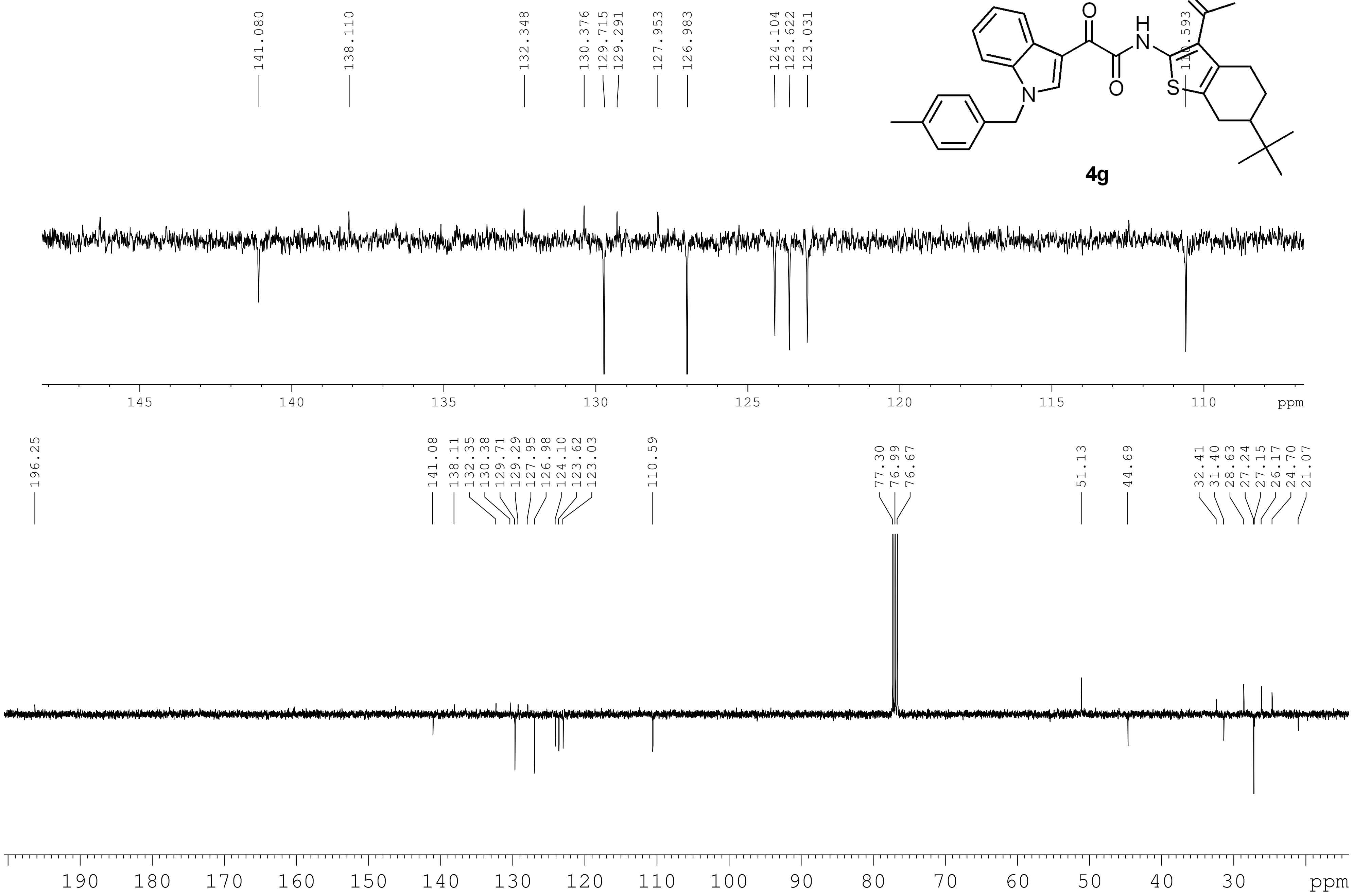

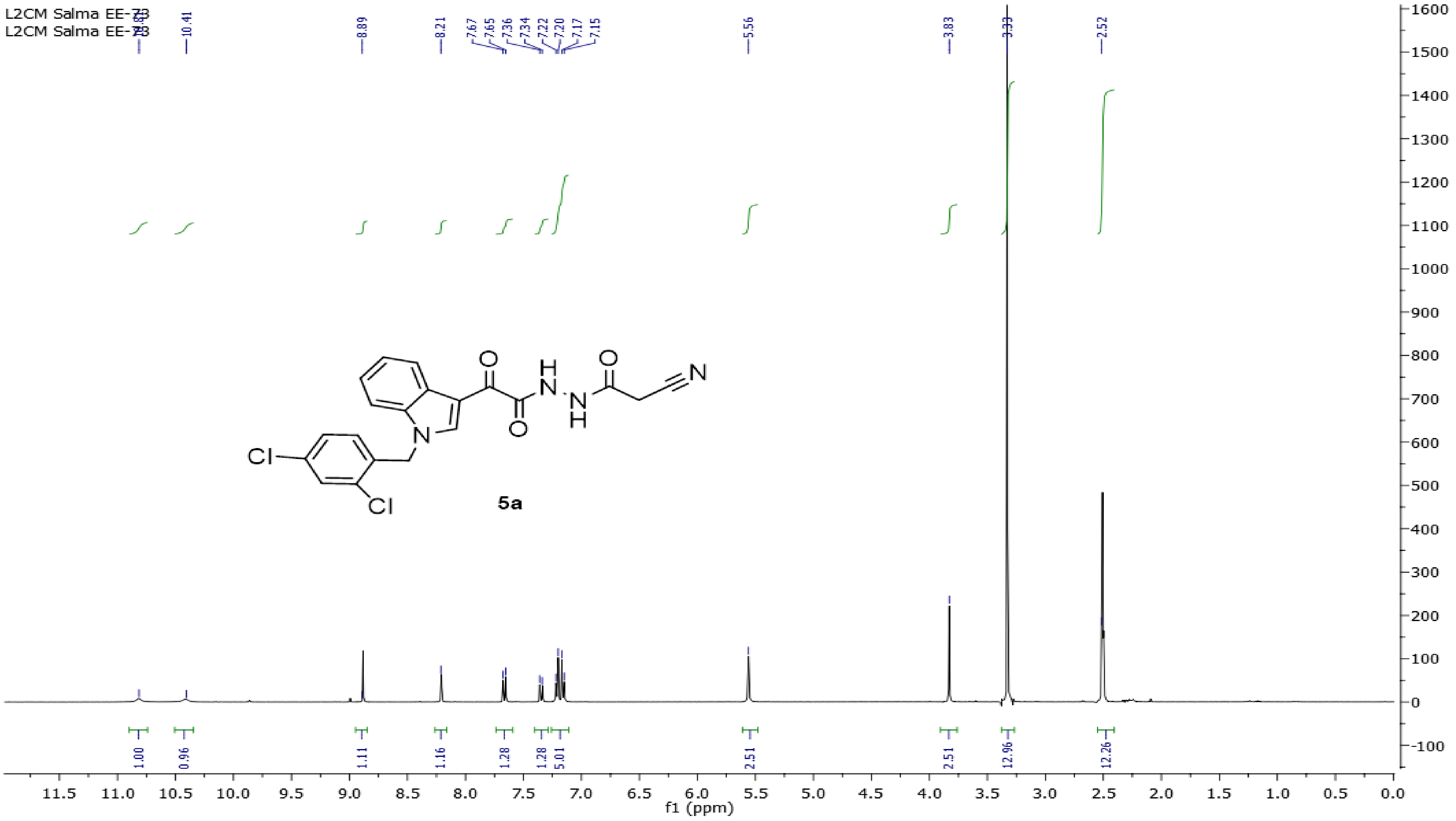
L2CM Salma EE-73
L2CM Salma EE-73
10.81
10.41
8.89
8.21
7.67
7.65
7.36
7.34
7.22
7.20
7.17
7.15
5.56
3.83
3.33
2.52
Cl
Cl
N
O
O
H
N
N
H
O
N
5a
1.00
0.96
1.11
1.16
1.28
1.28
5.01
2.51
2.51
12.96
12.26
f1 (ppm)

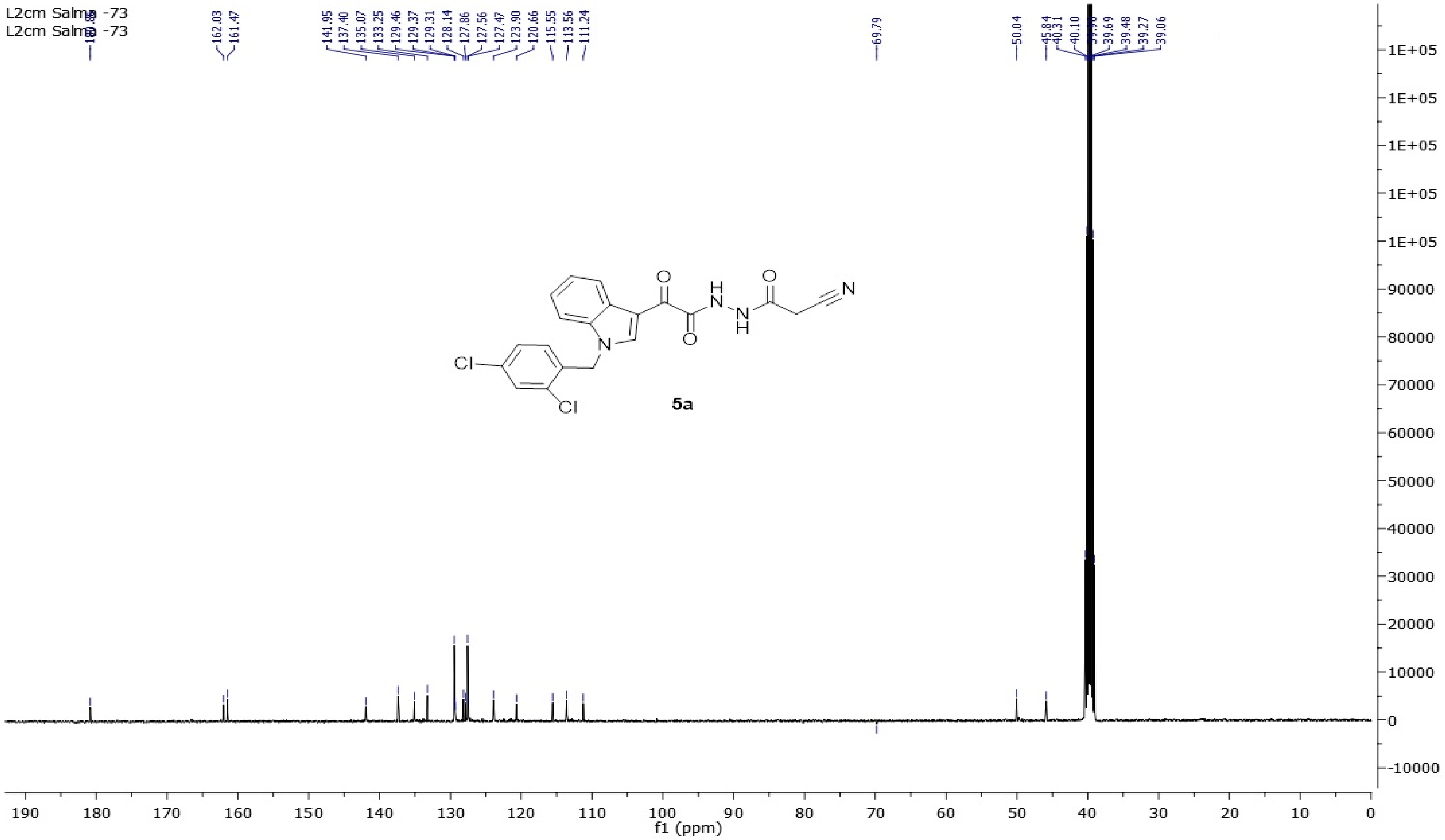

L2cm Salma -73
L2cm Salma -73
162.03
161.47
141.95
137.40
135.07
133.25
129.46
129.37
129.31
128.14
127.86
127.56
127.47
123.90
120.66
115.55
113.56
111.24
69.79
50.04
45.84
40.31
40.10
39.69
39.48
39.27
39.06
5a
Cl
Cl
N
O
H
f1 (ppm)

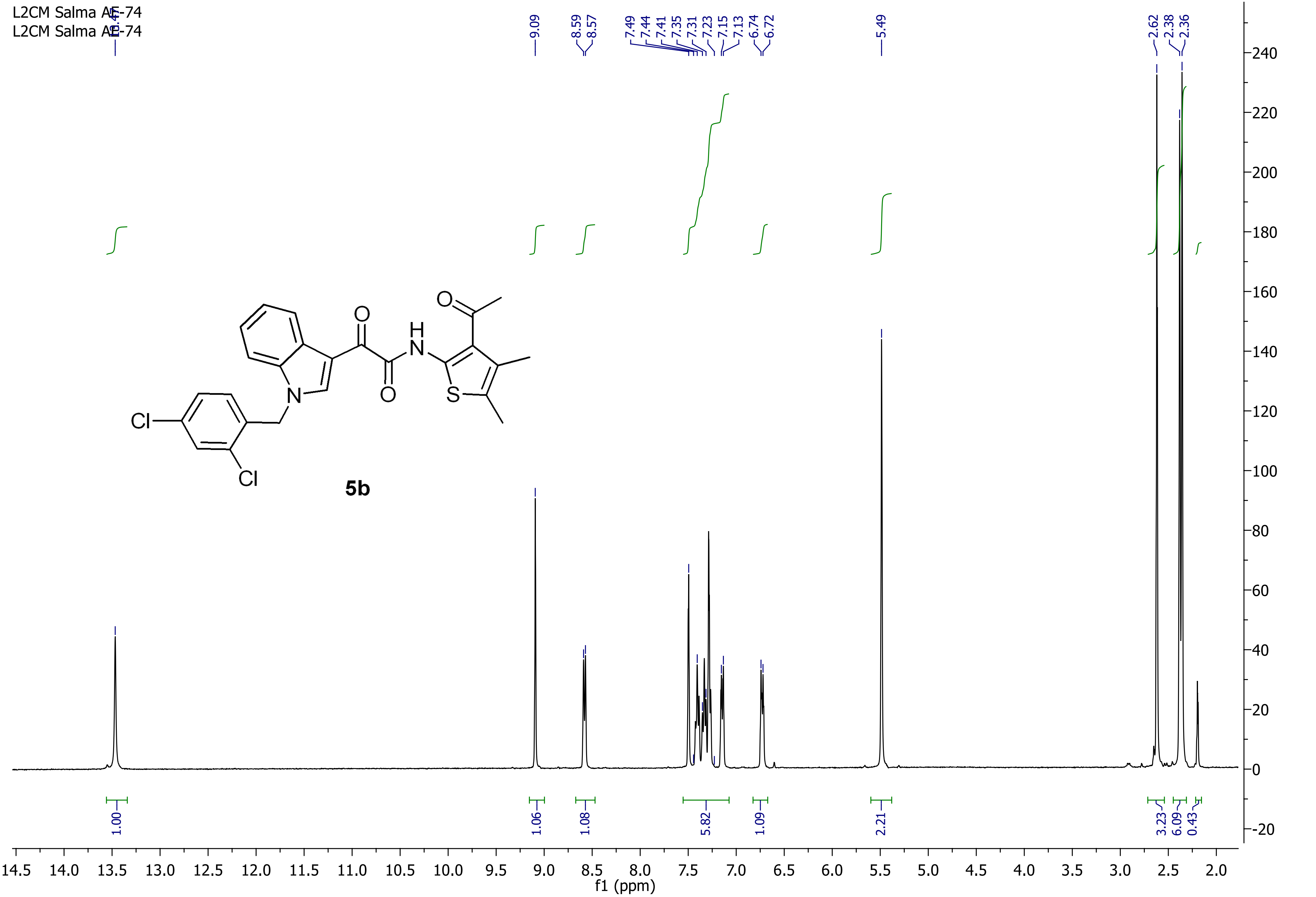
L2CM Salma AE-74
L2CM Salma AE-74
13.47
9.09
8.59
8.57
7.49
7.44
7.41
7.35
7.31
7.23
7.15
7.13
6.74
6.72
5.49
2.62
2.38
2.36
5b
1.00
1.06
1.08
5.82
1.09
2.21
3.23
6.09
0.43
f1 (ppm)

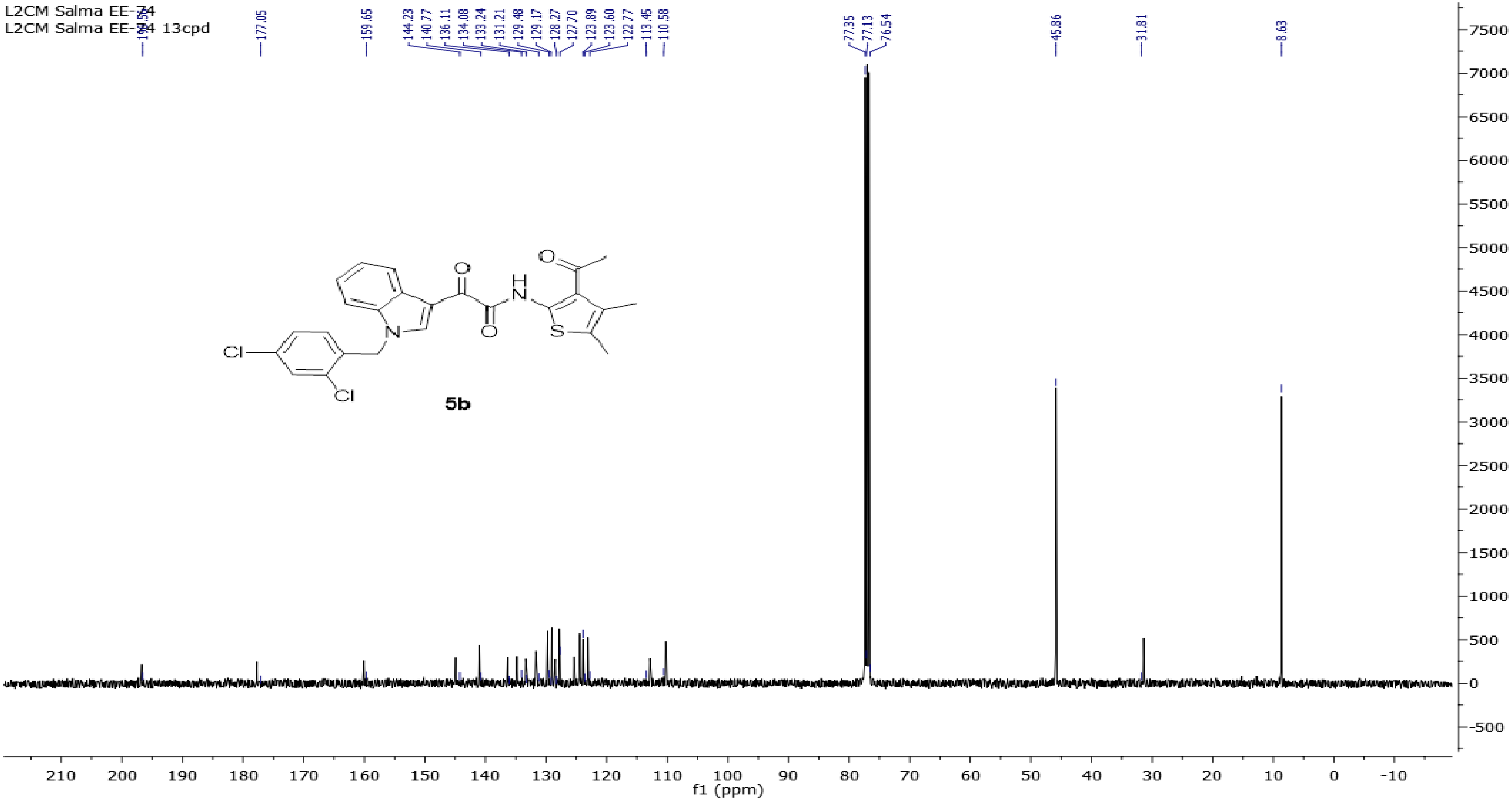
L2CM Salma EE-74
L2CM Salma EE-74 13cpd
177.05
159.65
144.23
140.77
136.11
134.08
133.24
131.21
129.48
129.17
128.27
127.70
123.89
123.60
122.77
113.45
110.58
77.35
77.13
76.54
45.86
31.81
8.63
5b
f1 (ppm)

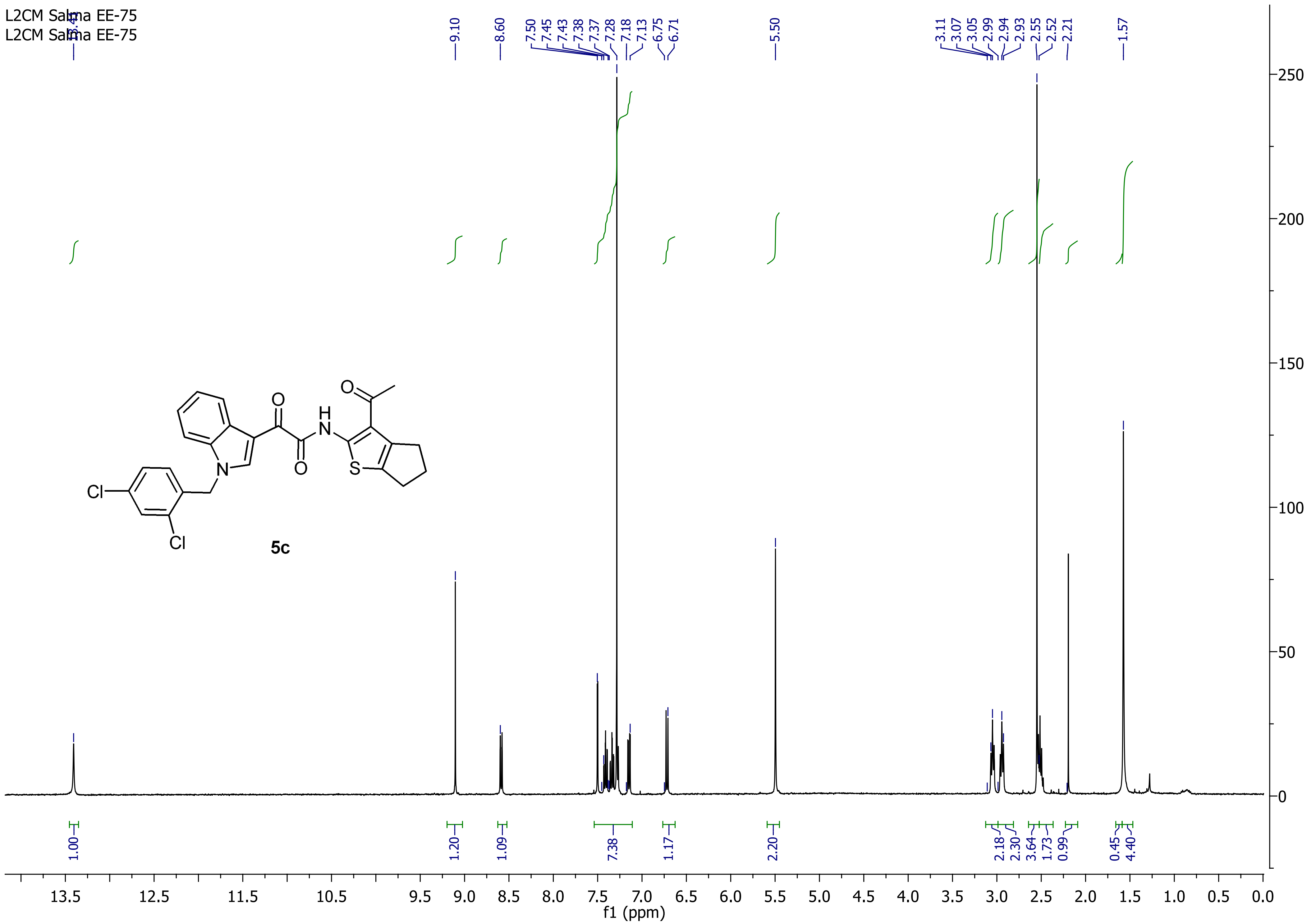

L2CM Salma EE-75
L2CM Salma EE-75
9.10
8.60
7.50
7.45
7.43
7.38
7.37
7.28
7.18
7.13
6.75
6.71
5.50
3.11
3.07
3.05
2.99
2.94
2.93
2.55
2.52
2.21
1.57
1.00
1.20
1.09
7.38
1.17
2.20
2.18
2.30
3.64
1.73
0.99
0.45
4.40
O
O
O
H
N
N
S
Cl
Cl
5c
13.5
12.5
11.5
10.5
9.5
9.0
8.5
8.0
7.5
7.0
6.5
6.0
5.5
5.0
4.5
4.0
3.5
3.0
2.5
2.0
1.5
1.0
0.5
0.0
f1 (ppm)
250
200
150
100
50
0

Mass Spectrum SmartFormula Report

Analysis Info

Analysis Name	D:\Data\2018\180924000005.d	Acquisition Date	9/24/2018 1:42:55 PM
Method	Tuneposlow.m	Operator	fabien
Sample Name	180913809///EE-75	Instrument	micrOTOF-Q 228888.00098
Comment	MeOH/CH2Cl2		

Acquisition Parameter

Source Type	ESI	Ion Polarity	Positive	Set Nebulizer	1.0 Bar
Focus	Not active	Set Capillary	4500 V	Set Dry Heater	190 °C
Scan Begin	50 m/z	Set End Plate Offset	-500 V	Set Dry Gas	4.0 l/min
Scan End	3000 m/z	Set Collision Cell RF	250.0 Vpp	Set Divert Valve	Source

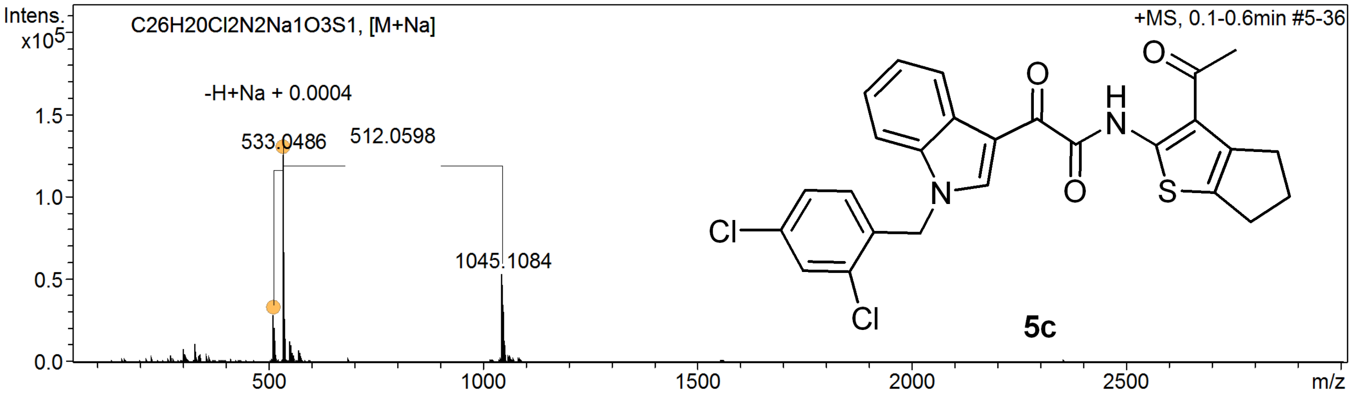

Meas. m/z	#	Ion Formula	m/z	|err| [mDa]	err [ppm]	mSigma	# mSigma	Score	rdb	e⁻ Conf	N-Rule
511.0662	1	C26H21Cl2N2O3S	511.0644	1.8	-3.4	3.2	1	100.00	16.5	even	ok
533.0486	1	C26H20Cl2N2NaO3S	533.0464	2.2	-4.1	0.7	1	100.00	16.5	even	ok

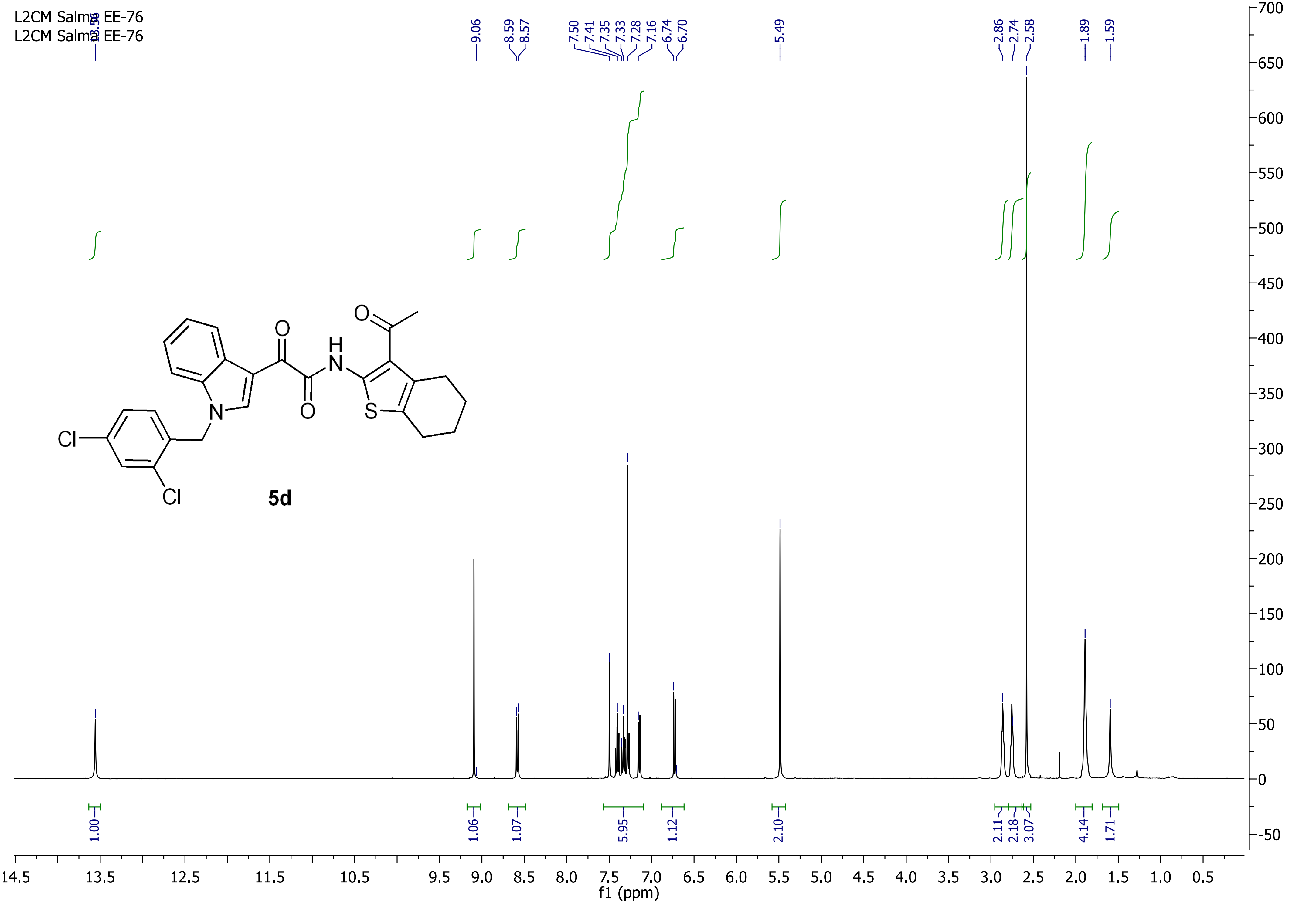
L2CM Salma EE-76
L2CM Salma EE-76
13.58
9.06
8.59
8.57
7.50
7.41
7.35
7.33
7.28
7.16
6.74
6.70
5.49
2.86
2.74
2.58
1.89
1.59
1.00
1.06
1.07
5.95
1.12
2.10
2.11
2.18
3.07
4.14
1.71
O
O
H
N
O
N
S
Cl
Cl
5d
f1 (ppm)

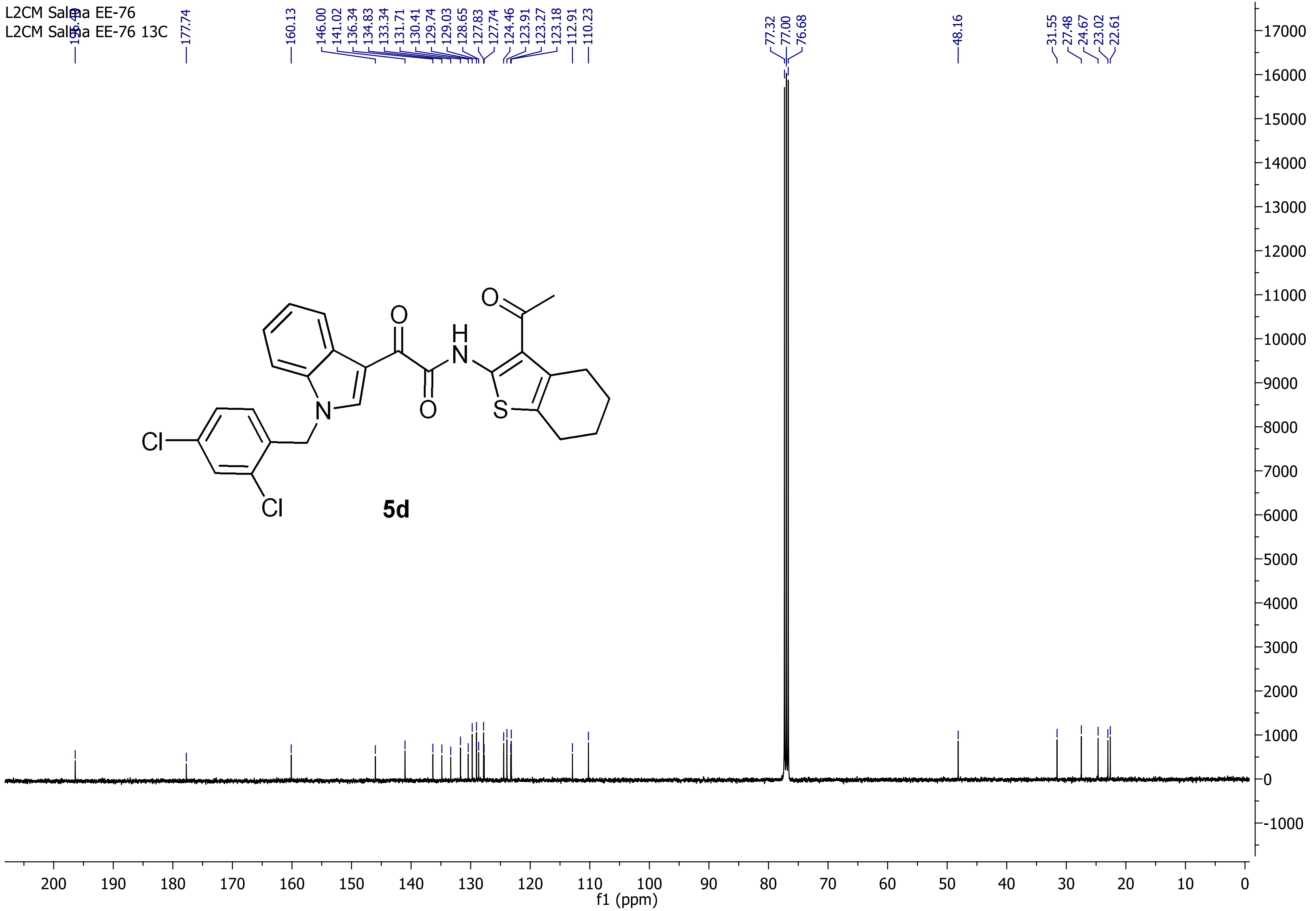

L2CM Salma EE-76
L2CM Salma EE-76 13C
196.40
177.74
160.13
146.00
141.02
136.34
134.83
133.34
131.71
130.41
129.74
129.03
128.65
127.83
127.74
124.46
123.91
123.27
123.18
112.91
110.23
77.32
77.00
76.68
48.16
31.55
27.48
24.67
23.02
22.61
O
H
N
O
O
S
N
Cl
Cl
5d
17000
16000
15000
14000
13000
12000
11000
10000
9000
8000
7000
6000
5000
4000
3000
2000
1000
0
-1000
200
190
180
170
160
150
140
130
120
110
100
90
80
70
60
50
40
30
20
10
0
f1 (ppm)

Mass Spectrum SmartFormula Report

Analysis Info

Analysis Name	D:\Data\2018\180924000006.d	Acquisition Date	9/24/2018 1:48:49 PM
Method	Tuneposlow.m	Operator	fabien
Sample Name	180913810///EE-76	Instrument	micrOTOF-Q 228888.00098
Comment	MeOH/CH2Cl2		

Acquisition Parameter

Source Type	ESI	Ion Polarity	Positive	Set Nebulizer	1.0 Bar
Focus	Not active	Set Capillary	4500 V	Set Dry Heater	190 °C
Scan Begin	50 m/z	Set End Plate Offset	-500 V	Set Dry Gas	4.0 l/min
Scan End	3000 m/z	Set Collision Cell RF	250.0 Vpp	Set Divert Valve	Source

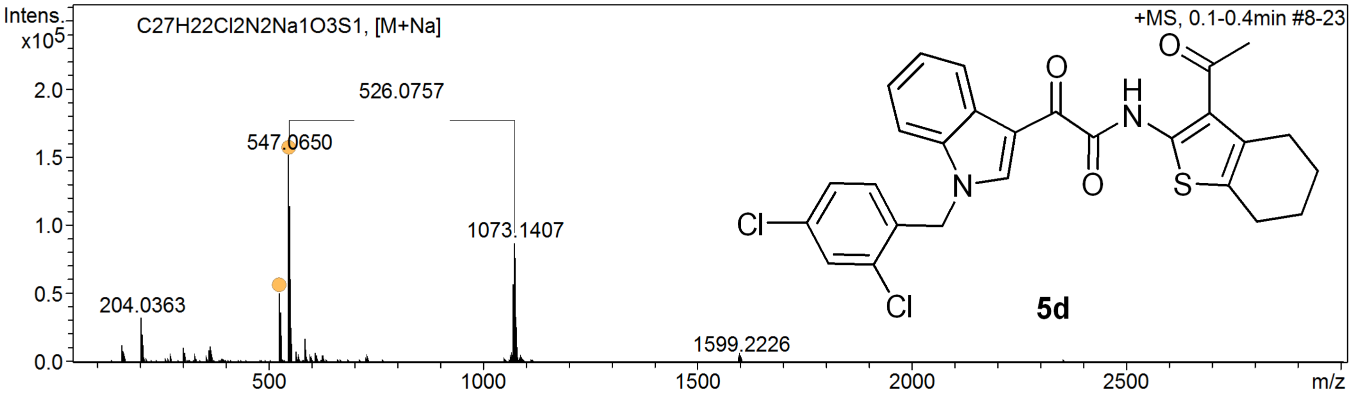

Meas. m/z	#	Ion Formula	m/z	|err| [mDa]	err [ppm]	mSigma	# mSigma	Score	rdb	e⁻ Conf	N-Rule
525.0828	1	C27H23Cl2N2O3S	525.0801	2.7	-5.2	6.8	1	100.00	16.5	even	ok
547.0650	1	C27H22Cl2N2NaO3S	547.0620	3.0	-5.5	7.5	1	100.00	16.5	even	ok

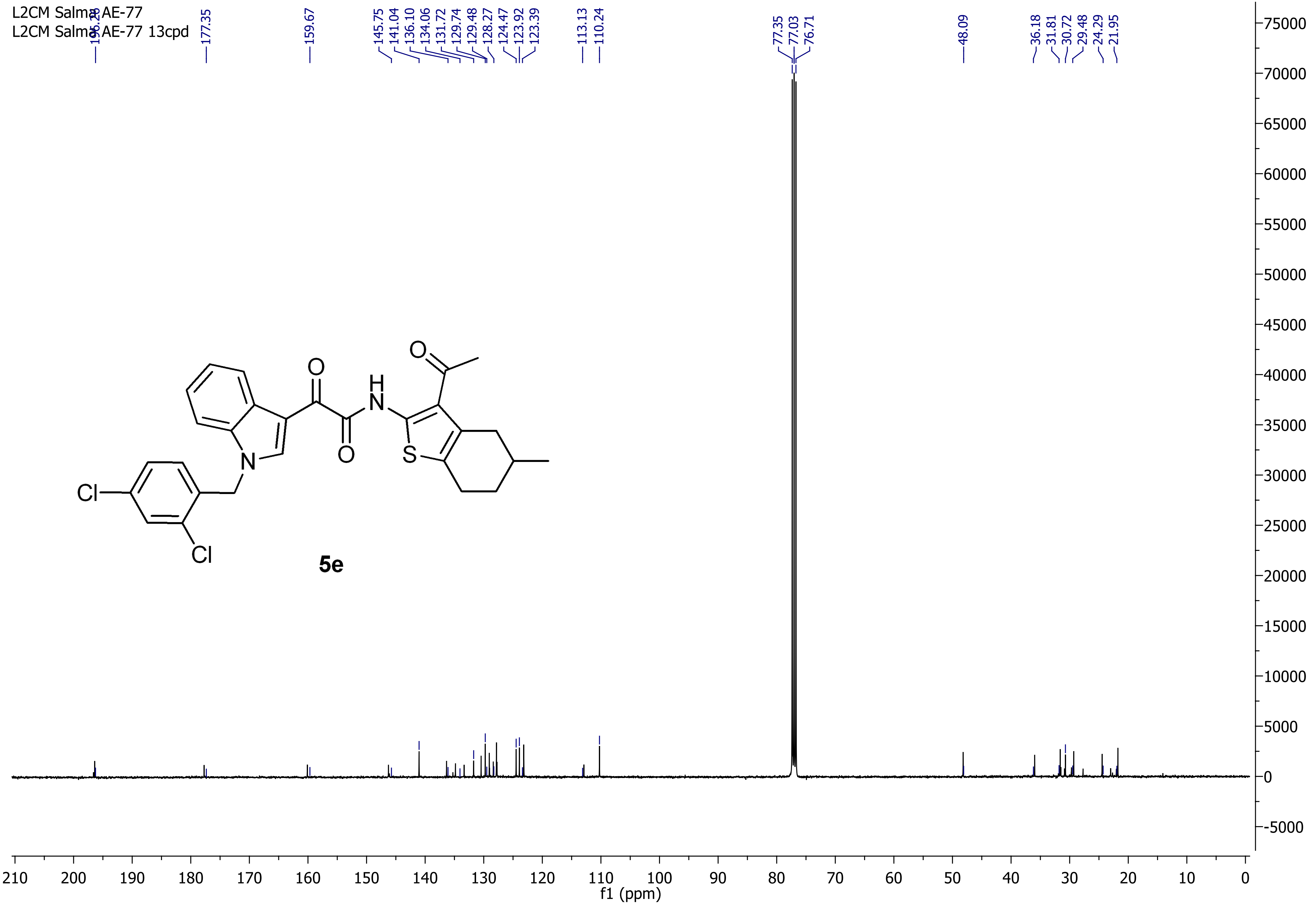
L2CM Salma AE-77
L2CM Salma AE-77 13cpd
196.28
177.35
159.67
145.75
141.04
136.10
134.06
131.72
129.74
129.48
128.27
124.47
123.92
123.39
113.13
110.24
77.35
77.03
76.71
48.09
36.18
31.81
30.72
29.48
24.29
21.95
O
O
H
N
O
N
S
Cl
Cl
5e
75000
70000
65000
60000
55000
50000
45000
40000
35000
30000
25000
20000
15000
10000
5000
0
-5000
210
200
190
180
170
160
150
140
130
120
110
100
90
80
70
60
50
40
30
20
10
0
f1 (ppm)

L2CM Salma EE-77
L2CM Salma EE-77

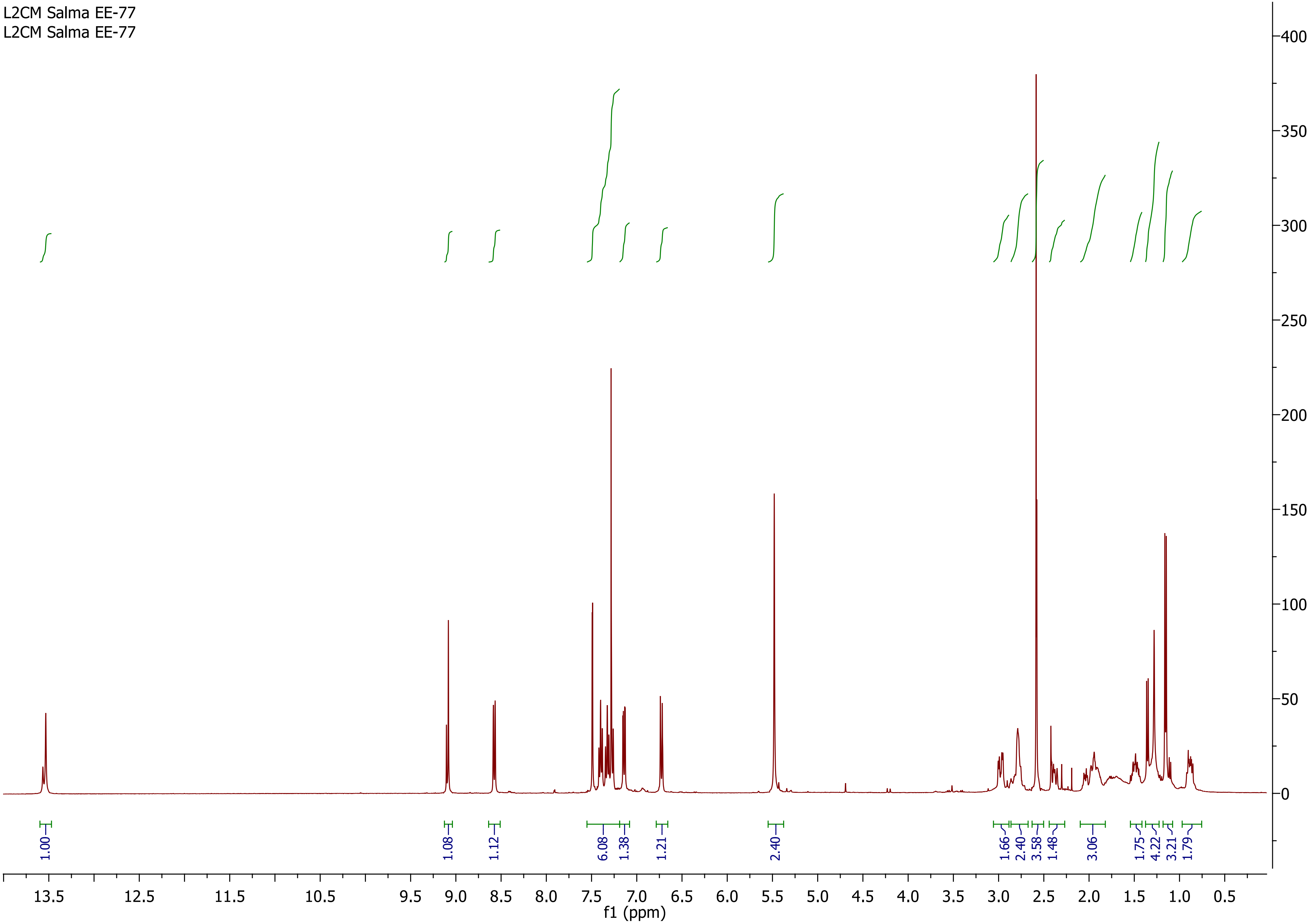

L2CM Salma AE-78

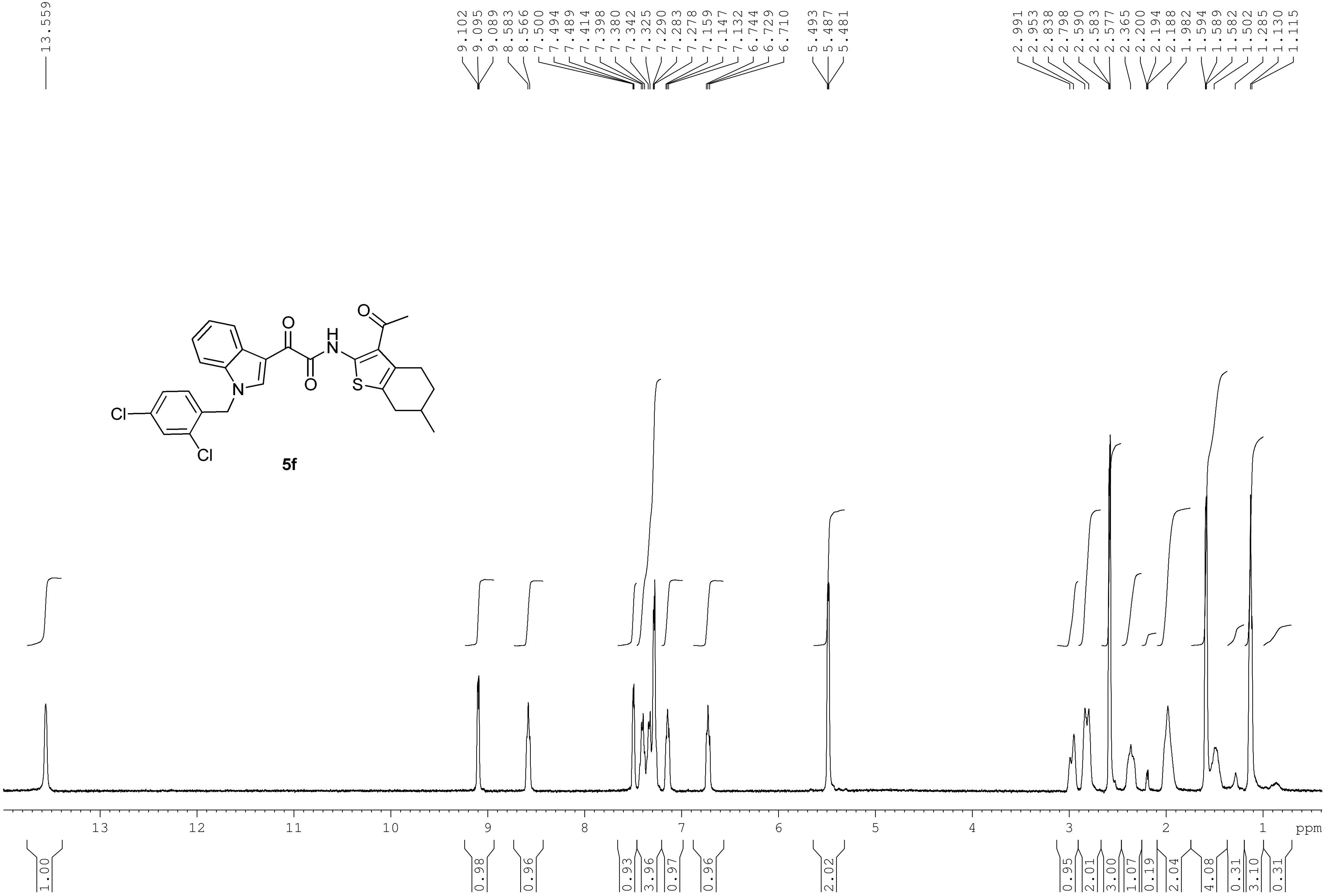

5f

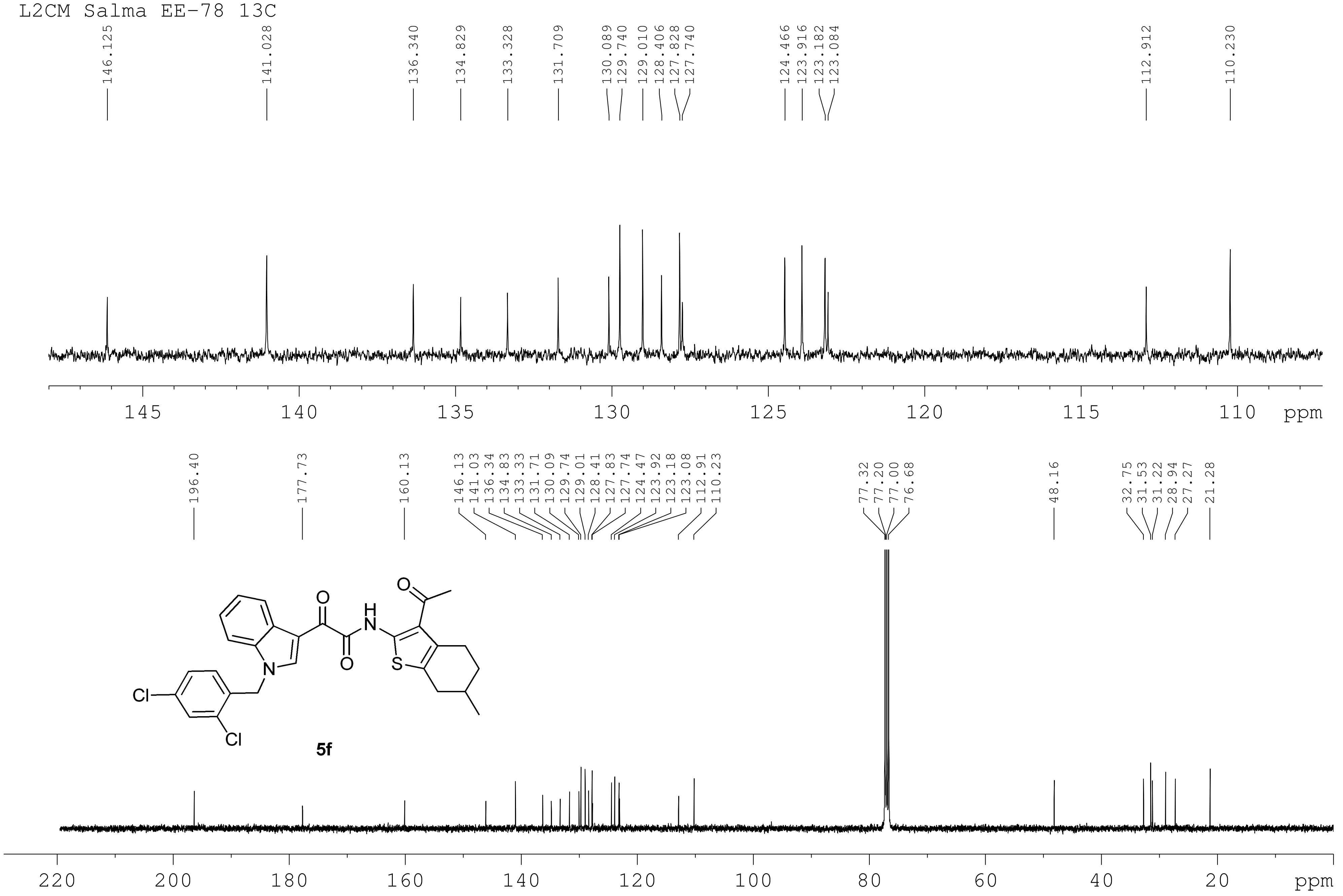
L2CM Salma EE-78 13C
146.125
141.028
136.340
134.829
133.328
131.709
130.089
129.740
129.010
128.406
127.828
127.740
124.466
123.916
123.182
123.084
112.912
110.230
145 140 135 130 125 120 115 110 ppm
196.40
177.73
160.13
146.13
141.03
136.34
134.83
133.33
131.71
130.09
129.74
129.01
128.41
127.83
127.74
124.47
123.92
123.18
123.08
112.91
110.23
77.32
77.20
77.00
76.68
48.16
32.75
31.53
31.22
28.94
27.27
21.28
O
H
N
S
Cl
Cl
5f
220 200 180 160 140 120 100 80 60 40 20 ppm

Mass Spectrum SmartFormula Report

Analysis Info

Analysis Name	D:\Data\2018\180924000007.d	Acquisition Date	9/24/2018 1:55:36 PM
Method	Tuneposlow.m	Operator	fabien
Sample Name	180913811///EE-78	Instrument	micrOTOF-Q 228888.00098
Comment	MeOH/CH2Cl2		

Acquisition Parameter

Source Type	ESI	Ion Polarity	Positive	Set Nebulizer	1.0 Bar
Focus	Not active	Set Capillary	4500 V	Set Dry Heater	190 °C
Scan Begin	50 m/z	Set End Plate Offset	-500 V	Set Dry Gas	4.0 l/min
Scan End	3000 m/z	Set Collision Cell RF	250.0 Vpp	Set Divert Valve	Source

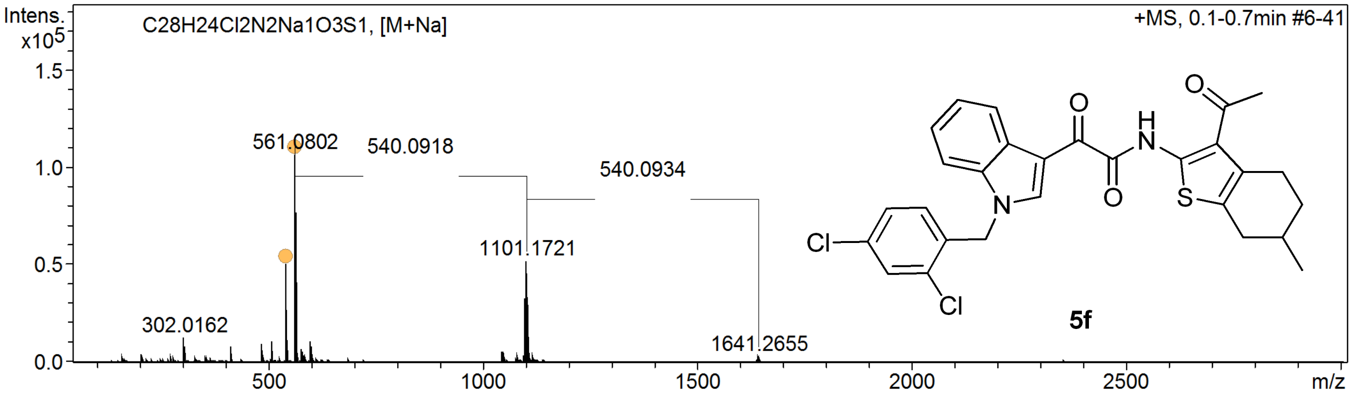

Meas. m/z	#	Ion Formula	m/z	|err| [mDa]	err [ppm]	mSigma	# mSigma	Score	rdb	e⁻ Conf	N-Rule
539.0979	1	C28H25Cl2N2O3S	539.0957	2.2	-4.1	13.3	1	100.00	16.5	even	ok
561.0802	1	C28H24Cl2N2NaO3S	561.0777	2.5	-4.5	8.7	1	100.00	16.5	even	ok

L2CM Salma EE-79
L2CM Salma EE-79

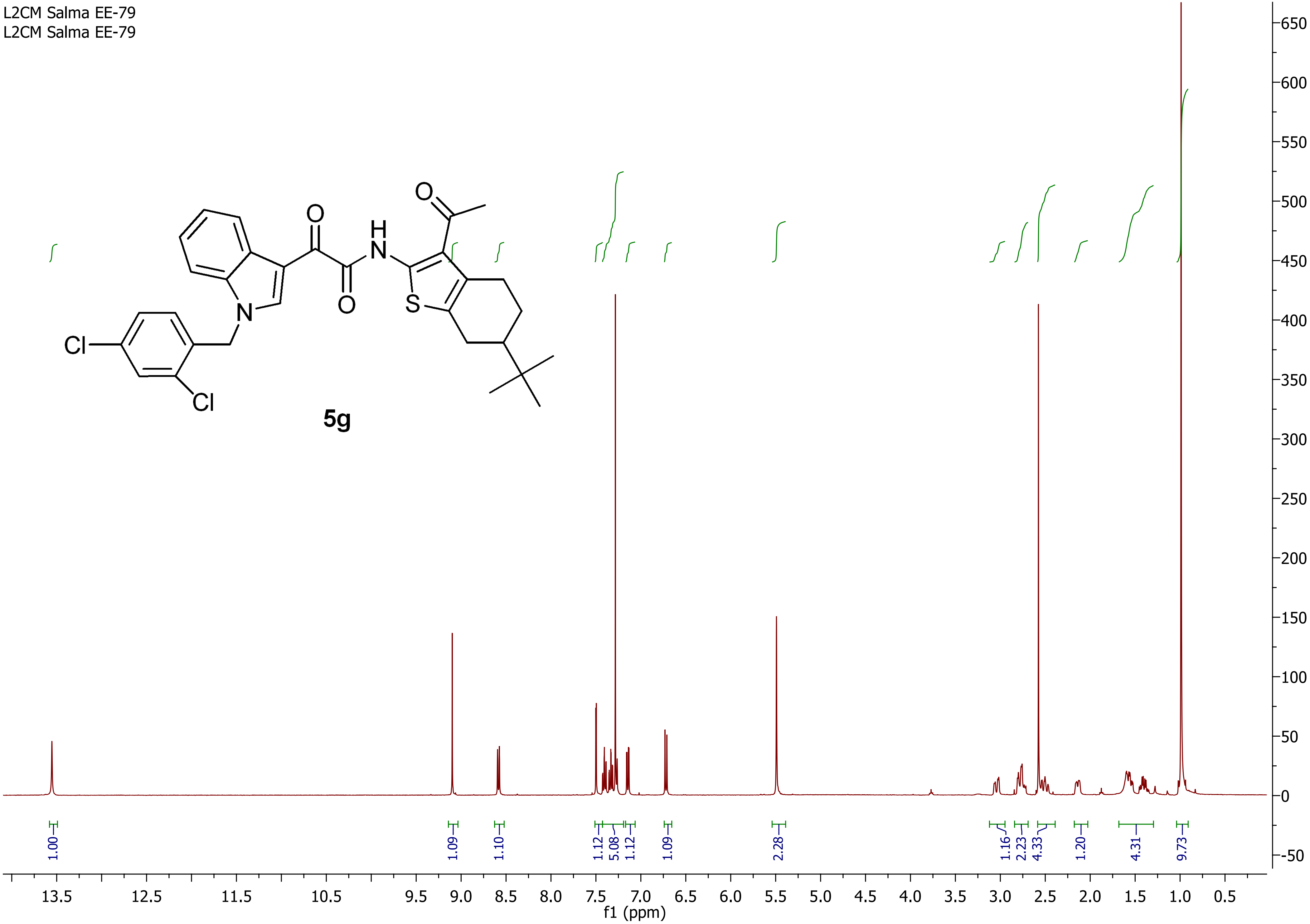

L2cm salma EE-79

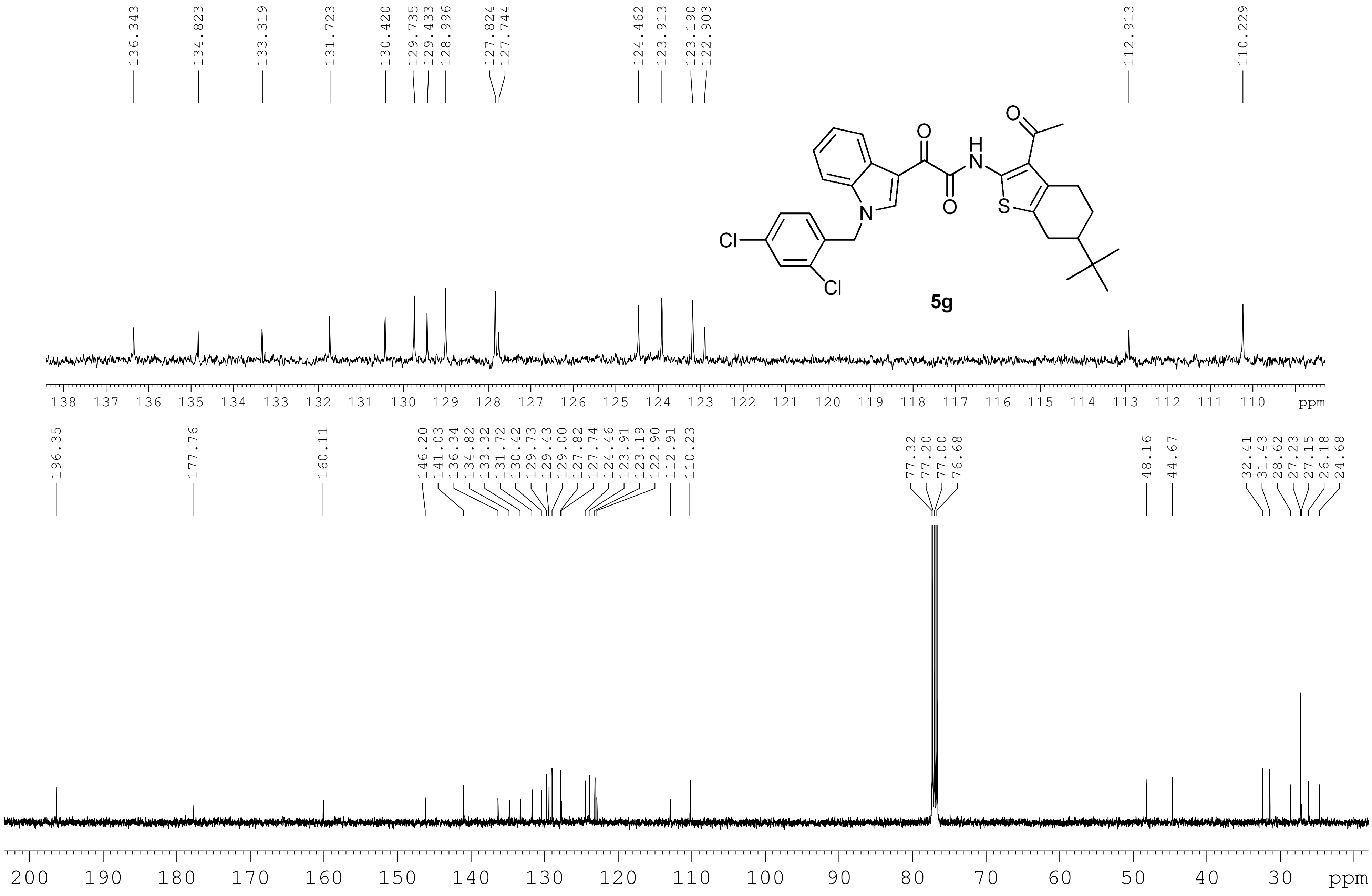

Mass Spectrum SmartFormula Report

Analysis Info

Analysis Name	D:\Data\2018\180924000008.d	Acquisition Date	9/24/2018 2:20:41 PM
Method	Tuneposlow.m	Operator	fabien
Sample Name	180913812///EE-79	Instrument	micrOTOF-Q 228888.00098
Comment	MeOH/CH2Cl2		

Acquisition Parameter

Source Type	ESI	Ion Polarity	Positive	Set Nebulizer	1.0 Bar
Focus	Not active	Set Capillary	4500 V	Set Dry Heater	190 °C
Scan Begin	50 m/z	Set End Plate Offset	-500 V	Set Dry Gas	4.0 l/min
Scan End	3000 m/z	Set Collision Cell RF	250.0 Vpp	Set Divert Valve	Source

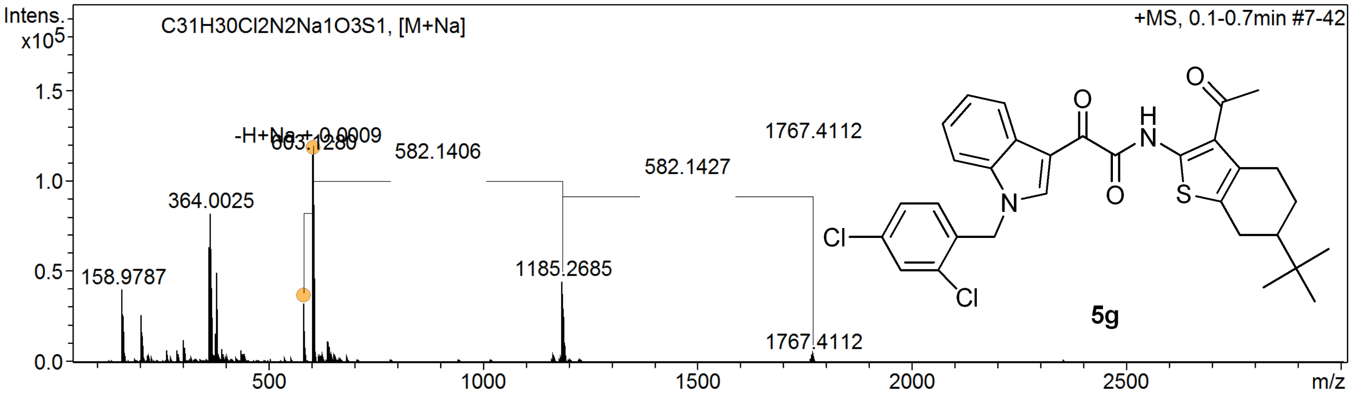

5g

Meas. m/z	#	Ion Formula	m/z	|err| [mDa]	err [ppm]	mSigma	# mSigma	Score	rdb	e⁻ Conf	N-Rule
581.1451	1	C31H31Cl2N2O3S	581.1427	2.4	-4.2	10.9	1	100.00	16.5	even	ok
603.1280	1	C31H30Cl2N2NaO3S	603.1246	3.3	-5.5	3.2	1	100.00	16.5	even	ok
