# Supplementary material for: Synthetic Analogs of Marine Alkaloid Aplysinopsin Suppress Anti-Apoptotic Protein BCL2 in Prostate Cancer
Source: Molecules. 2022 Dec 23;28(1):109. doi: 10.3390/molecules28010109 (PMC9821956; doi:10.3390/molecules28010109)
Supplement: Supplementary file 1 [file molecules-28-00109-s001.zip › Supporting information.pdf]

Synthetic analogs of marine alkaloid aplysinopsin suppress anti-apoptotic protein BCL2 in prostate cancer

Eslam R. El-Sawy[1,*], Zeinab A. El-Shahid[2], Ahmed A.F. Soliman[3], Amr Nassrallah[4], Ahmed B. Abdelwahab[5], Gilbert Kirsch[6,*] and Heba Abdelmegeed[1,*]

[1] Chemistry of Natural Compounds Department, National Research Centre, 12622 Giza, Egypt
[2] Chemistry of Natural and Microbial Products Department, National Research Centre, 12622 Giza, Egypt
[3] Drug Bioassay-Cell Culture Laboratory, Pharmacognosy Department, National Research Center, 12622Dokki, Giza, Egypt
[4] Biochemistry Department, Faculty of Agriculture, Cairo University, 12613 Giza, Egypt
[5] Plant Advanced Technologies (PAT), Vandœuvre-lès-Nancy 54500, France
[6] Laboratoire Lorrain de Chimie Moléculaire (L.2.C.M.), Université de Lorraine, 57050 Metz, France
*Correspondence: eslamelsawy@gmail.com, heba.abdelmegeed.eldeeb@gmail.com, and gilbert.kirsch@univ-lorraine.fr ; Tel.: (+33) 03.72.74.92.00; Fax: (+33) 03.72.74.91.87).

Materials and Methods

Chemistry

General Information

All reagents and solvents were of commercial grade. Melting points were determined on the digital melting point apparatus (Electro thermal 9100, Electro thermal Engineering Ltd., serial No. 8694, Rochford, UK) and are uncorrected. Elemental analyses were performed on a Flash Smart™ Elemental Analyzer (Thermo Scientific, Courtaboeuf, France) and were found within ± 0.4% of the theoretical values. ^{1}H and ^{13}C NMR spectra were measured with a Bruker Avance spectrometer (Bruker, Germany) at 400 and 101 MHz, respectively, using TMS as the internal standard. Hydrogen coupling patterns are described as (s) singlet, (d) doublet, (t) triplet, (q) quartet, and (m) multiplet. The chemical shifts were defined as parts per million (ppm) relative to the solvent peak. The reaction progress was checked by pre-coated TLC Silica gel 0.2 nm F254 nm [Fluka], visualized under UV lamp 254 and 365 nm. Cyanoacetic acide hydrazide [21], N-benzyl indoles [22]; methyl creatinine [23]; indole-3-aldehyde [24] were prepared as reported.

General procedure for the preparation of aplysinopsins analogs

A solution of oxalyl chloride (0.44 ml, 5.1 mmol) in dry ethyl ether (25 ml) was treated with a solution of indoles (4.14 mmol) in dry ethyl ether (5 ml) dropwise under cooling. The resulting yellow slurry was refluxed for 2 h. After removing the ether under vacuum, the remains were dissolved in dry tetrahydrofuran (20 ml), and then cooled to 0°C. A solution of amines (9.73 mmol) in dry tetrahydrofuran (20 ml) was added slowly to the THF solutions under stirring. After complete addition, 1 ml of triethylamine was added to the reaction mixture and left to stir overnight. The formed precipitate was filtered off, washed several times with water, dried, and recrystallized from acetone.

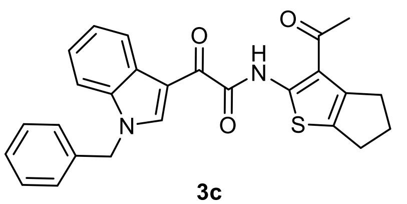

3c

N-(3-Acetyl-5,6-dihydro-4*H*-cyclopenta[*b*]thiophen-2-yl)-2-(1-benzyl-1*H*-indol-3-yl)-2-oxo-acetamide (**3c, EE-65**). Yield (0.35g, 55%); mp 240-2°C; **^{1}H-NMR** (400 MHz, $CDCl_3$) δ 13.38 (s, 1H), 9.11 (s, 1H), 8.57 (dt, *J* = 7.9, 1.0 Hz, 1H), 8.04 (s, 1H), 7.43 – 7.15 (m, 7H), 5.42 (s, 2H), 3.19 – 2.81 (m, 6H), 2.58 (s, 3H); **^{13}C-NMR** (101 MHz, $CDCl_3$) δ 195.56, 177.42, 162.51, 160.16, 150.37, 141.15, 141.01, 136.55, 135.41, 134.21, 129.06, 128.28, 127.89, 126.92, 124.19, 123.68, 123.04, 119.03, 112.51, 110.58, 51.29, 36.44, 31.42, 31.35, 29.99, 28.73, 28.11; **HRMS**-(ESI): *m/z* $[M+Na]^+$ calcd for $C_{26}H_{22}N_2O_3SNa$ (465.12); Anal calcd for $C_{26}H_{22}N_2O_3S$ (442.53): C, 70.57; H, 5.01; N, 6.33; S, 7.24; found: C, 70.44; H, 5.03; N, 6.22; S, 7.11.

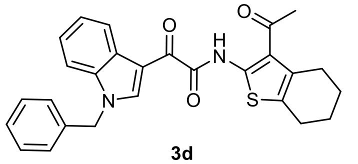

3d

N-(3-Acetyl-4,5,6,7-tetrahydrobenzo[*b*]thiophen-2-yl)-2-(1-benzyl-1*H*-indol-3-yl)-2-oxo-acetamide (**EE-45, 3d**). Yield (0.42g, 64%); mp 214-6°C; **^{1}H-NMR** (400 MHz, DMSO-d_6) δ 13.28 (s, 1H), 9.25 (s, 1H), 8.47 – 8.14 (m, 1H), 7.81 – 7.51 (m, 1H), 7.46 – 6.92 (m, 7H), 5.66 (s, 2H), 2.84 (d, *J* = 15.8 Hz, 2H), 2.63 (d, *J* = 15.8 Hz, 2H), 2.52 (s, 3H), 1.78 (s, 4H); **^{13}C- NMR** (101 MHz, DMSO) δ 196.76, 177.21, 159.56, 144.82, 141.87, 136.47, 136.25, 130.70, 128.75, 127.85, 127.69, 127.44, 127.11, 123.96, 123.44, 122.75, 121.73, 111.73, 111.11, 49.96, 31.55, 26.45, 24.07, 22.47, 22.12; **HRMS**-(ESI): *m/z* $[M+Na]^+$ calcd for $C_{27}H_{24}N_2O_3SNa$ (479.14); Anal calcd for $C_{27}H_{24}N_2O_3S$ (456.56): C, 71.03; H, 5.30; N, 6.14; S, 7.02; found: C, 71.21; H, 5.01; N, 6.22; S, 7.10.

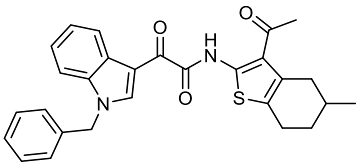

3e

N-(3-Acetyl-5-methyl-4,5,6,7-tetrahydrobenzo[*b*]thiophen-2-yl)-2-(1-benzyl-1*H*-indol-3-yl)-2-oxo-acetamide (**EE-47,3e**). Yield (0.61g, 90%), mp 206-8°C; **^{1}H-NMR** (400 MHz, $CDCl_3$) δ 13.54 (s, 1H), 9.14 (s, 1H), 8.57 (d, *J* = 7.9 Hz, 1H), 7.30 (ddd, *J* = 28.9, 21.4, 8.6 Hz, 7H), 5.41 (s, 2H), 2.97 (dd, *J* = 34.9, 55.5 Hz, 1H), 2.86-2.80 (m, 1H), 2.51(s, 3H), 2.51 – 1.99 (m, 1H), 2.03 (d, *J* = 15.8 Hz, 1H), 2.01 (d, *J* = 15.8 Hz, 2H), 1.82 – 1.78 (m, 2H), 0.90 (s, 3H); **^{13}C-NMR** (101 MHz, $CDCl_3$) δ 196.35, 177.58, 160.31, 146.32, 141.13, 136.55, 135.41, 130.42, 129.07, 128.29, 127.91, 126.94, 124.18, 123.68, 123.12, 123.05, 112.55, 110.56, 51.30, 35.97, 31.59, 30.73, 29.31, 24.44, 21.77; **HRMS**-(ESI): *m/z* $[M+Na]^+$ calcd for $C_{28}H_{26}N_2O_3SNa$ (493.15); Anal calcd for $C_{28}H_{26}N_2O_3S$ (470.59): C, 71.47; H, 5.57; N, 5.95; S, 6.81; found: C, 71.32; H, 5.55; N, 6.00; S, 6.79.

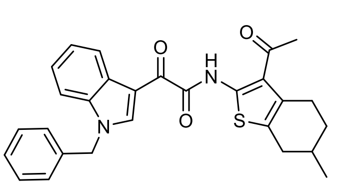

3f

N-(3-Acetyl-6-methyl-4,5,6,7-tetrahydrobenzo[*b*]thiophen-2-yl)-2-(1-benzyl-1*H*-indol-3-yl)-2-oxo-acetamide (**EE-46, 3f**). Yield (0.36g, 53%), mp 210-2°C; **^{1}H-NMR** (400 MHz, $CDCl_3$) δ 13.54 (s, 1H), 9.13 (s, 1H), 8.57 (d, *J* = 7.8 Hz, 1H), 7.43 – 7.18 (m, 7H), 5.42 (s, 2H), 2.98 (dd, *J* = 15.9, 5.0 Hz, 1H), 2.84 – 2.72 (m, 1H), 2.58 (s, 3H), 2.41 – 2.27 (m, 1H), 2.19 (s, 1H), 2.08 – 1.87 (m, 1H), 1.58 (d, *J* = 28.1 Hz, 1H), 1.51 – 1.24 (m, 2H), 1.14 (s, 3H); Anal calcd for $C_{28}H_{26}N_2O_3S$ (470.59): C, 71.47; H, 5.57; N, 5.95; S, 6.81; found: C, 71.32; H, 5.61; N, 6.01; S, 6.51.

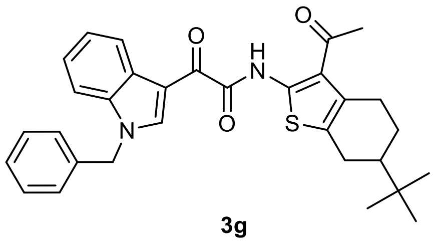

3g

N-(3-Acetyl-6-(tert-butyl)-4,5,6,7-tetrahydrobenzo[*b*]thiophen-2-yl)-2-(1-benzyl-1*H*-indol-3-yl)-2-oxo-acetamide (**EE-43, 3g**). Yield (0.35g, 47%); mp 115-7°C; **^{1}H-NMR** (400 MHz, $CDCl_3$) δ 13.55 (s, 1H), 9.13 (s, 1H), 8.57 (d, *J* = 7.8 Hz, 1H), 7.41 – 7.17 (m, 8H), 5.41 (s, 2H), 3.04 (dd, *J* = 16.0, 4.7 Hz, 2H), 2.84 – 2.73 (m, 2H), 2.57 (s, 3H), 1.62 – 1.50 (m, 2H), 1.45 – 1.34 (m, 1H), 0.98 (s, 9H); **^{13}C-NMR** (101 MHz, $CDCl_3$) δ 196.29, 177.60, 160.30, 146.27, 141.14, 136.55, 135.42, 130.40, 129.32, 129.07, 129.03, 128.28, 128.22, 127.91, 126.93, 125.29, 124.17, 123.67, 123.05, 122.86, 112.54, 110.57, 51.29, 47.02, 44.67, 32.42, 31.43, 28.62, 27.24, 26.17, 24.69, 21.43; **HRMS**-(ESI): *m/z* $[M+Na]^+$ calcd for $C_{31}H_{32}N_2O_3SNa$ (535.20); Anal calcd for: $C_{31}H_{32}N_2O_3S$ (512.67): C, 72.63; H, 6.29; N, 5.46; S, 6.25; found: C, 72.59; H, 6.31; N, 5.50; S, 6.29.

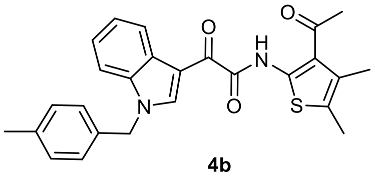

4b

N-(3-Acetyl-4,5-dimethylthiophen-2-yl)-2-(1-(4-methylbenzyl)-1*H*-indol-3-yl)-2-oxo-acetamide (**EE-67, 4b**). Yield (0.28g, 47%); mp 201-3°C; **^{1}H-NMR** (400 MHz, $CDCl_3$) δ 13.46 (s, 1H), 9.11 (s, 1H), 8.56 (d, *J* = 7.8 Hz, 1H), 7.46 – 7.05 (m, 7H), 5.37 (s, 2H), 2.63 (s, 3H), 2.43 – 2.27 (m, 6H), 1.58 (s, 3H); ^{13}C-NMR (101 MHz, $CDCl_3$) δ 145.02, 141.09, 138.12, 136.55, 132.33, 129.72, 127.93, 126.99, 125.21, 124.42, 124.12, 123.64, 123.02, 110.60, 51.14, 31.40, 21.08, 15.27, 12.78; **HRMS**-(ESI): *m/z* $[M+Na]^+$ calcd for $C_{26}H_{24}N_2O_3SNa$ (467.14); Anal calcd for: $C_{26}H_{24}N_2O_3S$ (444.55): C, 70.25; H, 5.44; N, 6.30; S, 7.21; found: C, 70.12; H, 5.22; N, 6.21; S, 7.01.

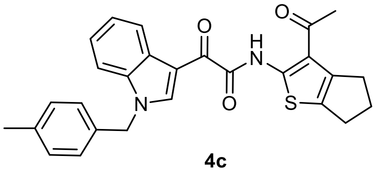

4c

N-(3-Acetyl-5,6-dihydro-4*H*-cyclopenta[b]thiophen-2-yl)-2-(1-(4-methylbenzyl)-1*H*-indol-3-yl)-2-oxo-acetamide (**EE-119, 4c**). Yield (0.37g, 60%); mp 233-5°C; **^{1}H-NMR** (400 MHz, $CDCl_3$) δ 13.40 (s, 1H), 9.11 (s, 1H), 8.56 (d, *J* = 7.8 Hz, 1H), 7.48 – 7.02 (m, 9H), 5.38 (s, 2H), 3.11 – 2.90 (m, 2H), 2.54 (d, *J* = 4.9 Hz, 2H), 2.34 (s, 3H), 1.59 (s, 3H); **^{13}C-NMR** (101 MHz, $CDCl_3$) δ 195.54, 177.38, 160.21, 150.40, 141.11, 141.00, 138.11, 136.56, 134.18, 132.34, 129.72, 127.93, 126.98, 124.13, 123.64, 123.02, 119.02, 112.44, 110.62, 51.13, 31.36, 29.98, 28.73, 28.12, 21.08; Anal calcd for $C_{27}H_{24}N_2O_3S$ (456.56): C, 71.03; H, 5.30; N, 6.14; S, 7.02; found: C, 71.00; H, 5.22; N, 6.24; S, 6.98.

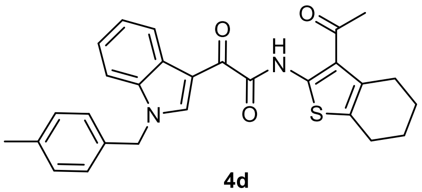

4d

N-(3-Acetyl-4,5,6,7-tetrahydrobenzo[b]thiophen-2-yl)-2-(1-(4-methylbenzyl)-1*H*-indol-3-yl)-2-oxo-acetamide (**EE-69, 4d**). Yield (0.53g, 84%); mp 177-9°C; **^{1}H-NMR** (400 MHz, $CDCl_3$) δ 13.53 (s, 1H), 9.10 (s, 1H), 8.56 (d, *J* = 7.8 Hz, 1H), 7.39 – 6.91 (m, 7H), 5.37 (s, 2H), 2.33 (d, *J* = 15.8 Hz, 2H), 2.64 (d, *J* = 15.8 Hz, 2H), 2.57 (s, 3H), 2.37 (s, 3H), 1.97 – 1.79 (m, 4H); **^{13}C-NMR** (101 MHz, $CDCl_3$) δ 196.27, 177.56, 160.36, 146.11, 141.05, 138.11, 136.57, 132.35, 130.35, 129.71, 128.51, 127.95, 127.00, 124.09, 123.61, 123.23, 123.03, 112.48, 110.57, 51.12, 31.48, 27.47, 24.66, 23.04, 22.63, 21.05; **HRMS**-(ESI): *m/z* $[M+Na]^+$ calcd for $C_{28}H_{26}N_2O_3SNa$ (493.15); Anal calcd for $C_{28}H_{26}N_2O_3S$ (470.59): C, 71.47; H, 5.57; N, 5.95; S, 6.81; found: C, 71.32; H, 5.48; N, 5.87; S, 6.79.

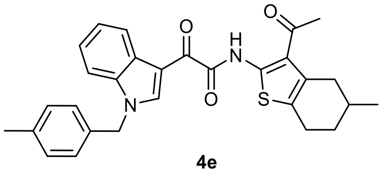

4e

N-(3-Acetyl-5-methyl-4,5,6,7-tetrahydrobenzo[*b*]thiophen-2-yl)-2-(1-(4-methylbenzyl)-1*H*-indol-3-yl)-2-oxo-acetamide (**EE-70, 4e**). Yield (0.40g, 61%); mp 208-10°C; **^{1}H-NMR** (400 MHz, $CDCl_3$) δ 13.55 (s, 1H), 9.11 (d, *J* = 9.5 Hz, 1H), 8.56 (d, *J* = 7.9 Hz, 1H), 7.42 – 7.05 (m, 7H), 5.37 (s, 2H), 2.96 (dt, *J* = 34.9, 17.4 Hz, 1H), 2.83 – 2.67 (m, 2H), 2.58 (s, 3H), 2.46 – 2.32 (m, 1H), 2.35 (s, 3H), 2.05 – 1.82 (m, 1H), 1.63 – 1.44 (m, 2H), 1.16 (s, 3H); **^{13}C-NMR** (101 MHz, $CDCl_3$) δ 196.33, 177.53, 160.33, 146.34, 141.09, 138.11, 136.55, 132.34, 130.41, 129.72, 128.23, 127.93, 127.00, 124.11, 123.63, 123.10, 123.02, 112.46, 110.60, 51.13, 35.96, 31.58, 30.73, 29.30, 24.44, 21.76, 21.08; **HRMS**-(ESI): *m/z* $[M+Na]^+$ calcd for $C_{29}H_{28}N_2O_3SNa$ (507.16); Anal calcd for $C_{29}H_{28}N_2O_3S$ (484.61): C, 71.88; H, 5.82; N, 5.78; S, 6.62; found: C, 71.89; H, 5.77; N, 5.81; S, 6.59.

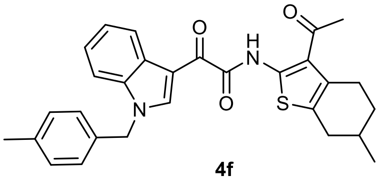

4f

N-(3-Acetyl-6-methyl-4,5,6,7-tetrahydrobenzo[*b*]thiophen-2-yl)-2-(1-(4-methylbenzyl)-1*H*-indol-3-yl)-2-oxo-acetamide (**EE-71, 4f**). Yield (0.58g, 88%); mp 176-8°C; **^{1}H-NMR** (400 MHz, $CDCl_3$) δ 13.55 (s, 1H), 9.11 (s, 1H), 8.56 (d, *J* = 7.9 Hz, 1H), 7.44 – 7.09 (m, 7H), 5.37 (s, 2H), 3.00 (dd, *J* = 26.9, 15.0 Hz, 1H), 2.84 – 2.70 (m, 2H), 2.58 (d, *J* = 2.9 Hz, 3H), 2.36 (d, *J* = 12.1 Hz, 1H), 2.25 – 2.13 (m, 1H), 1.95 (t, *J* = 26.2 Hz, 2H), 1.66 – 1.56 (m, 2H), 1.13 (d, *J* = 6.5 Hz, 1H), 0.99 (s, 3H); ^{13}C NMR (101 MHz, $CDCl_3$) δ 196.32, 196.28, 177.52, 160.35, 146.29, 146.22, 141.10, 138.11, 136.55, 132.34, 130.39, 130.05, 129.72, 129.29, 128.27, 127.94, 127.00, 124.11, 123.63, 123.02, 122.84, 112.46, 110.60, 51.13, 44.67, 32.75, 32.41, 31.51, 31.42, 31.24, 28.95, 28.62, 27.27, 27.24, 26.17, 24.69, 21.28, 21.08; Anal calcd for $C_{29}H_{28}N_2O_3S$ (484.61): C, 71.88; H, 5.82; N, 5.78; S, 6.62; found: C, 71.89; H, 5.77; N, 5.81; S, 6.59.

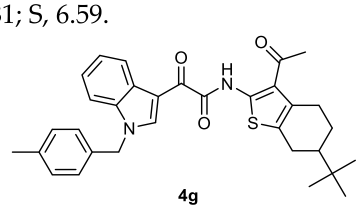

4g

N-(3-Acetyl-6-(tert-butyl)-4,5,6,7-tetrahydrobenzo[*b*]thiophen-2-yl)-2-(1-(4-methyl-benzyl)-1*H*-indol-3-yl)-2-oxo-acetamide (**EE-72, 4g**). Yield (0.41g, 58%); mp 166-8°C; **^{1}H-NMR** (400 MHz, $CDCl_3$) δ 13.54 (s, 1H),

9.10 (s, 1H), 8.52 (s, 1H), 7.43 – 7.08 (m, 7H), 5.38 (s, 2H), 3.08 (d, *J* = 15.7 Hz, 2H), 2.77 (d, *J* = 15.7 Hz, 2H), 2.54 (s, 3H), 2.34 (s, 3H), 1.45 (m, 3H), 1.01 (s, 9H); **^{13}C-NMR** (101 MHz, $CDCl_3$) δ 196.26, 160.35, 146.29, 141.09, 138.11, 136.56, 132.35, 130.38, 129.72, 129.29, 127.95, 126.98, 124.11, 123.62, 123.03, 122.85, 112.47, 110.59, 51.13, 44.68, 32.41, 31.40, 28.63, 27.24, 26.17, 24.70, 21.07; Anal calcd for $C_{32}H_{34}N_2O_3S$ (526.70): C, 72.97; H, 6.51; N, 5.32; S, 6.09; found: C, 73.00; H, 6.49; N, 5.21; S, 5.99.

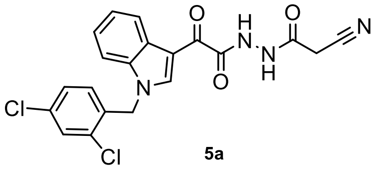

5a

N'-(2-cyanoacetyl)-2-(1-(2,4-dichlorobenzyl)-1*H*-indol-3-yl)-2-oxoacetohydrazide (**EE-73, 5a**). Yield (0.36g, 77%); mp 256-8°C; **^{1}H-NMR** (400 MHz, DMSO-*d$_6$*) δ 10.81 (s, 1H), 10.41 (s, 1H), 8.89 (s, 1H), 8.21 (s, 1H), 7.66 (d, *J* = 8.8 Hz, 1H), 7.35 (d, *J* = 8.7 Hz, 1H), 7.18 (dd, *J* = 20.3, 8.1 Hz, 4H), 5.56 (s, 2H), 3.83 (s, 2H); **^{13}C-NMR** (101 MHz, $CDCl_3$) δ 194.03, 180.86, 162.03, 161.47, 141.95, 137.40, 135.07, 133.25, 129.46, 129.37, 129.31, 128.14, 127.86, 127.56, 127.47, 123.90, 120.66, 115.55, 113.56, 111.24, 69.79, 50.04, 45.84; Anal calcd for $C_{20}H_{14}Cl_2N_4O_3$ (429.26): C, 55.96; H, 3.29; Cl, 16.52; N, 13.05; found: C, 55.88; H, 3.21; N, 12.99.

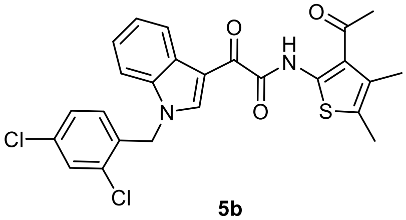

5b

N-(3-Acetyl-4,5-dimethylthiophen-2-yl)-2-(1-(2,4-dichlorobenzyl)-1*H*-indol-3-yl)-2-oxo-acetamide (**EE-74, 5b**). Yield (0.39g, 72%); 227-9°C; **^{1}H-NMR** (400 MHz, $CDCl_3$) δ 13.47 (s, 1H), 9.09 (s, 1H), 8.58 (d, *J* = 7.9 Hz, 1H), 7.52 – 7.09 (m, 5H), 6.73 (d, *J* = 8.3 Hz, 1H), 5.49 (s, 2H), 2.62 (s, 3H), 2.37 (d, *J* = 10.0 Hz, 6H); ^{13}C NMR (101 MHz, $CDCl_3$) δ 196.72, 177.73, 160.07, 144.92, 141.04, 136.35, 134.83, 133.36, 131.69, 129.73, 129.06, 128.49, 127.81, 127.70, 125.36, 124.46, 123.89, 123.11, 112.87, 110.26, 45.86, 31.39, 8.63; **HRMS**-(ESI): *m/z* $[M+Na]^+$ calcd for $C_{25}H_{20}Cl_2N_2O_3SNa$ (521.04); Anal calcd for $C_{25}H_{20}Cl_2N_2O_3S$ (499.41): C, 60.13; H, 4.04; N, 5.61; S, 6.42; found: C, 60.01; H, 4.00; N, 5.54; S, 6.32.

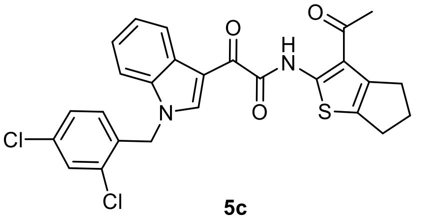

5c

N-(3-acetyl-5,6-dihydro-4H-cyclopenta[b]thiophen-2-yl)-2-(1-(2,4-dichlorobenzyl)-1H-indol-3-yl)-2-oxo-acetamide (**EE-75, 5c**). Yield (0.31g, 54%); mp 239-241°C; **^{1}H-NMR** (400 MHz, $CDCl_3$) δ 13.41 (s, 1H), 9.10 (s, 1H), 8.60 (s, 1H), 7.59 – 7.11 (m, 5H), 6.73 (d, *J* = 15.9 Hz, 1H), 5.50 (s, 2H), 3.13 – 2.97 (m, 2H), 2.95 (t, *J* = 12.3 Hz, 2H), 2.54 (s, 3H), 2.52 (d, *J* = 9.7 Hz, 1H), 2.21 (s, 1H); **HRMS**-(ESI): *m/z* $[M+Na]^+$ calcd for $C_{26}H_{20}Cl_2N_2O_3SNa$ (533.04); Anal calcd for $C_{26}H_{20}Cl_2N_2O_3S$ (511.42): C, 61.06; H, 3.94; N, 5.48; S, 6.27; found: C, 61.22; H, 4.01; N, 5.35; S, 6.12.

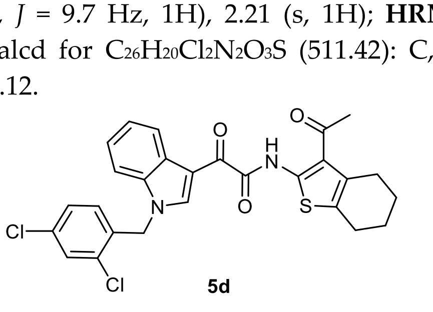

5d

N-(3-Acetyl-4,5,6,7-tetrahydrobenzo[*b*]thiophen-2-yl)-2-(1-(2,4-dichlorobenzyl)-1*H*-indol-3-yl)-2-oxo-acetamide (**EE-76, 5d**). Yield (0.36g, 63%); mp 189-91°C; **^{1}H-NMR** (400 MHz, $CDCl_3$) δ 13.56 (s, 1H), 9.06 (s, 1H), 8.58 (d, *J* = 7.7 Hz, 1H), 7.34 (dt, *J* = 70.1, 43.8 Hz, 5H), 6.72 (d, *J* = 13.8 Hz, 1H), 5.49 (s, 2H), 2.86 (d, *J* = 9.7 Hz, 2H), 2.74 (d, *J* = 9.7 Hz, 2H), 2.58 (s, 3H), 1.89 (t, 3H), 1.59 (s, 1H); **^{13}C-NMR** (101 MHz, $CDCl_3$) δ 196.40, 177.74, 160.13, 146.00, 141.02, 136.34, 134.83, 133.34, 131.71, 130.41, 129.74, 129.03, 128.65, 127.83, 127.74, 124.46, 123.91, 123.27, 123.18, 112.91, 110.23, 48.16, 31.55, 27.48, 24.67, 23.02, 22.61; **HRMS**-(ESI): *m/z* $[M+Na]^+$ calcd for $C_{27}H_{22}Cl_2N_2O_3SNa$ (547.06); Anal calcd for $C_{27}H_{22}Cl_2N_2O_3S$ (525.44): C, 61.72; H, 4.22; N, 5.33; S, 6.10; found: C, 61.66; H, 4.32; N, 5.21; S, 6.23.

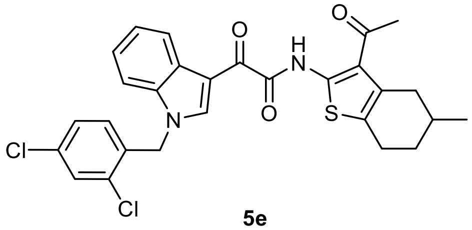

5e

N-(3-Acetyl-5-methyl-4,5,6,7-tetrahydrobenzo[*b*]thiophen-2-yl)-2-(1-(2,4-dichlorobenzyl) -1*H*-indol-3-yl)-2-oxo-acetamide (**EE-77, 5e**). Yield (0.47g, 82%); mp 283-5°C; **^{1}H-NMR** (400 MHz, $CDCl_3$) δ 13.55 (s, 1H), 9.09 (s, 1H), 8.58 (d, *J* = 7.8 Hz, 1H), 7.55 – 7.19 (m, 4H), 7.14 (dd, *J* = 8.3, 2.0 Hz, 1H), 6.73 (d, *J* = 8.4 Hz, 1H), 5.46 (s, 2H), 2.97 (d, *J* = 12.1 Hz, 1H), 2.86 – 2.67 (m, 2H), 2.58 (s, 3H), 2.44 – 2.27 (m, 2H), 1.98 (dd, *J* = 29.4, 21.0 Hz, 1H), 1.48 (dd, *J* = 8.8, 4.4 Hz, 1H), 1.13 (s, 3H); **^{13}C-NMR** (101 MHz, $CDCl_3$) δ 196.26, 177.35, 159.67, 145.75, 141.04, 136.10, 134.06, 131.72, 129.74, 129.48, 128.27, 124.47, 123.92, 123.39, 113.13, 110.24, 48.09, 36.18, 31.81, 30.72, 29.48, 24.29, 21.95; Anal calcd for $C_{28}H_{24}Cl_2N_2O_3S$ (539.47): C, 62.34; H, 4.48; N, 5.19; S, 5.94; found: C, 62.22; H, 4.35; N, 5.01; S, 6.00

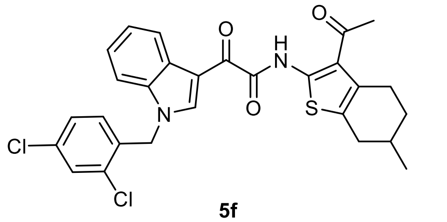

5f

N-(3-Acetyl-6-methyl-4,5,6,7-tetrahydrobenzo[*b*]thiophen-2-yl)-2-(1-(2,4-dichlorobenzyl) -1*H*-indol-3-yl)-2-oxo-acetamide (**EE-78, 5f**). Yield (0.44g, 75%); mp 206-8°C; **^{1}H-NMR** (400 MHz, $CDCl_3$) δ 13.56 (s, 1H), 9.09 (m, 1H), 8.58 (s, 1H), 7.50 – 7.27 (m, 4H), 7.15-7.13 (d, *J* = 6.0 Hz, 1H), 6.72 (d, *J* = 7.7 Hz, 1H), 5.48 (d, *J* = 2.8 Hz, 2H), 2.97 (d, *J* = 14.7 Hz, 1H), 2.82 (d, *J* = 16.3 Hz, 2H), 2.58 (d, *J* = 2.5 Hz, 3H), 2.37 (t, 1H), 1.98 (s, 1H), 1.56 (t, *J* = 17.4 Hz, 2H), 1.13 (s, 3H); **^{13}C-NMR** (101 MHz, $CDCl_3$) δ 196.41, 177.73, 160.14, 146.13, 141.03, 136.34, 134.83, 133.33, 131.71, 130.09, 129.74, 129.01, 128.41, 127.83, 127.74, 124.47, 123.92, 123.18, 123.09, 112.91, 110.23, 48.16, 32.76, 31.53, 31.22, 28.94, 27.27, 21.28; **HRMS**-(ESI): *m/z* $[M+Na]^+$ calcd for $C_{28}H_{24}Cl_2N_2O_3SNa$ (561. 08); Anal calcd for $C_{28}H_{24}Cl_2N_2O_3S$ (539.47): C, 62.34; H, 4.48; N, 5.19; S, 5.94; found: C, 62.45; H, 4.55; N, 5.21; S, 6.09

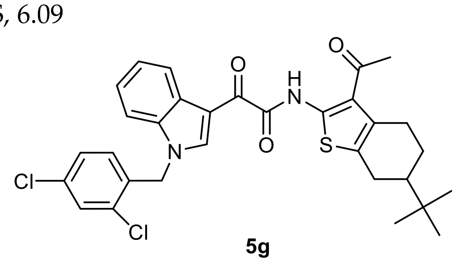

5g

N-(3-Acetyl-6-(tert-butyl)-4,5,6,7-tetrahydrobenzo[*b*]thiophen-2-yl)-2-(1-(2,4-dichloro-benzyl)-1*H*-indol-3-yl)-2-oxo-acetamide (**EE-79, 5g**). Yield (0.32g, 51%); mp 241-3°C; **^{1}H-NMR** (400 MHz, $CDCl_3$) δ 13.55 (s, 1H), 9.10 (s, 1H), 8.58 (d, *J* = 7.7 Hz, 1H), 7.47 (t, *J* = 14.8 Hz, 1H), 7.43 – 7.19 (m, 3H), 7.15 (dd, *J* = 8.4, 2.1

Hz, 1H), 6.72 (d, *J* = 8.4 Hz, 1H), 5.49 (s, 2H), 3.12 – 2.95 (m, 1H), 2.84 – 2.69 (m, 2H), 2.58 – 2.39 (m, 4H), 2.14 (d, *J* = 9.4 Hz, 1H), 1.68 – 1.30 (m, 2H), 0.99 (s, 9H); **^{13}C-NMR** (101 MHz, $CDCl_3$) δ 196.35, 177.76, 160.11, 146.20, 141.03, 136.35, 134.83, 133.32, 131.73, 130.42, 129.74, 129.44, 129.00, 127.83, 127.75, 124.46, 123.92, 123.19, 122.91, 112.92, 110.23, 48.16, 44.67, 32.41, 31.43, 28.62, 27.24, 26.18, 24.68; **HRMS**-(ESI): *m*/*z* $[M+Na]^+$ calcd for $C_{31}H_{30}Cl_2N_2O_3SNa$ (603.12); Anal calcd for $C_{31}H_{30}Cl_2N_2O_3S$ (581.55): C, 64.03; H, 5.20; N, 4.82; S, 5.51; found: C, 64.21; H, 5.32; N, 4.98; S, 5.46

Table S1. The cytotoxicity of aplysinopsin (**10**) and its analogs **3**c-g, **4**b-g and **5**a-g against different cell lines. The data are represented as cell death percent relative to doxorubicin at 100ppm for 48 h. The data are expressed as mean ±SD

Compounds No.	HCT-116	MCF-7	PC3	A549	HepG2
3c	NA	NA	91.03±6.8	NA	NA
3d	NA	NA	79.6±3.2	NA	NA
3e	NA	NA	99.06±0.56	**75.2±8.2**	NA
3f	NA	NA	34±4.9	NA	NA
3g	NA	NA	NA	NA	NA
4b	NA	NA	99.5±1.1	NA	NA
4c	NA	NA	98.6±1.5	NA	NA
4d	NA	NA	87.21±4.9	NA	NA
4e	NA	NA	87.86±8.8	NA	NA
4f	NA	NA	65.8±5.4	NA	NA
4g	NA	NA	61±0.98	NA	NA
5a	NA	76.2±2.5	91.31±8.8	76±2.5	NA
5b	NA	NA	99.56±0.25	NA	NA
5c	NA	NA	81.35±1.1	NA	NA
5d	NA	NA	91.89±6.3	NA	NA
5e	NA	NA	NA	NA	NA
5f	NA	NA	84.7±5.5	NA	NA
5g	NA	NA	NA	NA	NA
Aplysinopsin 10	NA	NA	100.16±0.3	NA	99.2±0.2

Table S2. The IC_{50} of aplysinopsin (**10**) and its analogs **3**c-g, **4**b-g and **5**a-g against PC3 cell line. The data are expressed as mean ±SD

Compounds No.	IC_{50} μM	Compounds No.	IC_{50} μM
3c	0.133±0.17	4f	0.151±0.13
3d	0.113±0.12	4g	0.154±0.05
3e	0.086±0.08	5a	0.056±0.3
3f	ND	5b	0.079±0.24
3g	ND	5c	0.103±0.025
4b	0.037±0.43	5d	0.112±0.1
4c	0.073±0.15	5e	ND
4d	0.090±0.074	5f	0.098±0.8
4e	0.075±0.37	5g	ND
Aplysinopsin (**10**)	0.107±0.38		

ND: not detected

Table S3. Apoptosis phase after treatment of PC3 cells with Aplysinopsin analogs for 24h. Results are represented as the mean of three independent experiments.

Sample	Early apoptosis	Late apoptosis	Necrosis	Total cell death
untreated	0.69±0.37	0.3± 0.22	1.48±0.37	2.48±1.32
Aplysinopsin (10)	13.08±3.13	8.64± 2.89	4.84± 2.63	26.56±3.96
4b	12.42±5.91	13.99±7.53	4.66± 2.62	31.07± 7.55
4c	14.17± 5.10	23.24±8.78	5.66± 3.17	43.07±7.02
4e	9.81±3.74	11.41±3.29	7.49±3.46	28.71±3.89
5a	8.61±0.49	16.01±3.98	11.53±3.53	36.15±6.04
5b	9.68±3.78	9.53±2.56	7.22±2.40	26.43±4.36

Table S4. Gene expression analysis (expressed as fold Change B-actin) of active compounds on Prostate cancer cell line. Values are expressed as Mean±SD

**Treatment**	**Gene expression (Fold change)**			
	**P53**	**Caspase-3**	**Bax**	**Bcl-2**
untreated	1.101±0.042	1.012±0.039	1.110±0.210	1.023±0.121
Doxorubicin	3.934±0.313	5.032±0.214	3.234±0.104	0.121±0.048
Aplysinopsin (10)	1.863±0.122	1.243±0.121	1.671±0.114	0.917±0.065
4b	2.154±0.146	3.762±0.212	2.765±0.094	0.443±0.113
4c	2.012±0.241	1.981±0.123	2.876±0.106	0.456±0.123
4e	1.321±0.231	1.542±0.123	1.353±0.107	0.972±0.016
5a	1.764±0.124	2.167±0.211	1.985±0.121	0.345±0.054
5b	1.623±0.211	1.4123±0.217	1.541±0.132	0.756±0.073

Table S5. Cell cycle phase distribution after treatment of PC3 cells with Aplysinopsin analogs for 24h. Results are represented as the mean of three independent experiments.

**Sample**	**% G0-G1**	**% S**	**% G2/M**	**% Pre-G1**
untreated	51.39±15.49	28.66±5.72	19.95±8.07	2.48±2.19
Aplysinopsin (10)	55.91±7.78	27.44±4.86	16.65±3.84	26.56±7.07
4b	44.36±10.19	39.51±8.78	16.13±1.67	31.07±2.96
4c	57.12±3.32	33.89±3.27	8.99±1.99	43.07±2.78
4e	59.84±8.72	26.58±6.17	13.58±3.28	28.71±6.38
5a	47.58±4.79	43.41±6.15	9.01±4.41	36.15±7.12
5b	52.94±10.19	40.76±8.78	6.3±1.67	26.43±2.96

Table S6. The molecular docking result of aplysinopsin (**10**), and its analogs **4b**, **4c**, **4e**, **5a**, and **5b**

Comp. No.	Score Kcal/mol	Moieties from the compound	Amino acid residues	Type of interaction
LBM	-12.6	C=O	GLN99, ASN143	Conventional H-bond
		O=S=O	GLY145	
		NH of pyrrole	ASP103	
		C=C of pyrrole	ALA100	
		N of piprazine	ASP111	Attractive charge
		Phenyl	PHE104	Pi-Pi
		Pyran (C-O-C)	TYP202	
		Phenyl	ARG146, ALA149, MET115	Pi-Alkyl
		Pyrrole	VAL148	
		CH_3	VAL156, PHE112	
**4b**	-8.3	$COCH_3$	ARG107	Conventional H-bond
		C=O	ARG107	
		NH	ARG207	
		C=C of pyrrole ring	ARG207	
		Pyrrole ring	ARG207, ASP103	Attractive charge
		Phenyl	ASP103	
		Phenyl	TRY202	Pi-Pi
		CH_3	TRY108, VAL148	Pi-Alkyl
		Phenyl	VAL148	
		Phenyl	ALA100	Pi-sigma
**4c**	-8.7	NH	ARG207	Conventional H-bond
		$COCH_3$	ALA100	
		Thiophene ring	ARG107, APS103, ARG207	Attractive charge
		Pyrrole ring	ASP103	
		Thiophene ring	MET206	Pi-Pi
		Phenyl	TYR202, GLY145	
		Phenyl	ALA100, VAL148	Pi-Alkyl
		Methyl	ARG146	
**4e**	-9.2	$COCH_3$	GLY145	Conventional H-bond
		CH_2	ASP103	
		Phenyl	ARG207	Attractive charge
		Benzopyrrole	TYR202	Pi-Pi
		Thiophene	TYR108	
		CH_3	TYR108, PHE104	Pi-Alkyl
		Hexane	TYR108	
		Phenyl	VAL148, ALA100	
**5a**	-8.9	NH	TYR202	Conventional H-bond
		Cl	ARG107	
		C=O	ALA100	
		NH	ASP103, ARG207	Attractive charge
		Cl	TYR108	Pi-Alkyl
		Phenyl	VAL148, ALA100, TYR202	
		Pyrrol	ALA100, TYR202	
**5b**	-8.5	Phenyl	ASP103, ARG207	Attractive charge
		Phenyl	VAL148, ALA100	Pi-Alkyl
		CH_3	ARG148	
		Phenyl and Pyrrol	TYR202	Pi-Pi
		CH_3	TYR108	Pi-Sigma
**Aplysino**	-8.7	Pyrrole ring	ARG207	Conventional H-bond

**psin (10)**	Imidazolidine	ARG207	Attractive charge
	C=O	ARG207	
	N-CH_3	GLY203	
	Benzopyrrole	TYR202	Pi-Pi
	Phenyl	ALA100, VAL148	Pi-Alkyl
	Pyrrole ring	ALA100	

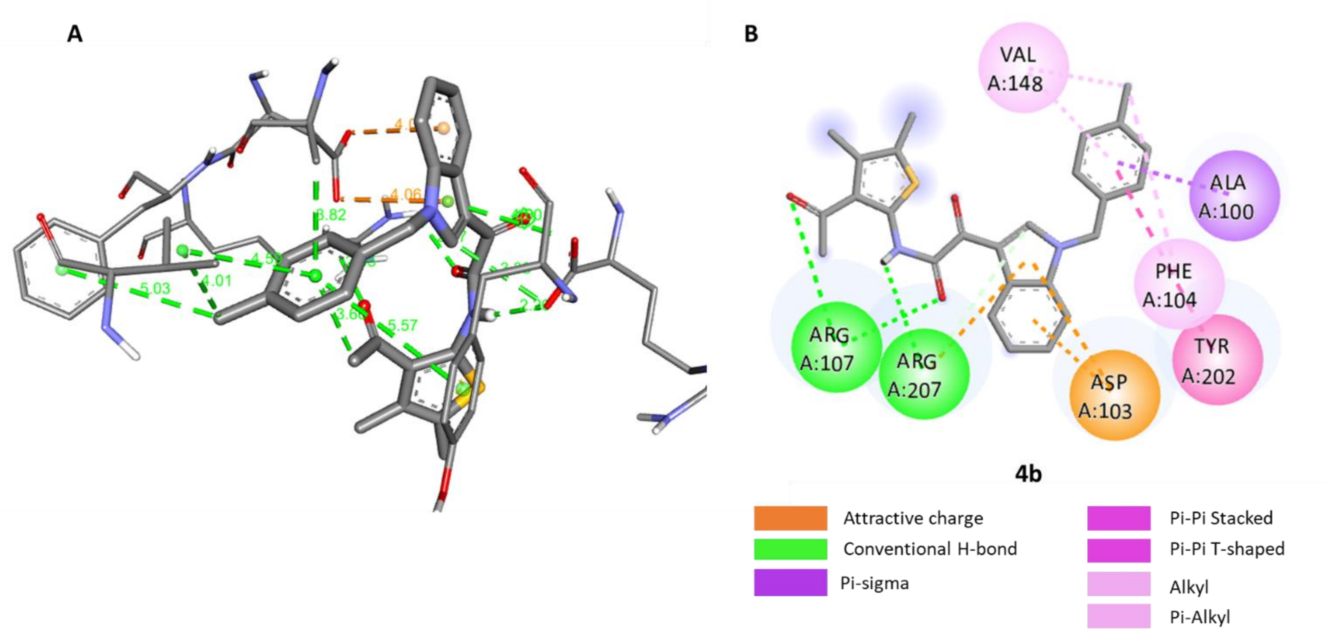

Figure S1. A. The 3D interaction of **4b** inside the binding pocket of BCl2 (PDB: 6O0K). **B.** The 2D interaction of **4b** inside the binding pocket of BCl2 (PDB: 6O0K) illustrating the formed hydrogen bonds, attractive charge, pi-pi bond, and pi-alkyl

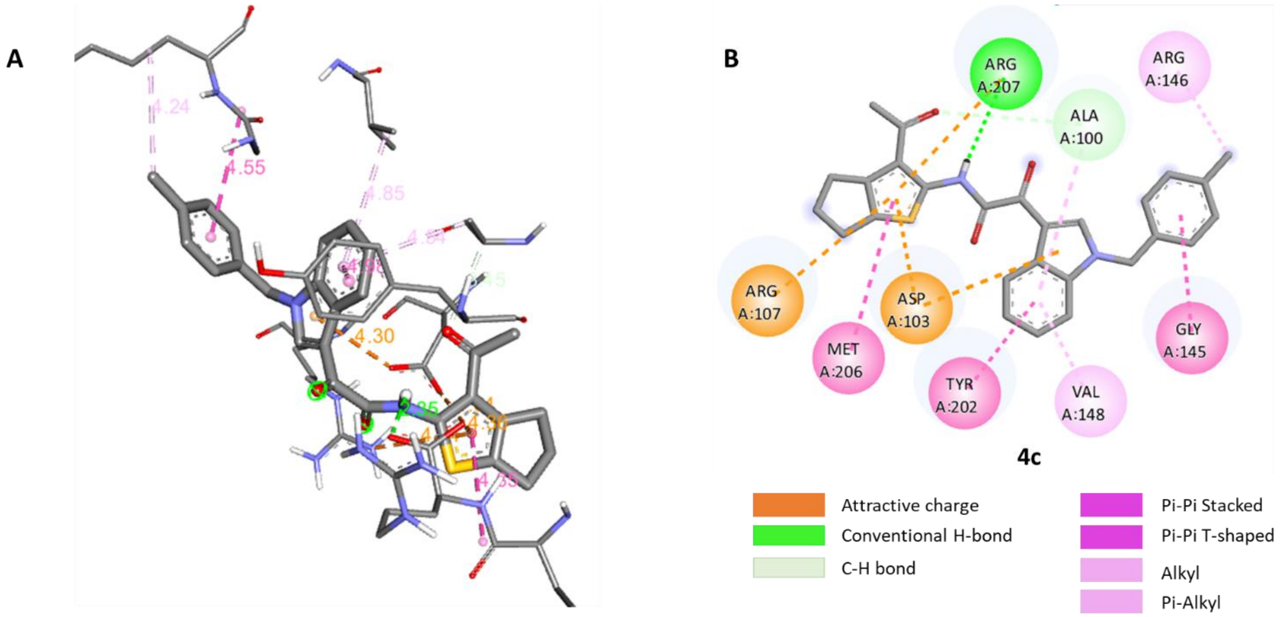

Figure S2. A. The 3D interaction of **4c** inside the binding pocket of BCl2 (PDB: 6O0K). **B.** The 2D interaction of **4c** inside the binding pocket of BCl2 (PDB: 6O0K) illustrating the formed hydrogen bonds, attractive charge, pi-pi bond, and pi-alkyl

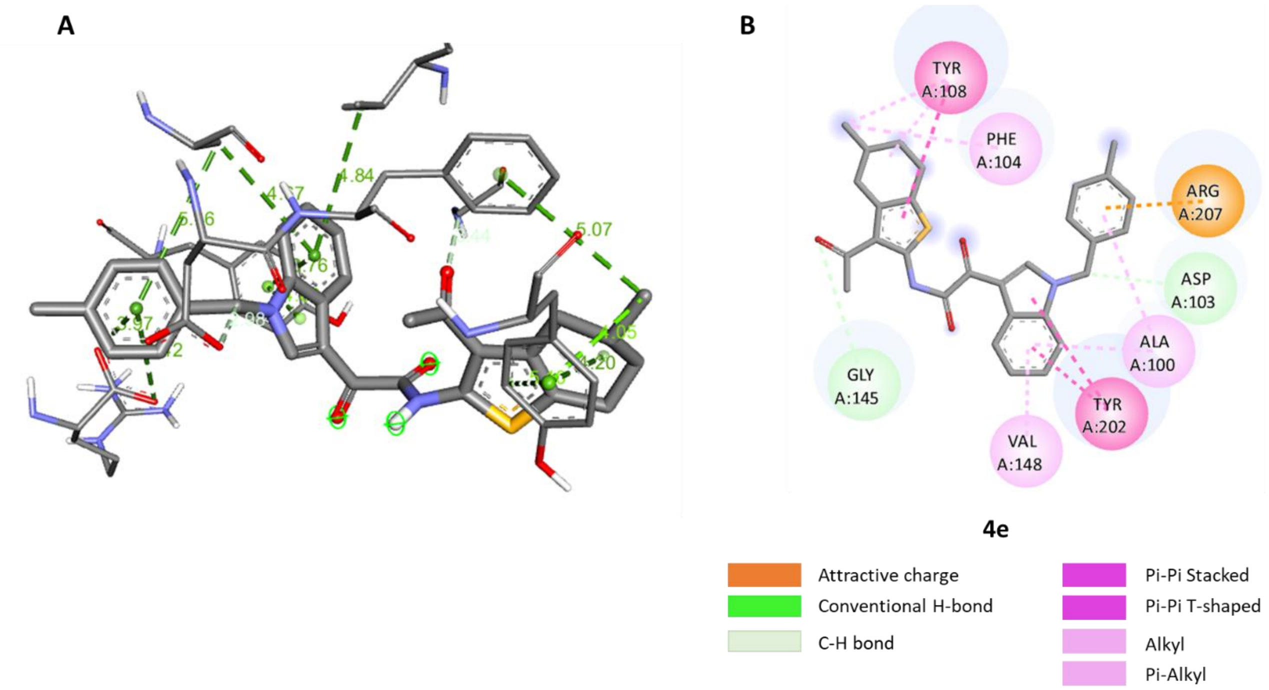

Figure S3. A. The 3D interaction of **4e** inside the binding pocket of BCl2 (PDB: 6O0K). **B.** The 2D interaction of **4e** inside the binding pocket of BCl2 (PDB: 6O0K) illustrating the formed hydrogen bonds, attractive charge, pi-pi bond, and pi-alkyl

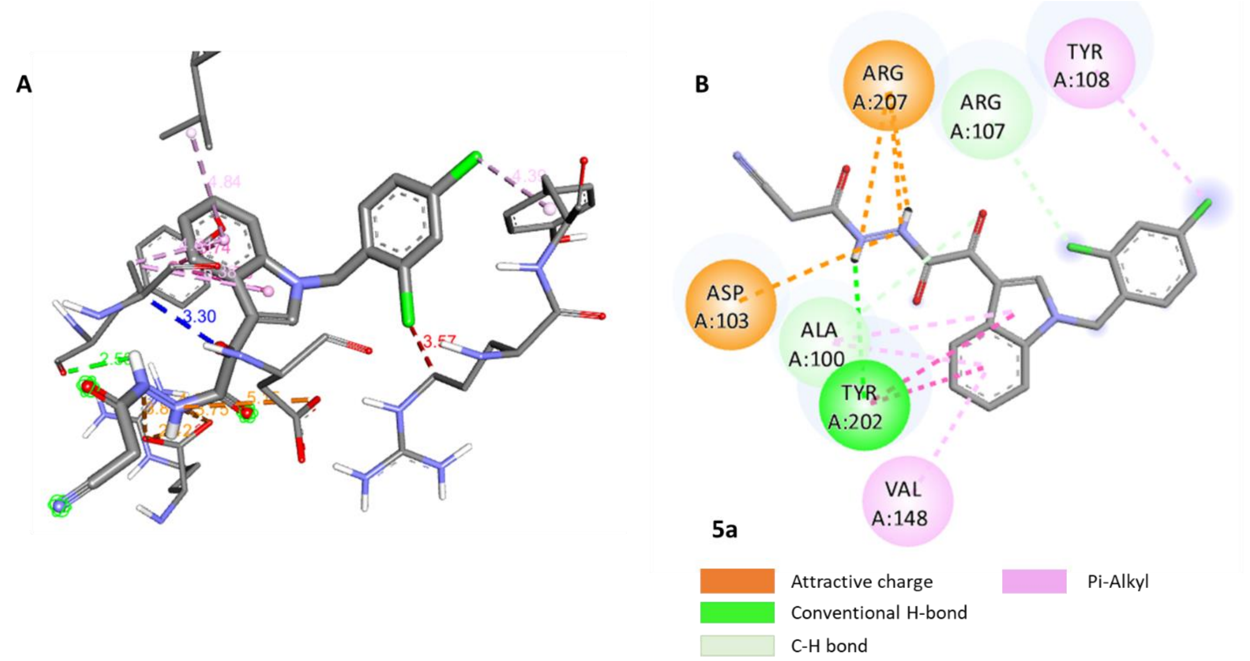

Figure S4. A. The 3D interaction of **5a** inside the binding pocket of BCl2 (PDB: 6O0K). **B.** The 2D interaction of **5a** inside the binding pocket of BCl2 (PDB: 6O0K) illustrating the formed hydrogen bonds, attractive charge, pi-pi bond, and pi-alkyl

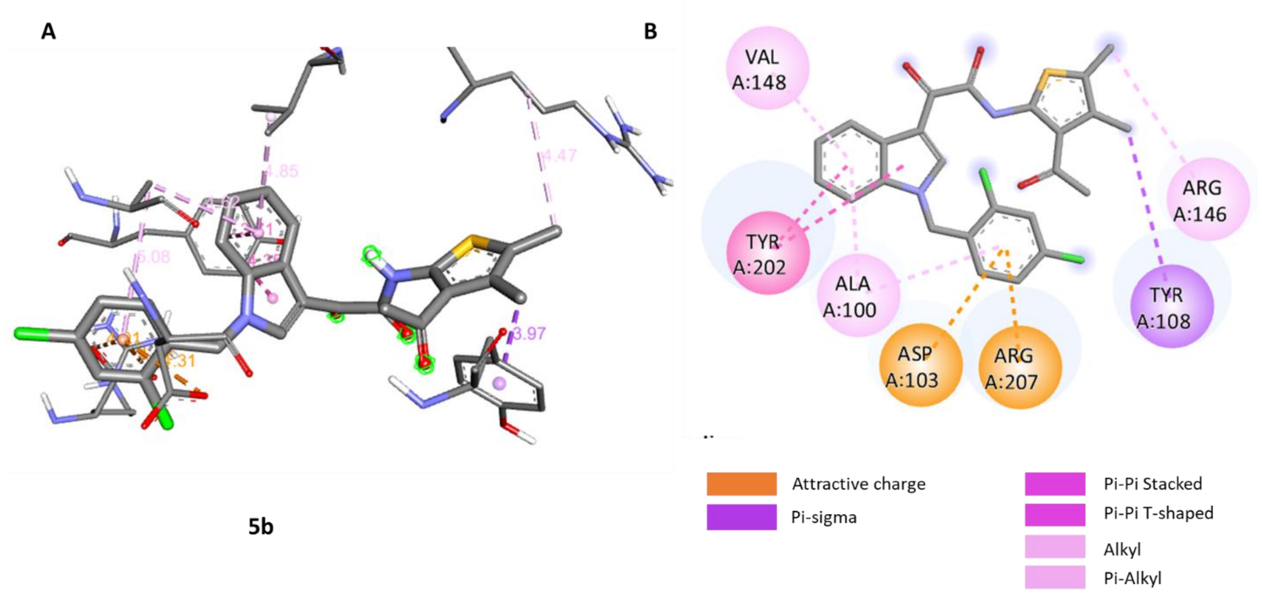

Figure S5. A. The 3D interaction of **5b** inside the binding pocket of BCl2 (PDB: 6O0K). **B.** The 2D interaction of **5b** inside the binding pocket of BCl2 (PDB: 6O0K) illustrating the formed attractive charge, pi-pi bond, and pi-alkyl

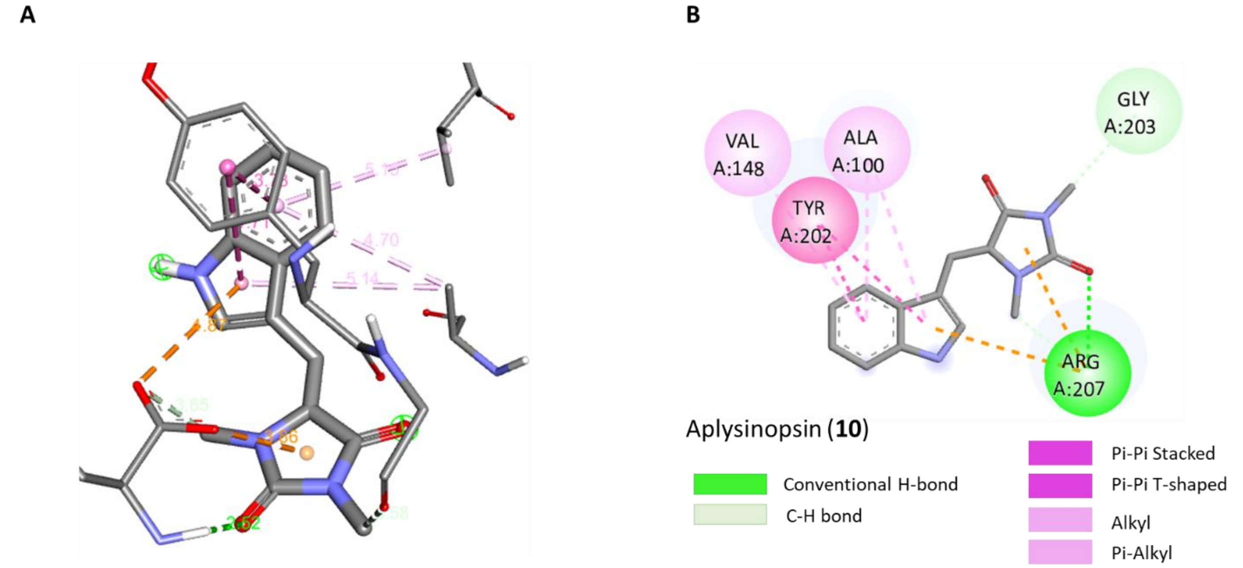

Figure S6. A. The 3D interaction of aplysinopsin (10) inside the binding pocket of BCl2 (PDB: 6O0K). **B.** The 2D interaction of aplysinopsin (10) inside the binding pocket of BCl2 (PDB: 6O0K) illustrating the formed hydrogen bonds, attractive charge, pi-pi bond, and pi-alkyl
